# Supplementary figures and images for: Novel tissue mechanics-guided cellular flows drive the formation of feather follicles (part 1 of 2)
Source: EMBO J. 2026 May 2;45(11):3926–53. doi: 10.1038/s44318-026-00771-7 (PMC13226717; doi:10.1038/s44318-026-00771-7)

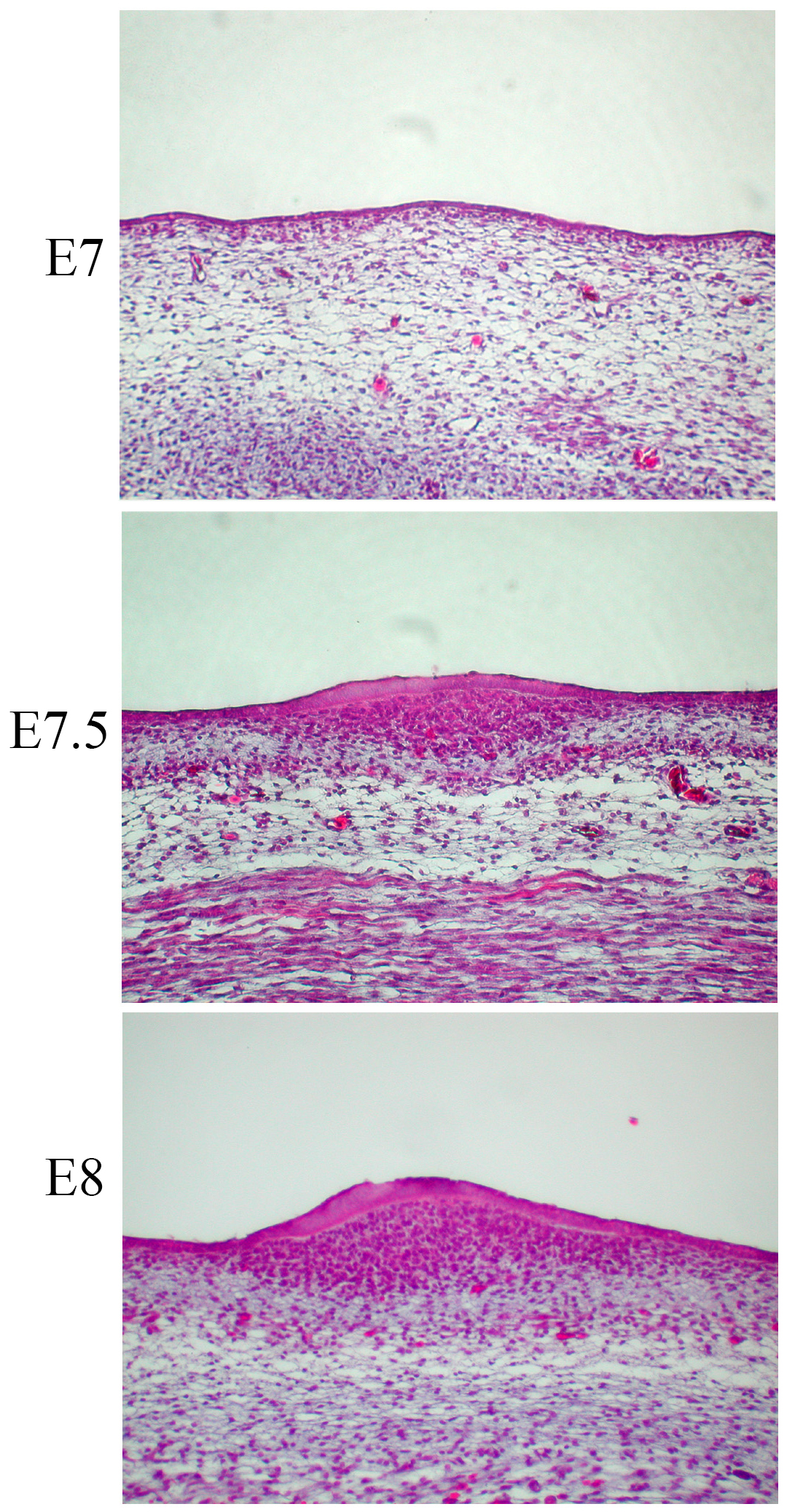

Supplement: Supplementary file 3 — Movie EV1 [file 44318_2026_771_MOESM3_ESM.zip › Fig 1 revised/Fig 1A n1.tif]

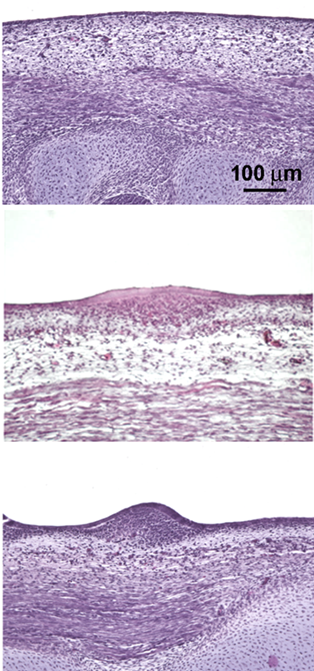

Supplement: Supplementary file 3 — Movie EV1 [file 44318_2026_771_MOESM3_ESM.zip › Fig 1 revised/Fig 1A n2.tif]

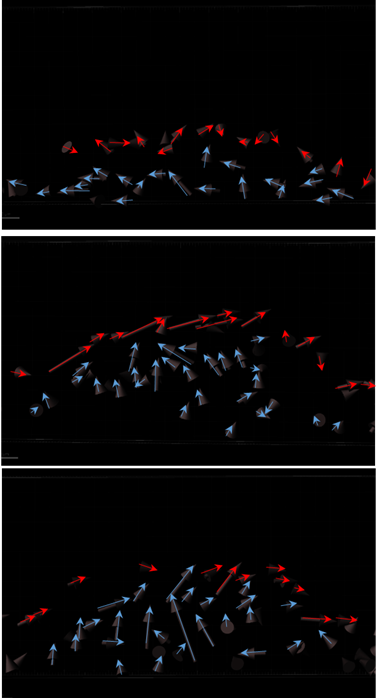

Supplement: Supplementary file 3 — Movie EV1 [file 44318_2026_771_MOESM3_ESM.zip › Fig 1 revised/Fig 1B n1.tif]

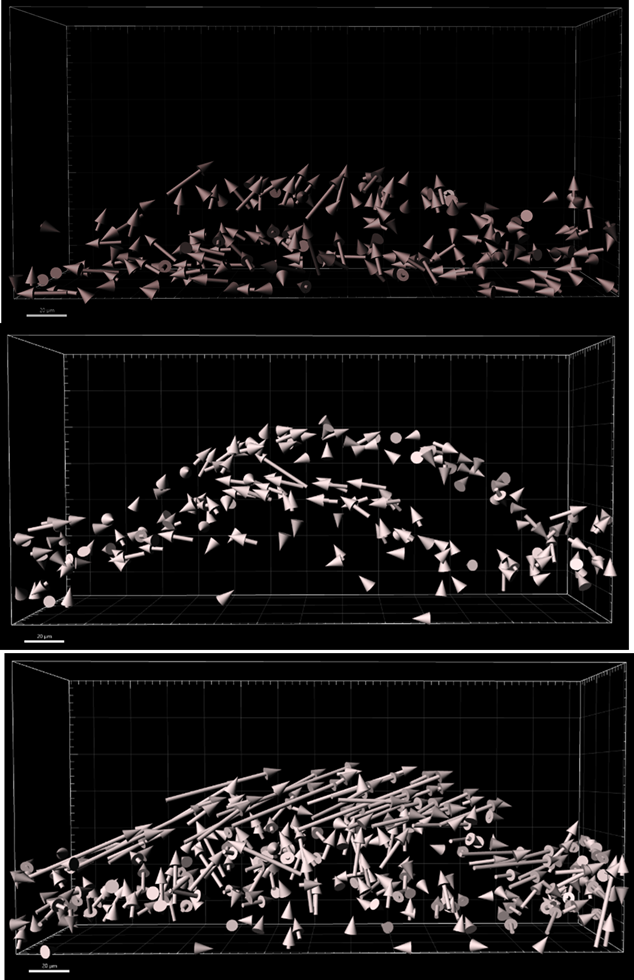

Supplement: Supplementary file 3 — Movie EV1 [file 44318_2026_771_MOESM3_ESM.zip › Fig 1 revised/Fig 1B n2.tif]

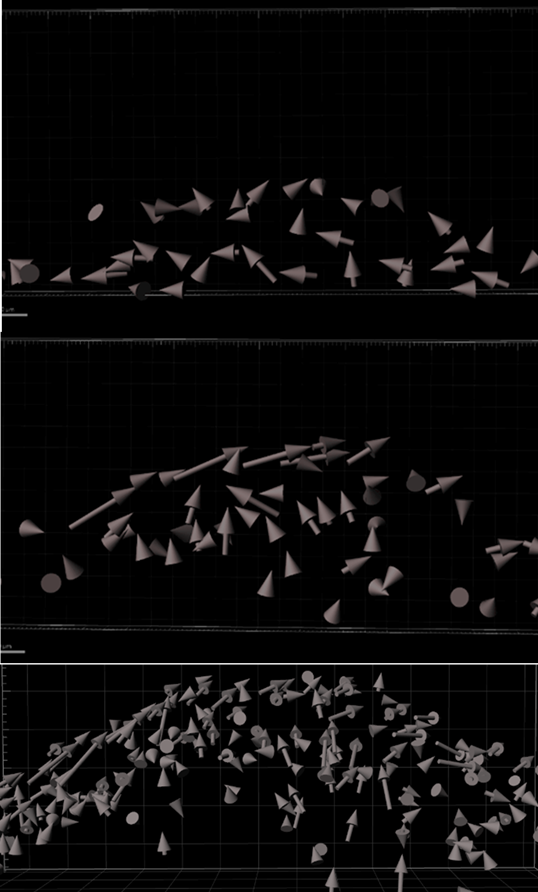

Supplement: Supplementary file 3 — Movie EV1 [file 44318_2026_771_MOESM3_ESM.zip › Fig 1 revised/Fig 1B n3.tif]

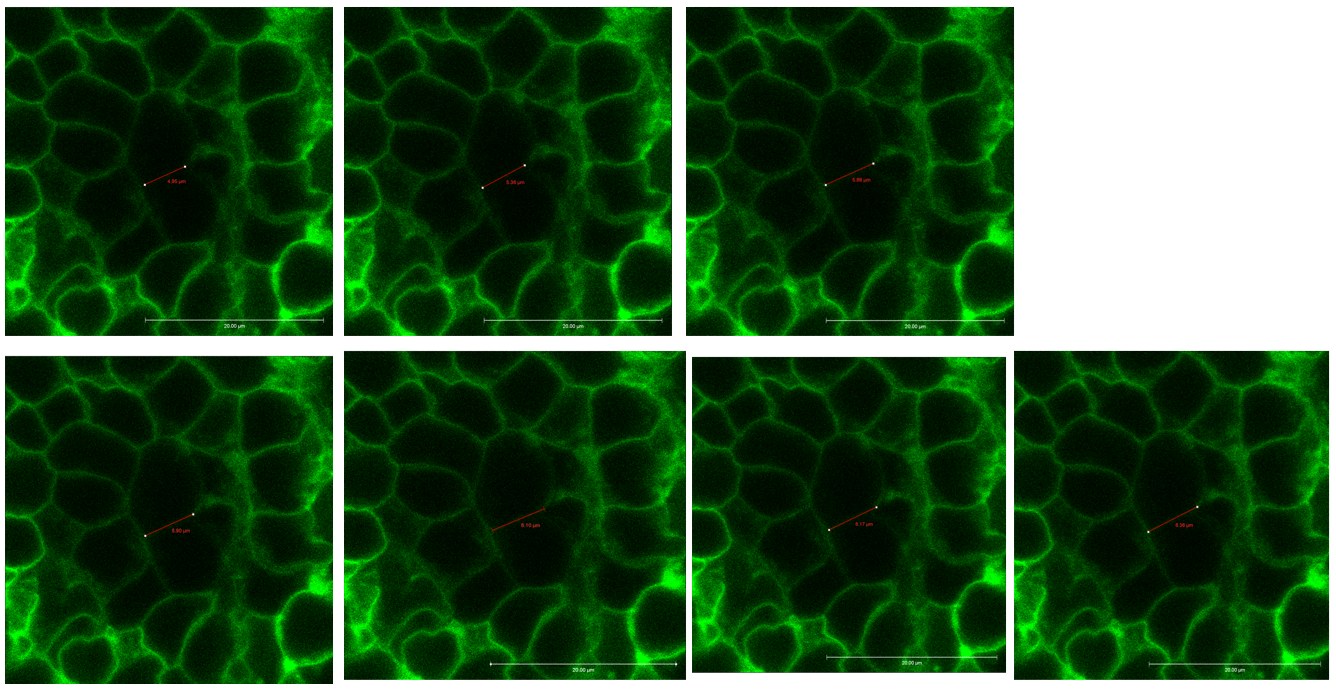

Supplement: Supplementary file 3 — Movie EV1 [file 44318_2026_771_MOESM3_ESM.zip › Fig 1 revised/Fig 1F bud pro n1.tif]

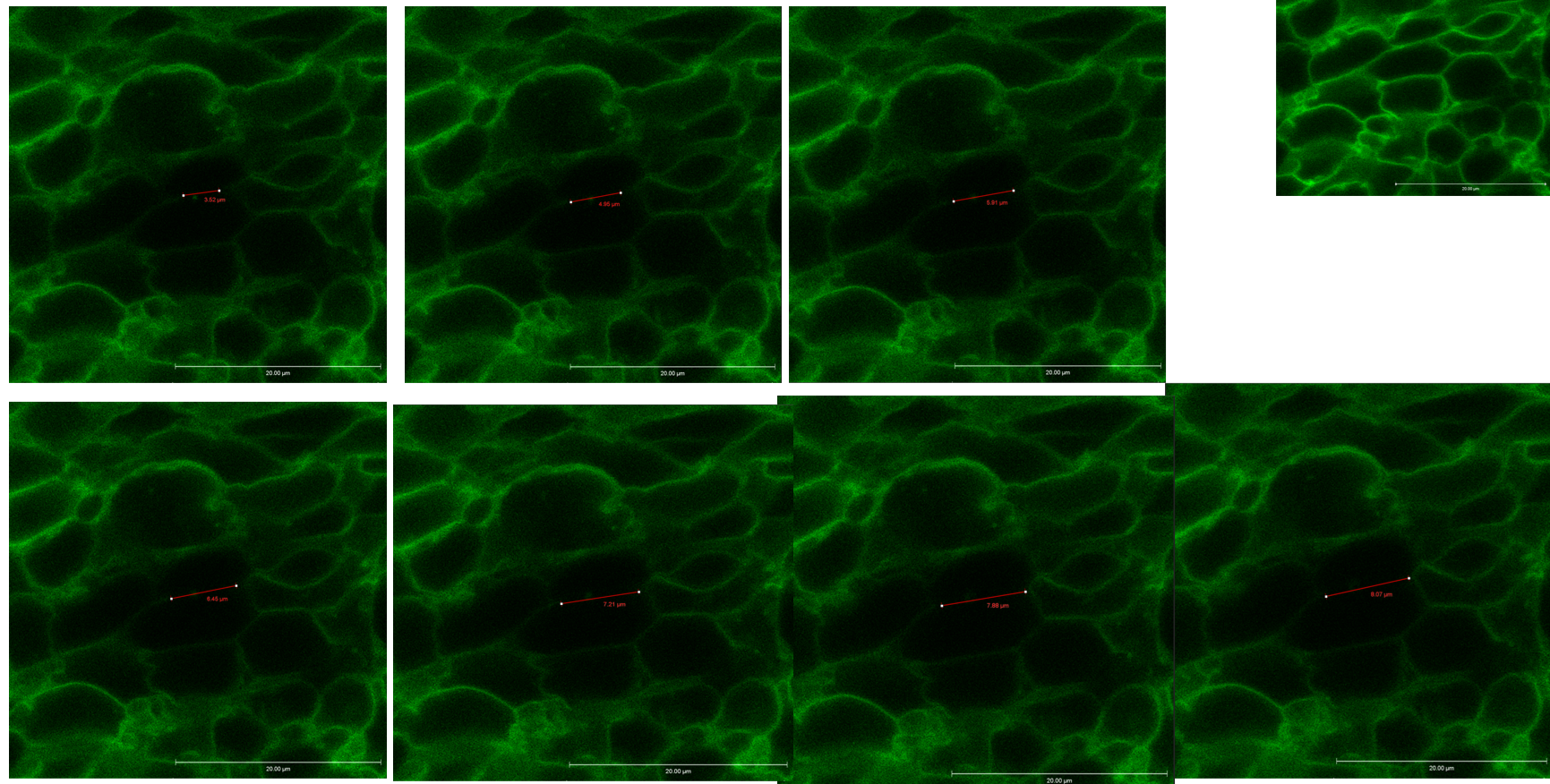

Supplement: Supplementary file 3 — Movie EV1 [file 44318_2026_771_MOESM3_ESM.zip › Fig 1 revised/Fig 1F bud pro n2.tif]

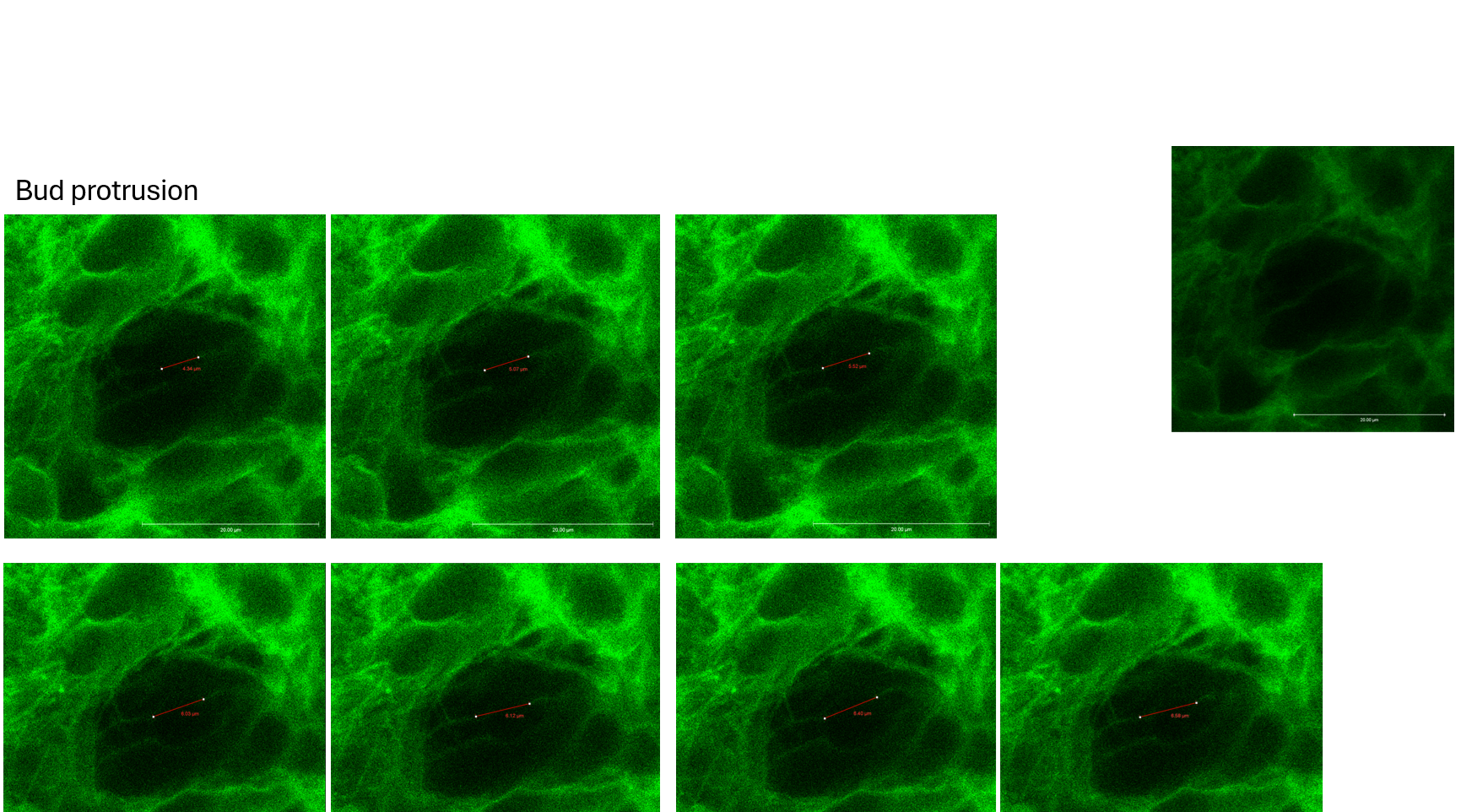

Supplement: Supplementary file 3 — Movie EV1 [file 44318_2026_771_MOESM3_ESM.zip › Fig 1 revised/Fig 1F bud pro n3.tif]

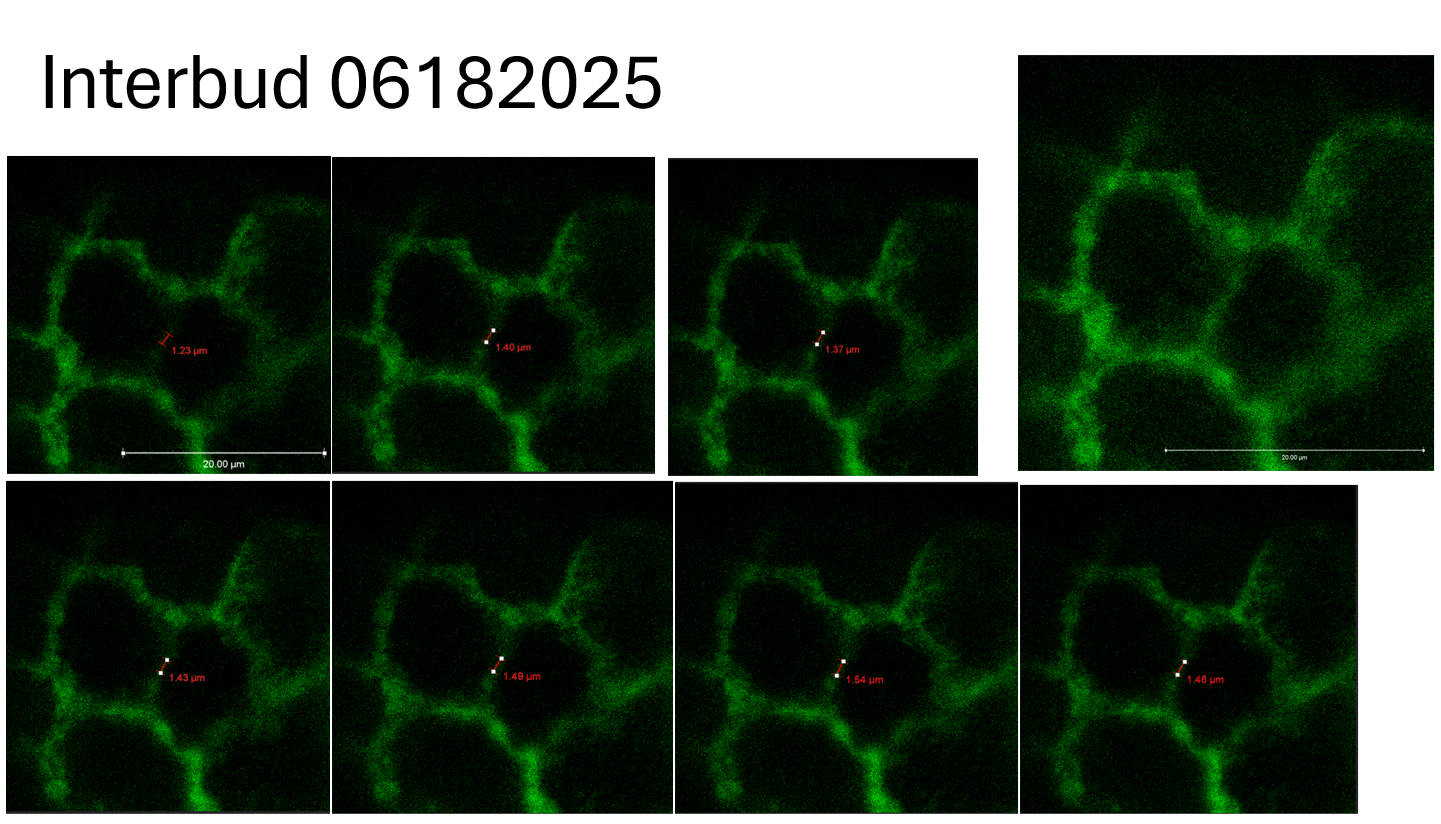

Supplement: Supplementary file 3 — Movie EV1 [file 44318_2026_771_MOESM3_ESM.zip › Fig 1 revised/Fig 1F interbud n1.tif]

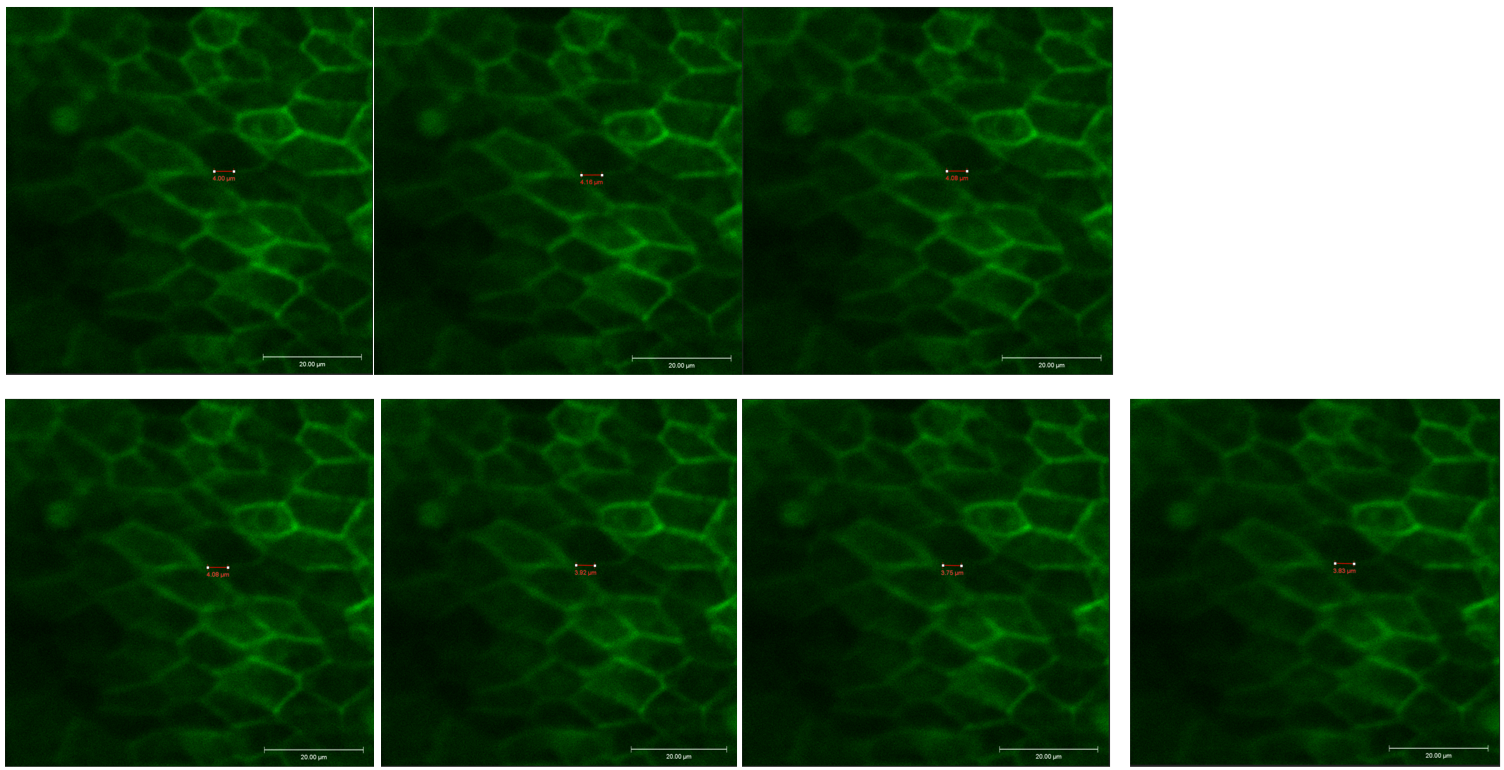

Supplement: Supplementary file 3 — Movie EV1 [file 44318_2026_771_MOESM3_ESM.zip › Fig 1 revised/Fig 1F interbud n2.tif]

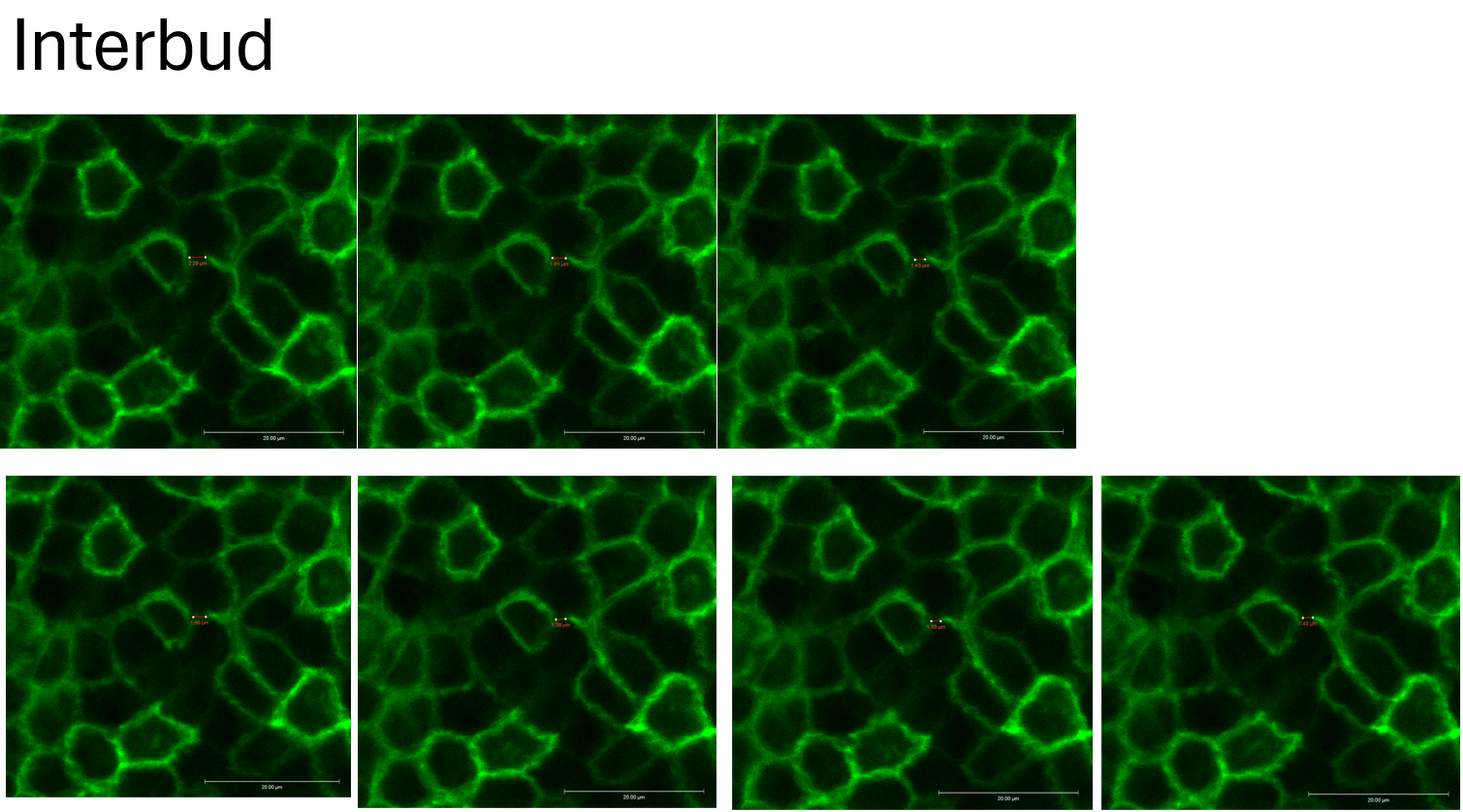

Supplement: Supplementary file 3 — Movie EV1 [file 44318_2026_771_MOESM3_ESM.zip › Fig 1 revised/Fig 1F interbud n3.tif]

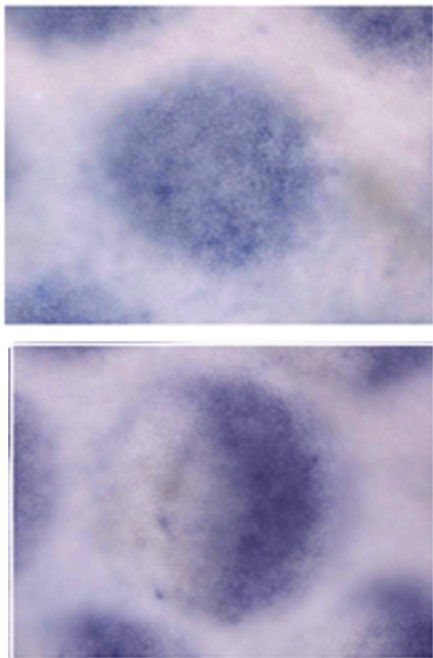

Supplement: Supplementary file 3 — Movie EV1 [file 44318_2026_771_MOESM3_ESM.zip › Fig 1 revised/Fig 1G snai2 n1.tif]

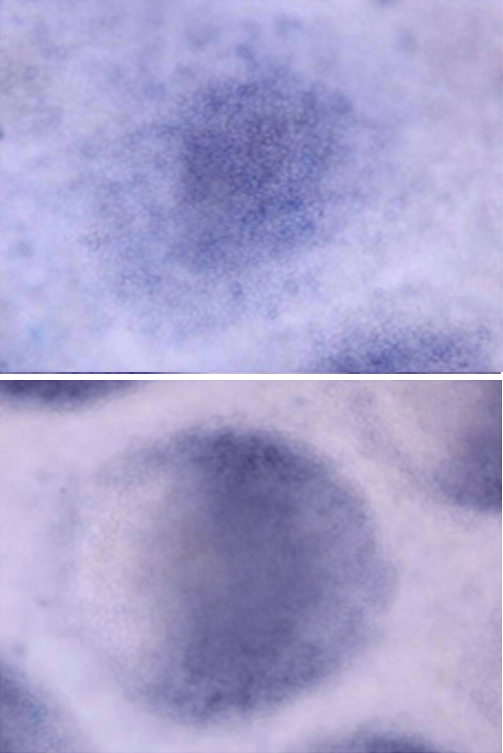

Supplement: Supplementary file 3 — Movie EV1 [file 44318_2026_771_MOESM3_ESM.zip › Fig 1 revised/Fig 1G snai2 n2.tif]

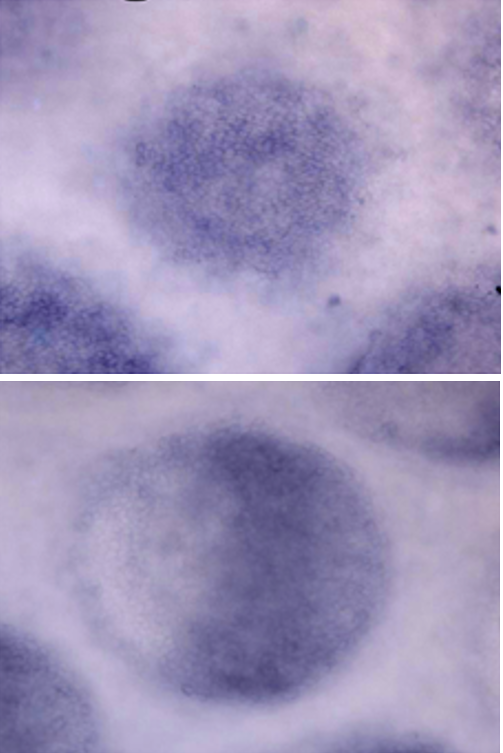

Supplement: Supplementary file 3 — Movie EV1 [file 44318_2026_771_MOESM3_ESM.zip › Fig 1 revised/Fig 1G snai2 n3.tif]

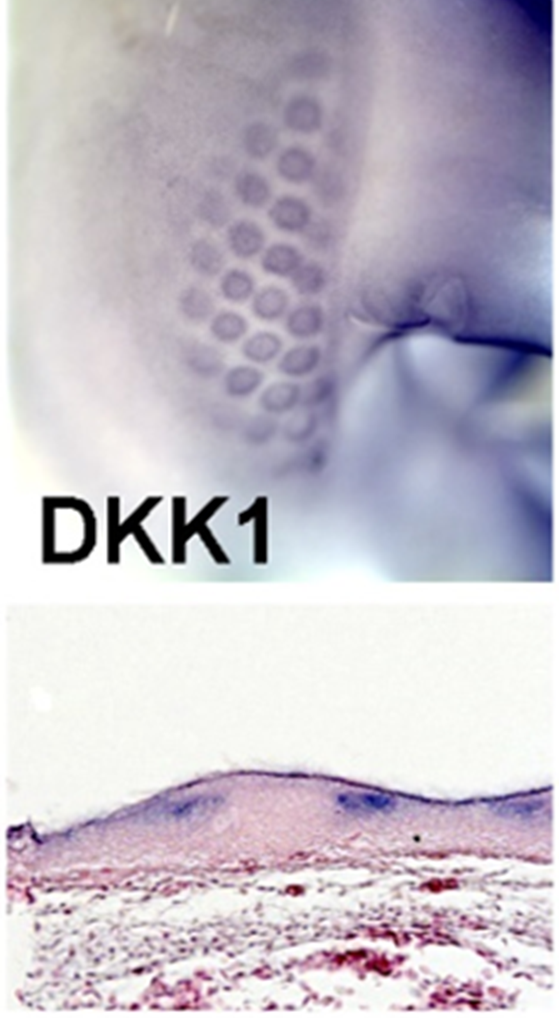

Supplement: Supplementary file 3 — Movie EV1 [file 44318_2026_771_MOESM3_ESM.zip › Fig 1 revised/Fig 1H Dkk n1.tif]

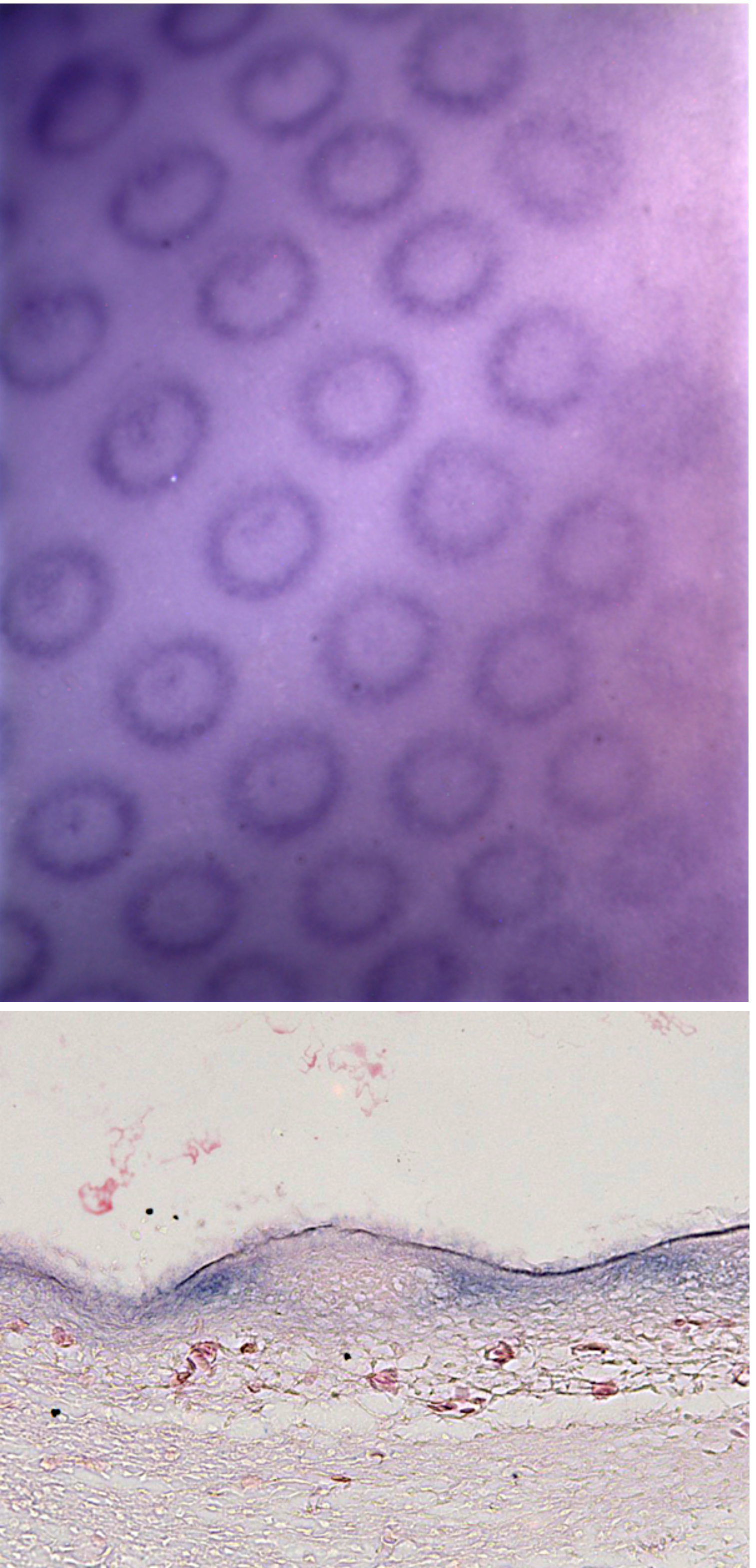

Supplement: Supplementary file 3 — Movie EV1 [file 44318_2026_771_MOESM3_ESM.zip › Fig 1 revised/Fig 1H Dkk n2.tiff]

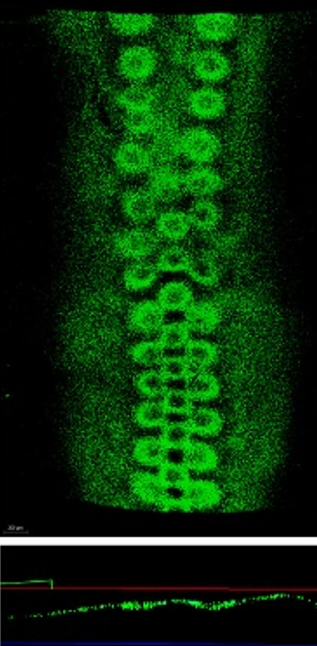

Supplement: Supplementary file 3 — Movie EV1 [file 44318_2026_771_MOESM3_ESM.zip › Fig 1 revised/Fig 1H Dkk n3.tif]

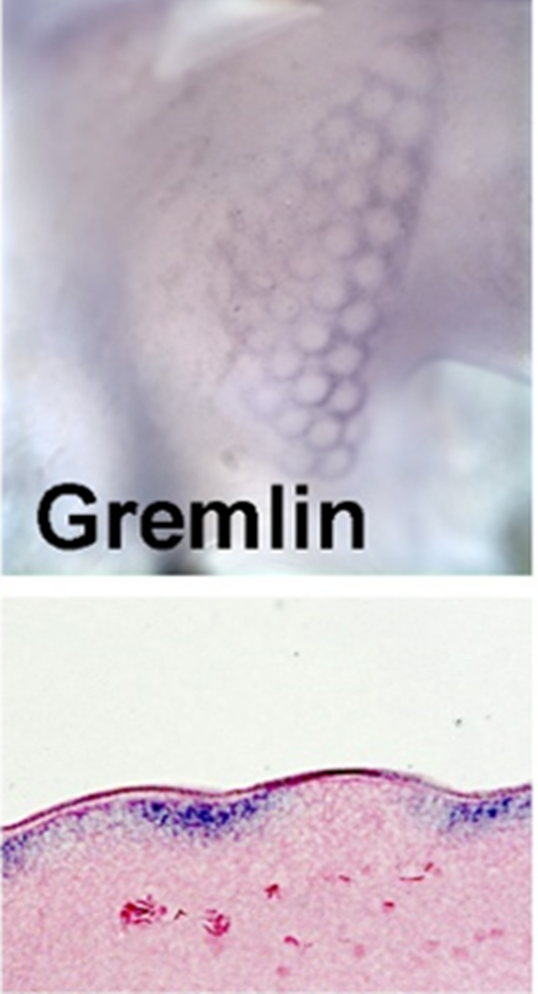

Supplement: Supplementary file 3 — Movie EV1 [file 44318_2026_771_MOESM3_ESM.zip › Fig 1 revised/Fig 1H Gremlin n1.tif]

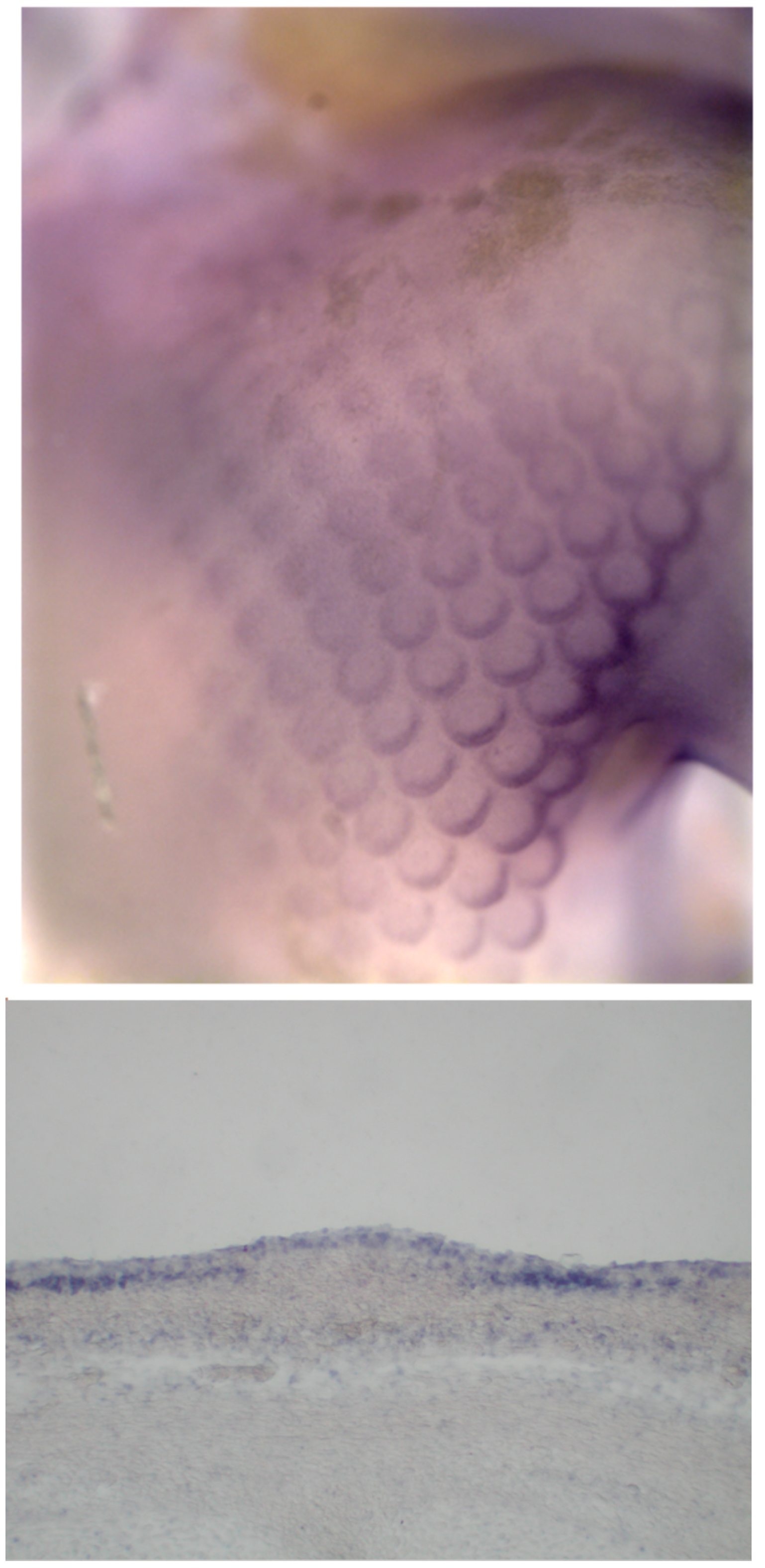

Supplement: Supplementary file 3 — Movie EV1 [file 44318_2026_771_MOESM3_ESM.zip › Fig 1 revised/Fig 1H Gremlin n2.tiff]

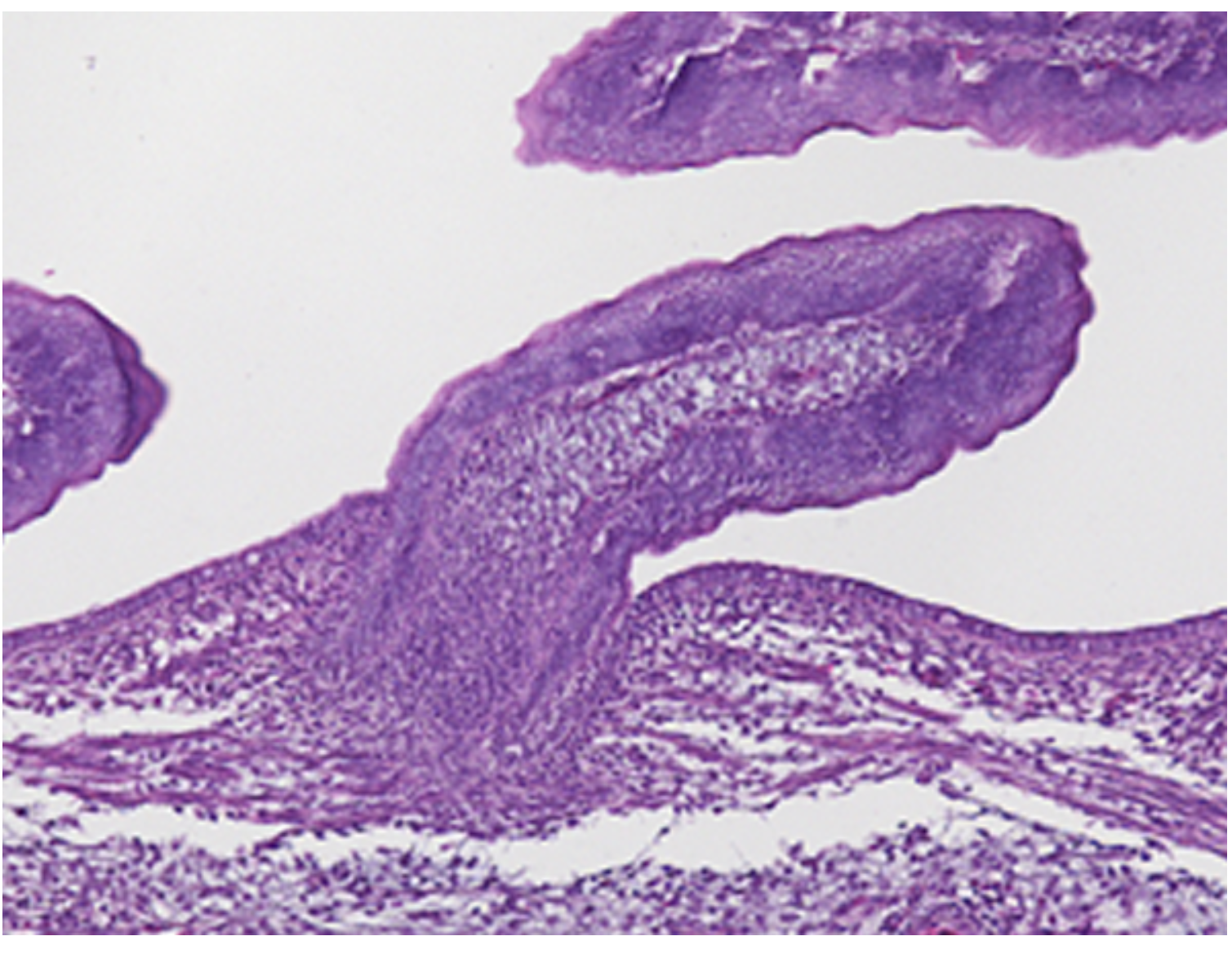

Supplement: Supplementary file 3 — Movie EV1 [file 44318_2026_771_MOESM3_ESM.zip › Fig 1 revised/Fig 1J E11 control n1.tiff]

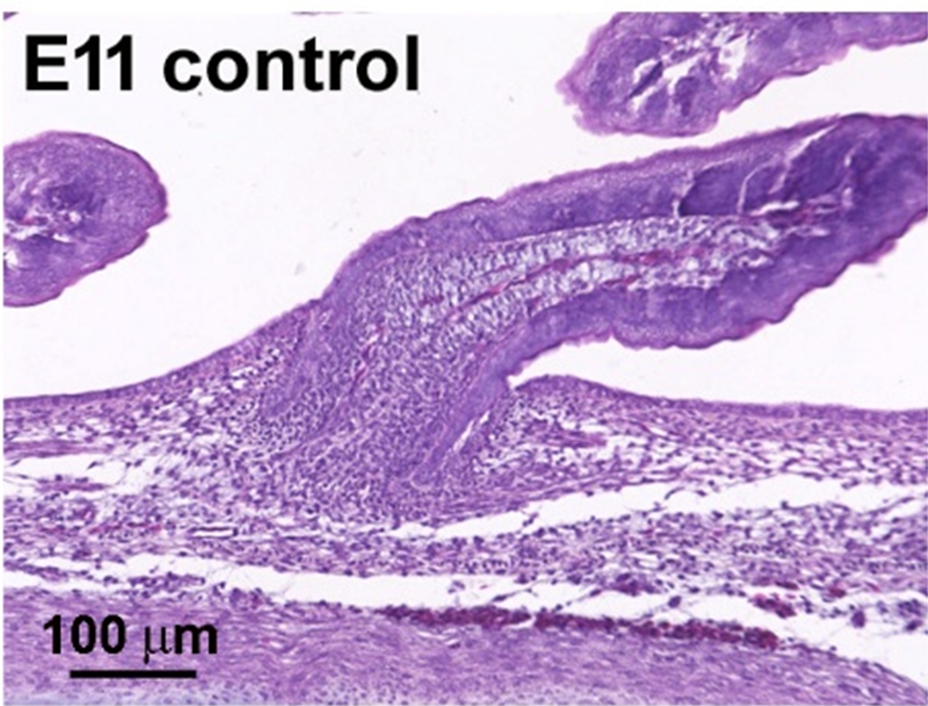

Supplement: Supplementary file 3 — Movie EV1 [file 44318_2026_771_MOESM3_ESM.zip › Fig 1 revised/Fig 1J E11 control n2.tiff]

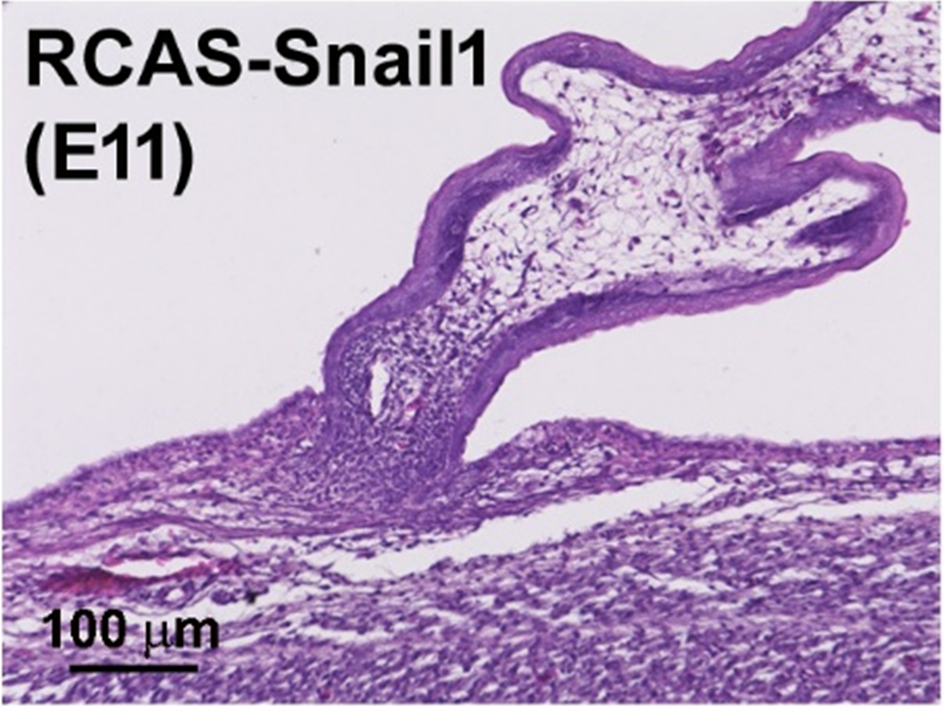

Supplement: Supplementary file 3 — Movie EV1 [file 44318_2026_771_MOESM3_ESM.zip › Fig 1 revised/Fig 1J E11 Snai1 n1.tif]

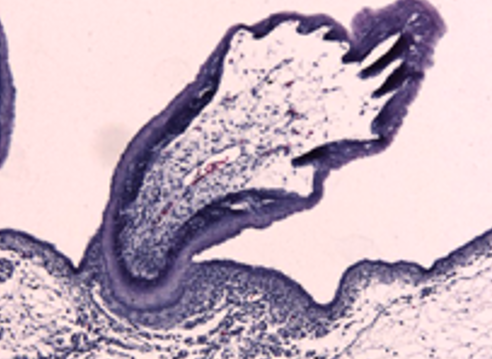

Supplement: Supplementary file 3 — Movie EV1 [file 44318_2026_771_MOESM3_ESM.zip › Fig 1 revised/Fig 1J E11 Snai1 n2.tiff]

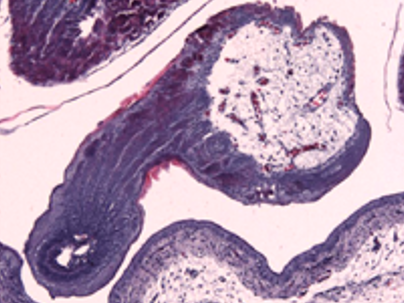

Supplement: Supplementary file 3 — Movie EV1 [file 44318_2026_771_MOESM3_ESM.zip › Fig 1 revised/Fig 1J E11 Snai1 n3.tif]

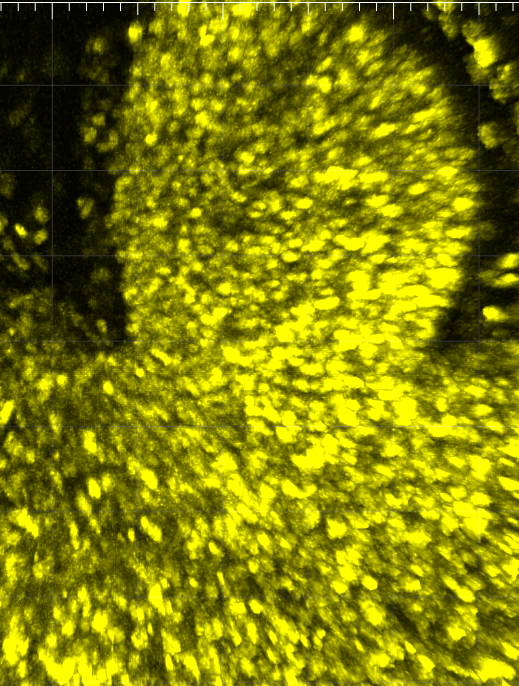

Supplement: Supplementary file 3 — Movie EV1 [file 44318_2026_771_MOESM3_ESM.zip › Fig 1 revised/Fig 1K Snai1 tracking n1.tif]

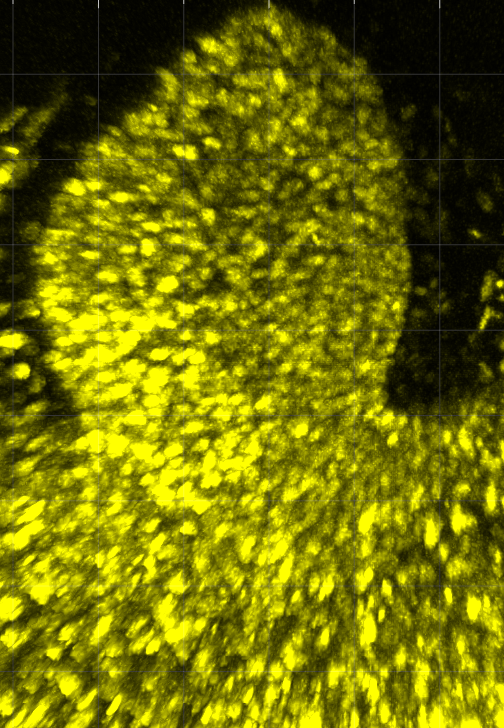

Supplement: Supplementary file 3 — Movie EV1 [file 44318_2026_771_MOESM3_ESM.zip › Fig 1 revised/Fig 1K Snai1 tracking n2.tif]

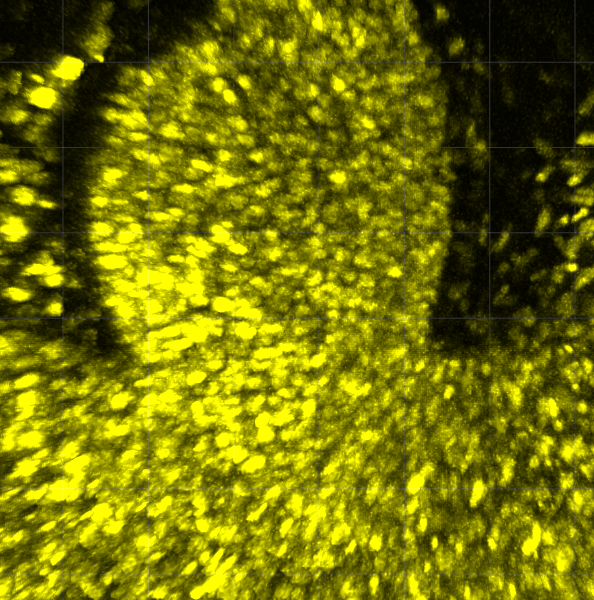

Supplement: Supplementary file 3 — Movie EV1 [file 44318_2026_771_MOESM3_ESM.zip › Fig 1 revised/Fig 1K Snai1 tracking n3.tif]

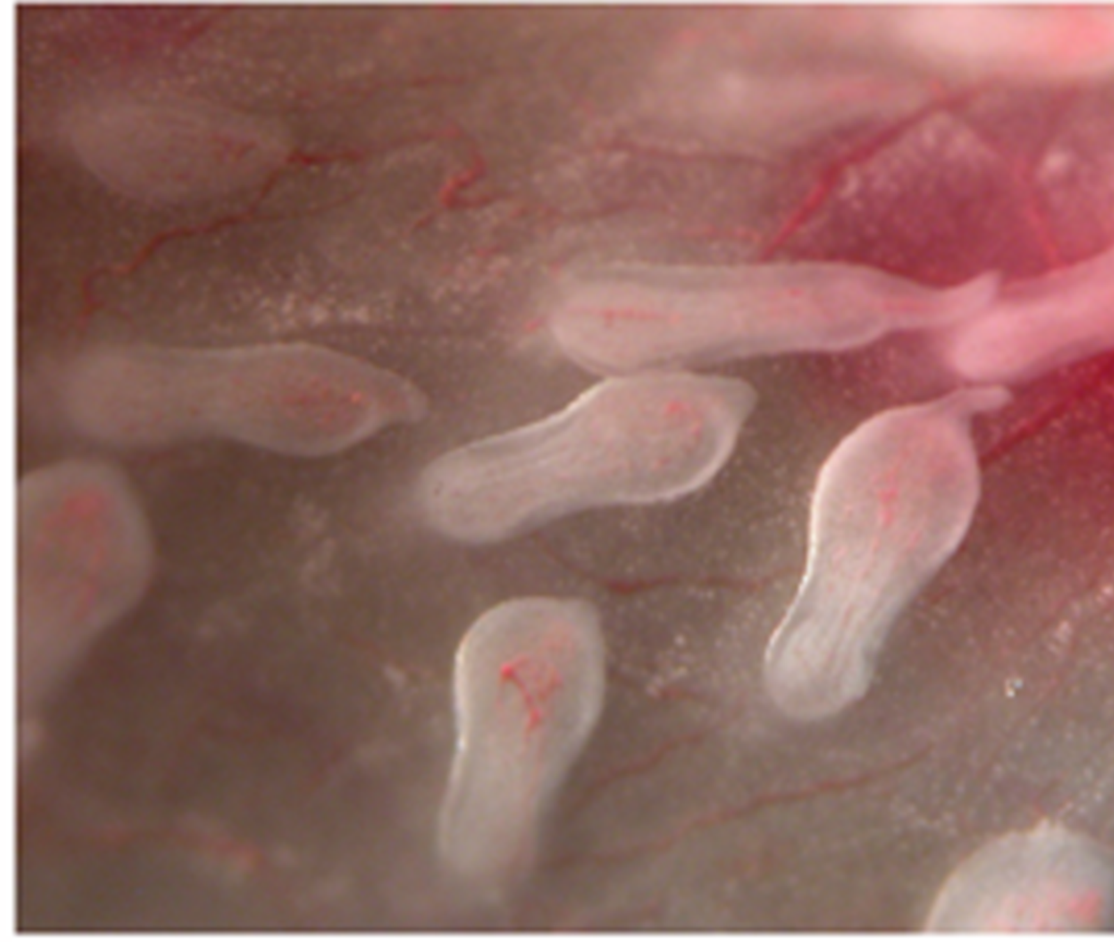

Supplement: Supplementary file 3 — Movie EV1 [file 44318_2026_771_MOESM3_ESM.zip › Fig 1 revised/Fig 1M Sprty phenotype n1.tif]

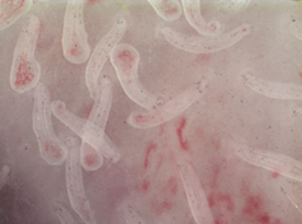

Supplement: Supplementary file 3 — Movie EV1 [file 44318_2026_771_MOESM3_ESM.zip › Fig 1 revised/Fig 1M Sprty phenotype n2.tif]

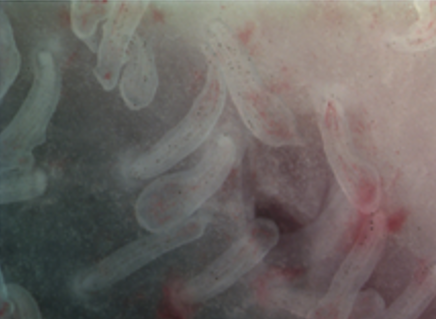

Supplement: Supplementary file 3 — Movie EV1 [file 44318_2026_771_MOESM3_ESM.zip › Fig 1 revised/Fig 1M Sprty phenotype n3.tif]

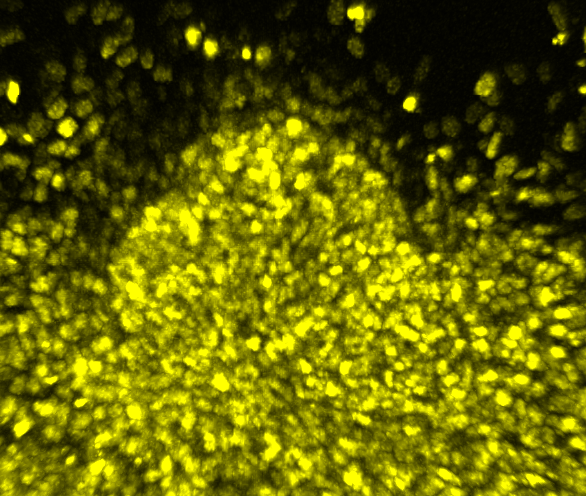

Supplement: Supplementary file 4 — Movie EV2 [file 44318_2026_771_MOESM4_ESM.zip › Fig 2/Fig 2N Wnt3a tracking n3.tif]

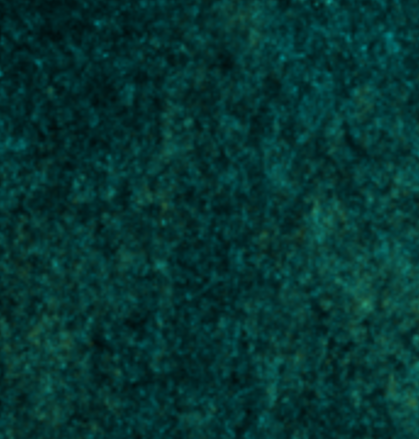

Supplement: Supplementary file 4 — Movie EV2 [file 44318_2026_771_MOESM4_ESM.zip › Fig 2/apteric n1.tif]

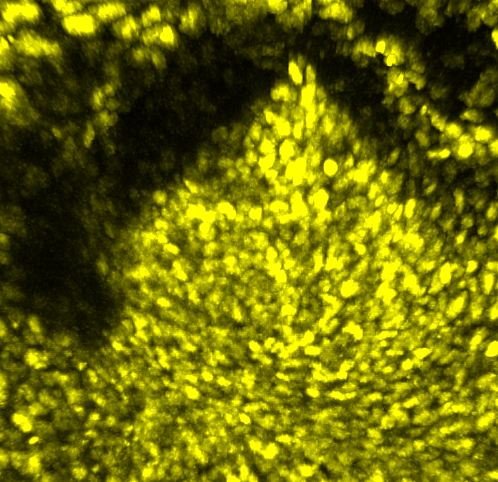

Supplement: Supplementary file 4 — Movie EV2 [file 44318_2026_771_MOESM4_ESM.zip › Fig 2/Fig 2N Wnt3a tracking n2.tif]

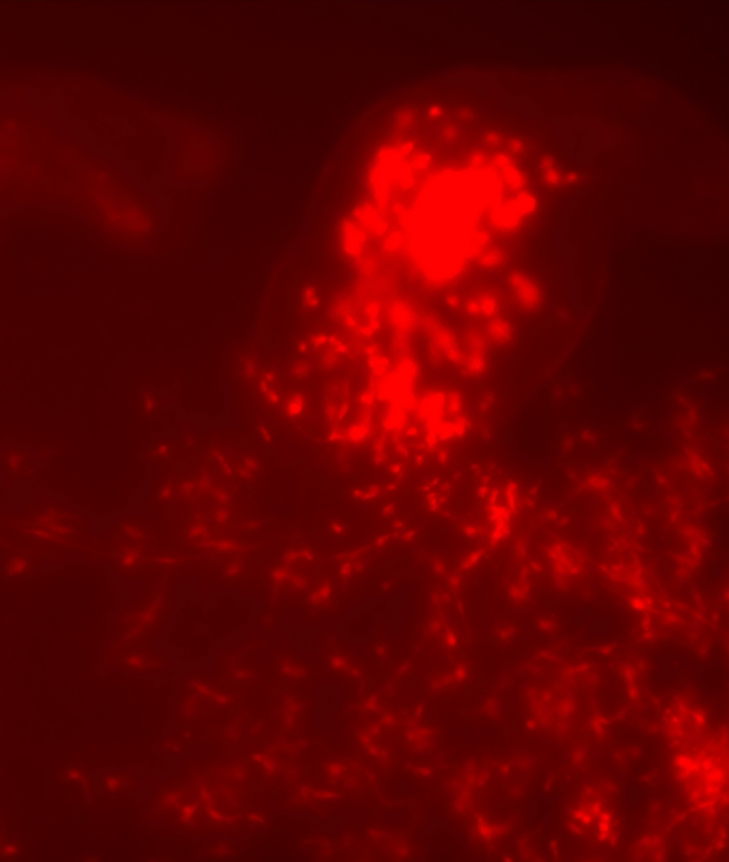

Supplement: Supplementary file 4 — Movie EV2 [file 44318_2026_771_MOESM4_ESM.zip › Fig 2/Fig 2A inv video img n1.tif]

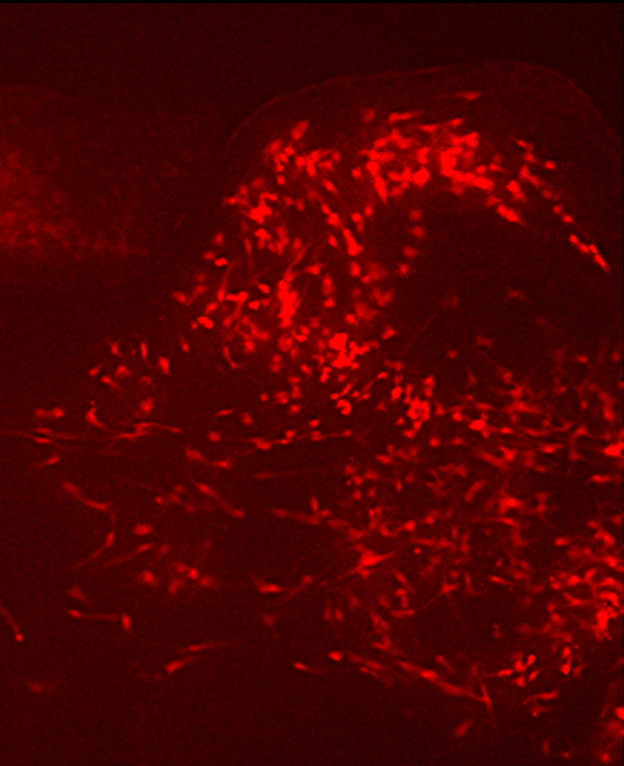

Supplement: Supplementary file 4 — Movie EV2 [file 44318_2026_771_MOESM4_ESM.zip › Fig 2/Fig 2A inv video img n3.tif]

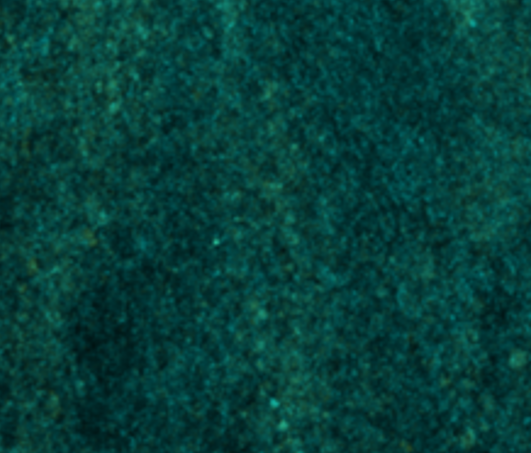

Supplement: Supplementary file 4 — Movie EV2 [file 44318_2026_771_MOESM4_ESM.zip › Fig 2/apteric n3.tif]

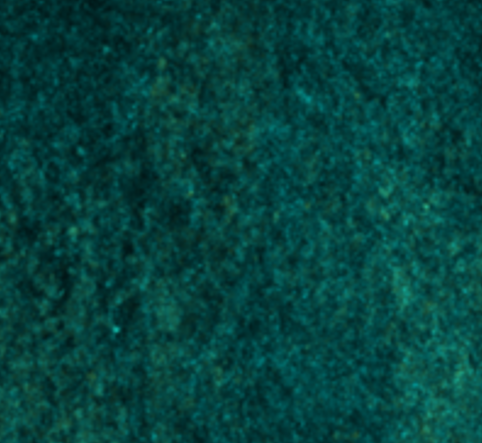

Supplement: Supplementary file 4 — Movie EV2 [file 44318_2026_771_MOESM4_ESM.zip › Fig 2/apteric n2.tif]

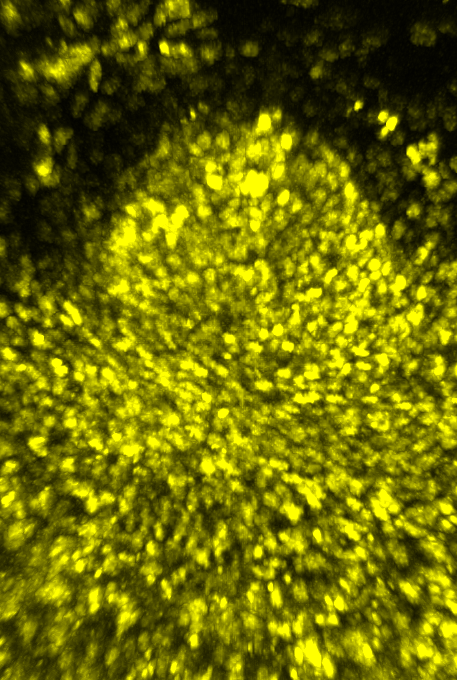

Supplement: Supplementary file 4 — Movie EV2 [file 44318_2026_771_MOESM4_ESM.zip › Fig 2/Fig 2N Wnt3a tracking n1.tif]

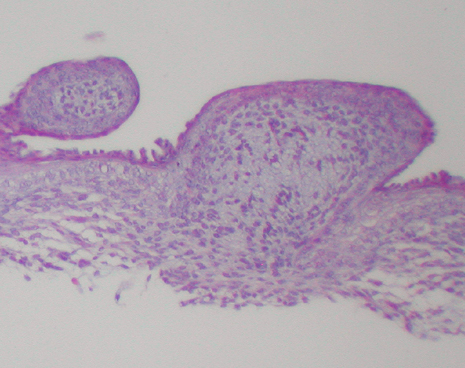

Supplement: Supplementary file 4 — Movie EV2 [file 44318_2026_771_MOESM4_ESM.zip › Fig 2/Fig 2M Wnt3a n2.tif]

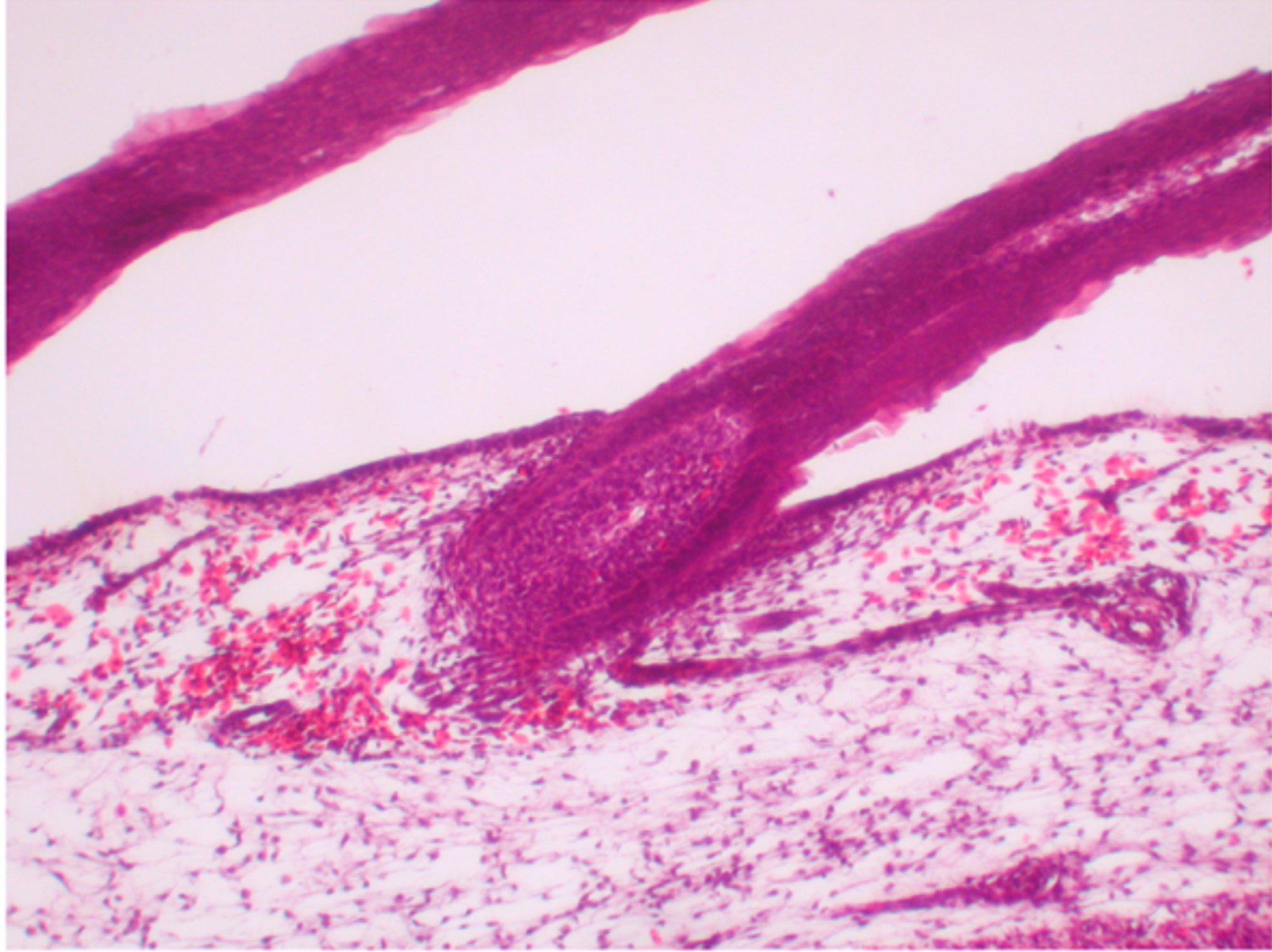

Supplement: Supplementary file 4 — Movie EV2 [file 44318_2026_771_MOESM4_ESM.zip › Fig 2/Fig 2M ctl n2.tiff]

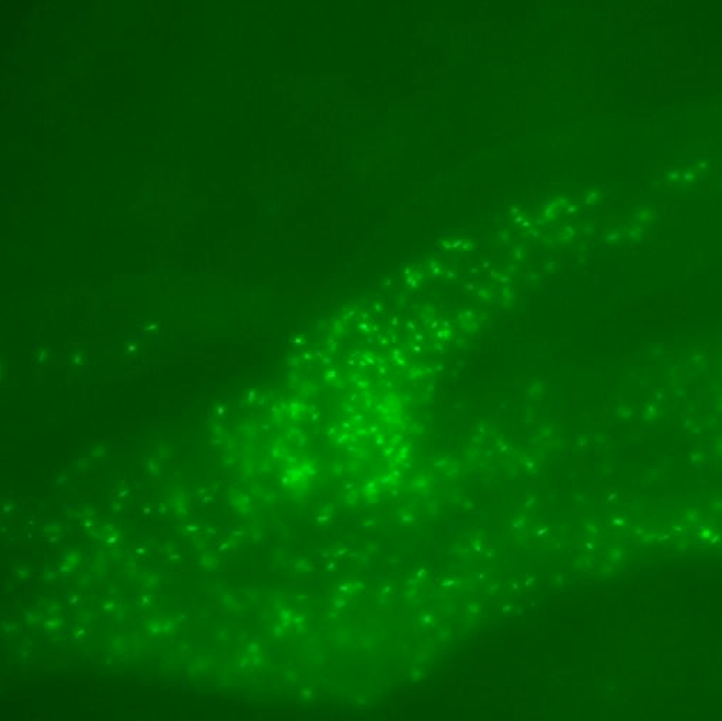

Supplement: Supplementary file 4 — Movie EV2 [file 44318_2026_771_MOESM4_ESM.zip › Fig 2/Fig 2A inv video img n2.tif]

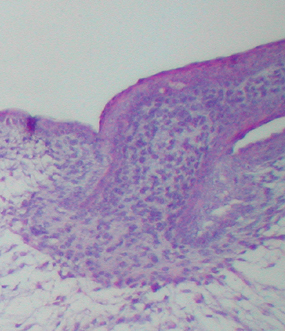

Supplement: Supplementary file 4 — Movie EV2 [file 44318_2026_771_MOESM4_ESM.zip › Fig 2/Fig 2M Ctl n2.tif]

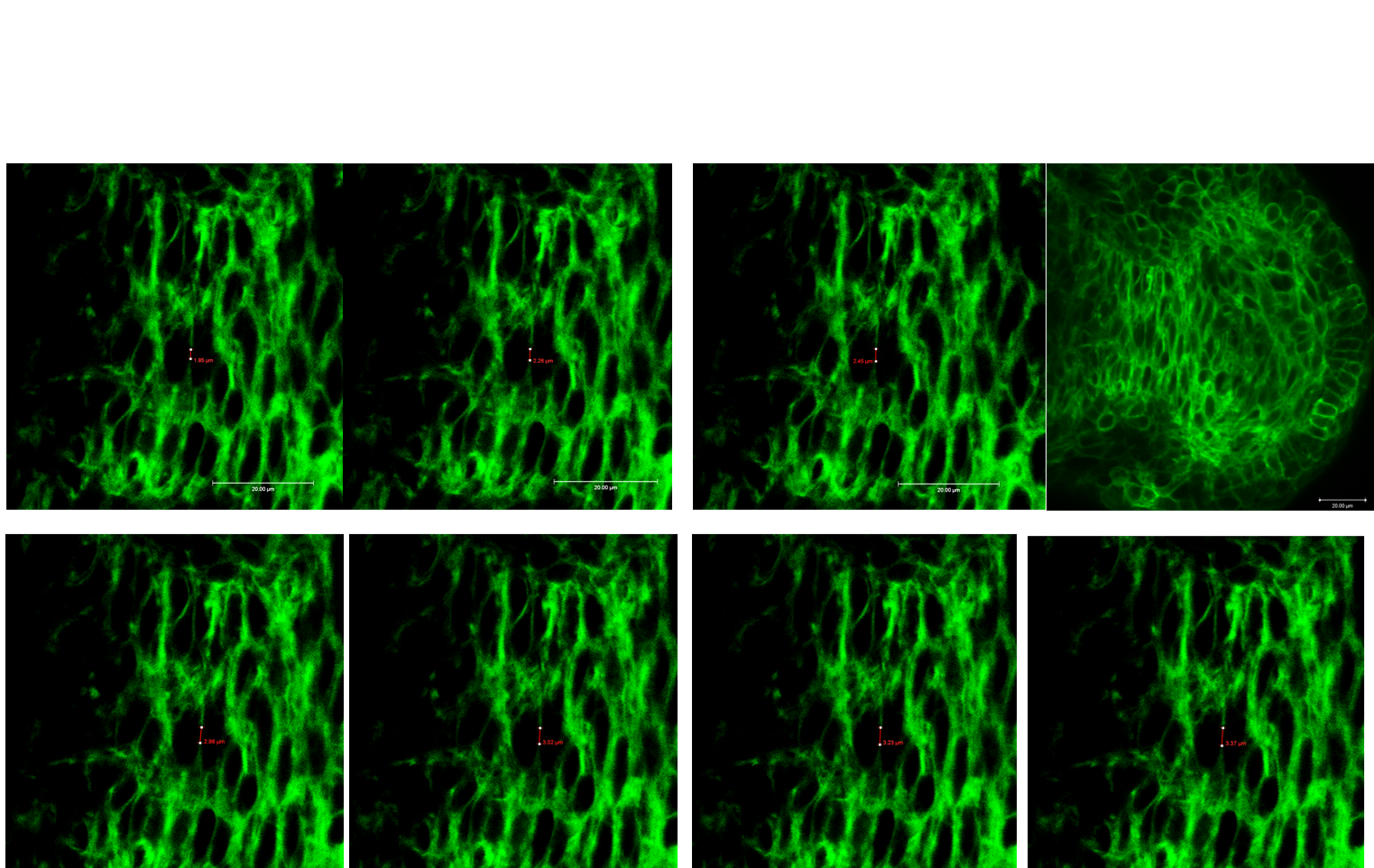

Supplement: Supplementary file 4 — Movie EV2 [file 44318_2026_771_MOESM4_ESM.zip › Fig 2/Fig 2H neck n1.tif]

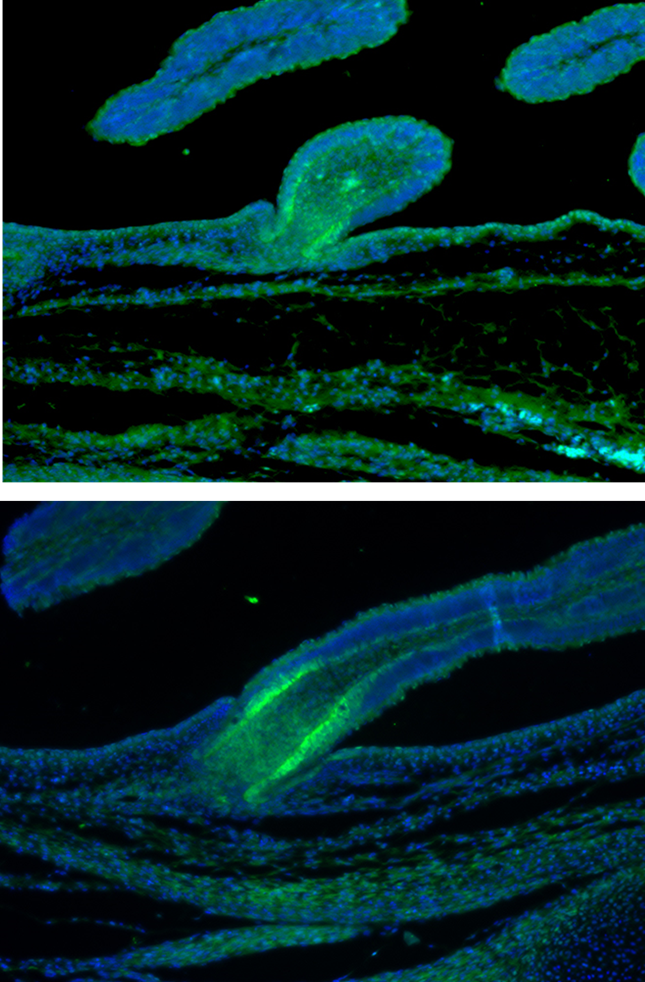

Supplement: Supplementary file 4 — Movie EV2 [file 44318_2026_771_MOESM4_ESM.zip › Fig 2/Fig 2I YAP n1.tif]

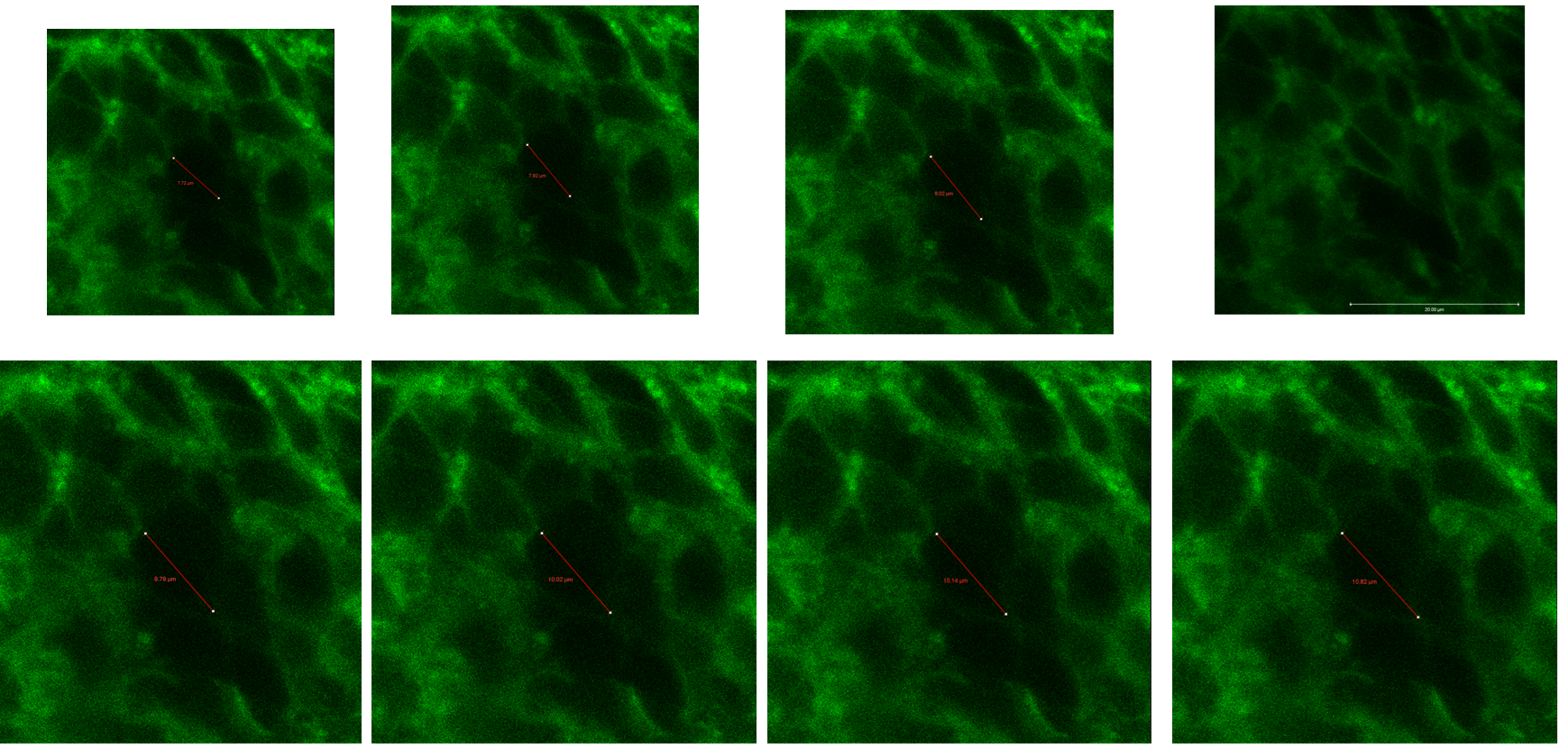

Supplement: Supplementary file 4 — Movie EV2 [file 44318_2026_771_MOESM4_ESM.zip › Fig 2/Fig 2H neck n3.tif]

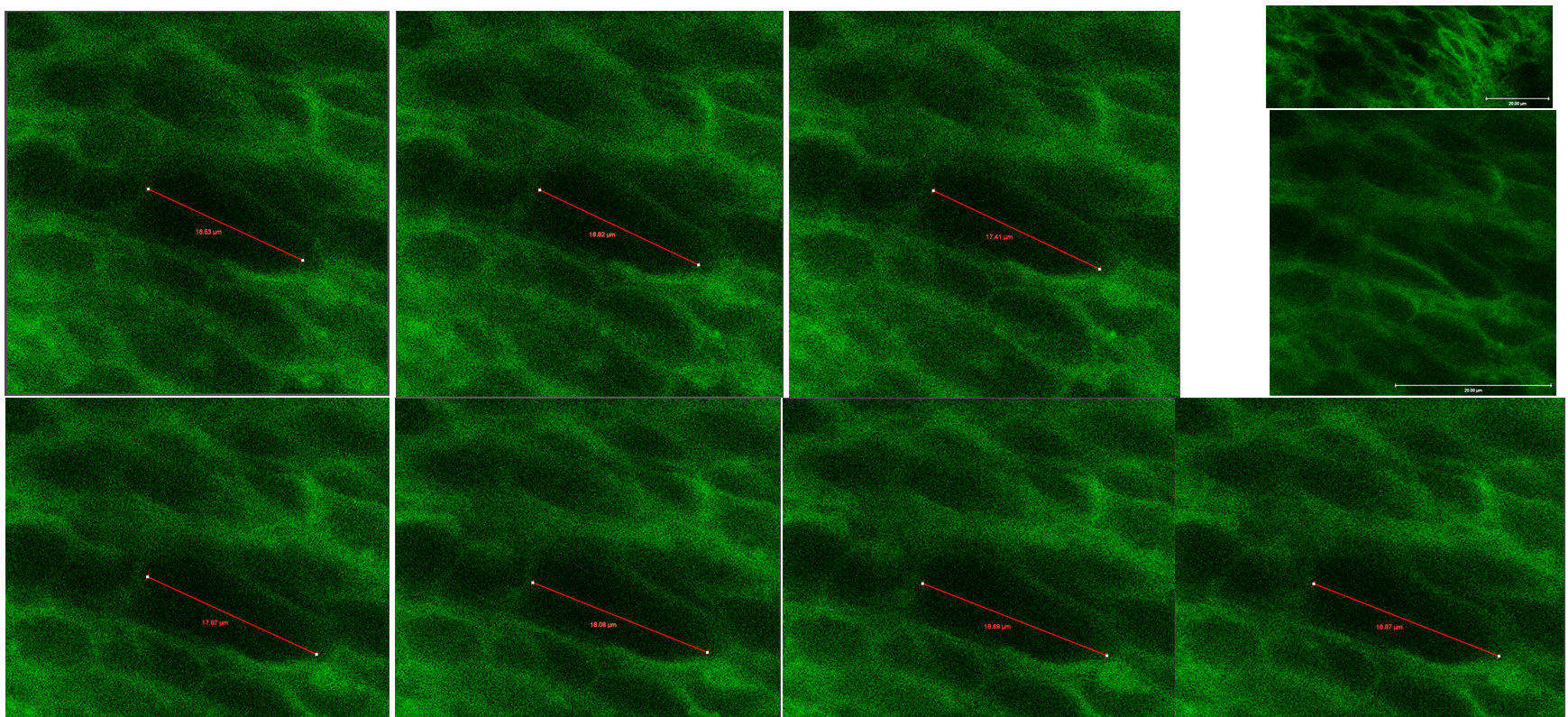

Supplement: Supplementary file 4 — Movie EV2 [file 44318_2026_771_MOESM4_ESM.zip › Fig 2/Fig 2H neck n2.tif]

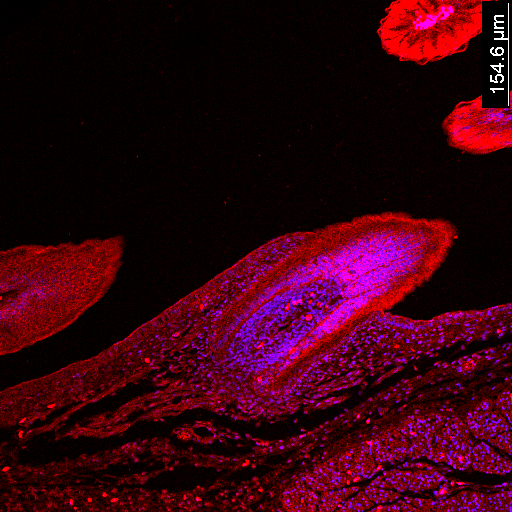

Supplement: Supplementary file 4 — Movie EV2 [file 44318_2026_771_MOESM4_ESM.zip › Fig 2/Fig 2K Ctl MMP n1.tif]

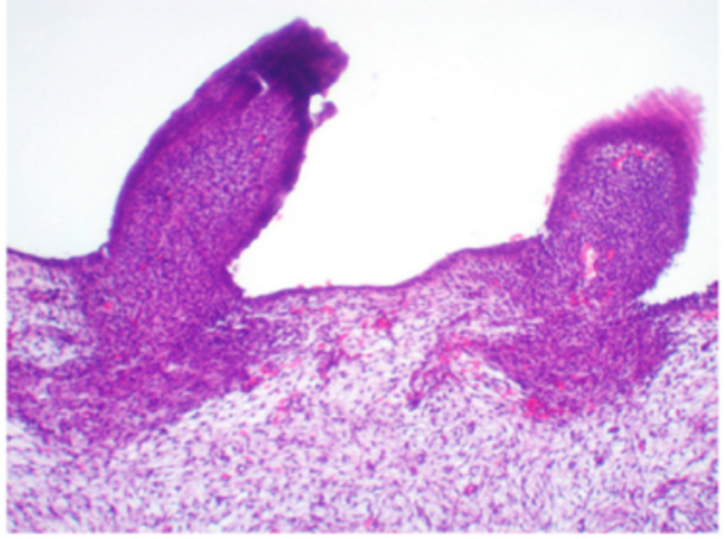

Supplement: Supplementary file 4 — Movie EV2 [file 44318_2026_771_MOESM4_ESM.zip › Fig 2/Fig 2M Wnt3a n1.tiff]

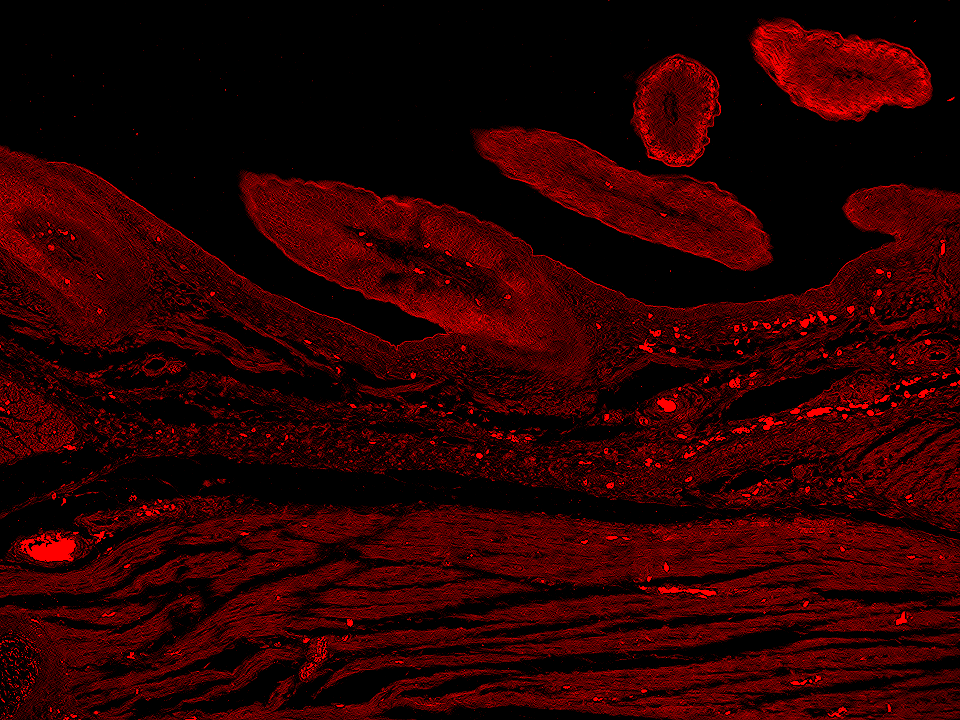

Supplement: Supplementary file 4 — Movie EV2 [file 44318_2026_771_MOESM4_ESM.zip › Fig 2/Fig 2K Ctl MMP n2.tif]

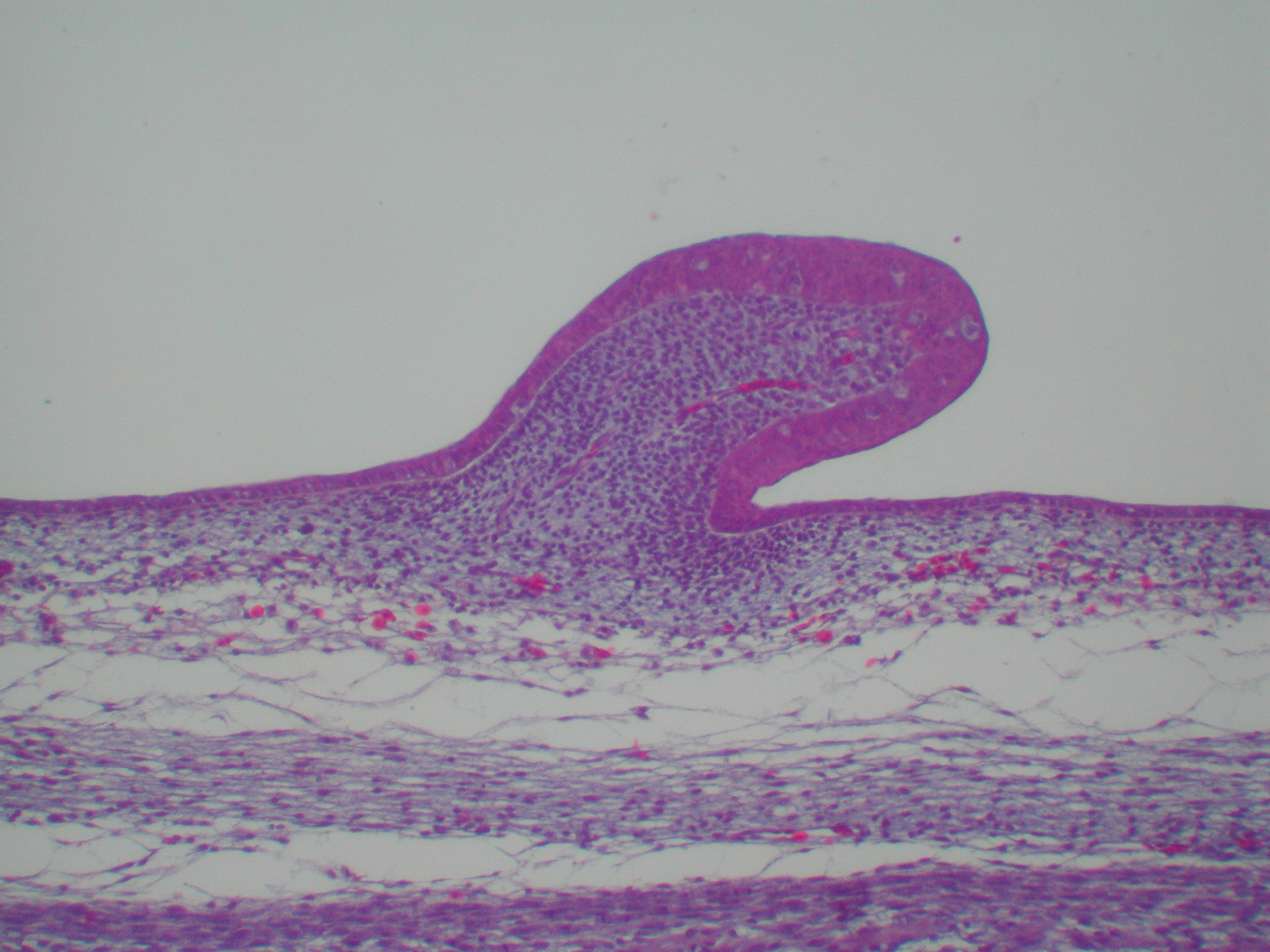

Supplement: Supplementary file 4 — Movie EV2 [file 44318_2026_771_MOESM4_ESM.zip › Fig 2/Fig 2A inv E9 n1.tif]

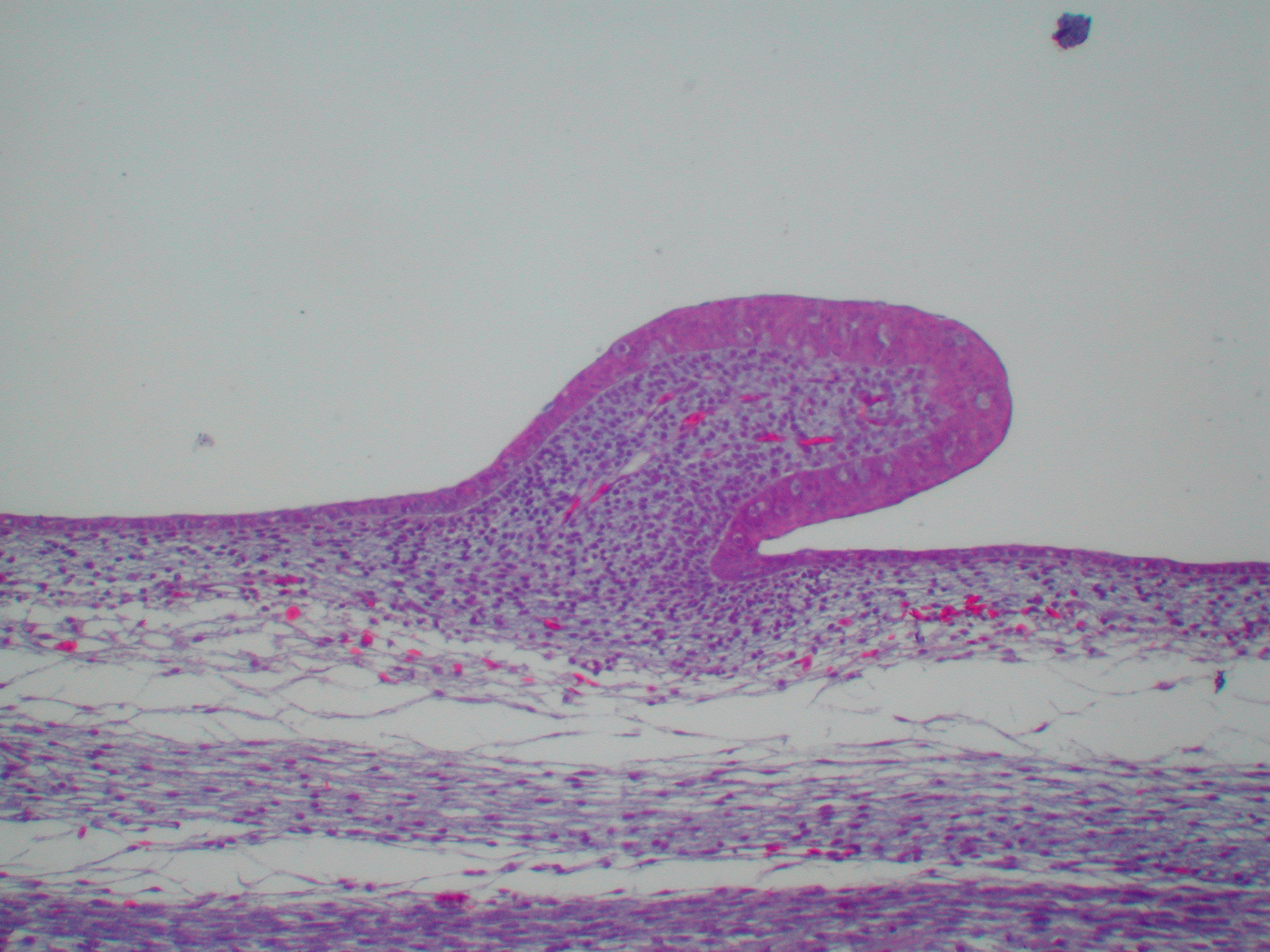

Supplement: Supplementary file 4 — Movie EV2 [file 44318_2026_771_MOESM4_ESM.zip › Fig 2/Fig 2A inv E9 n2.tif]

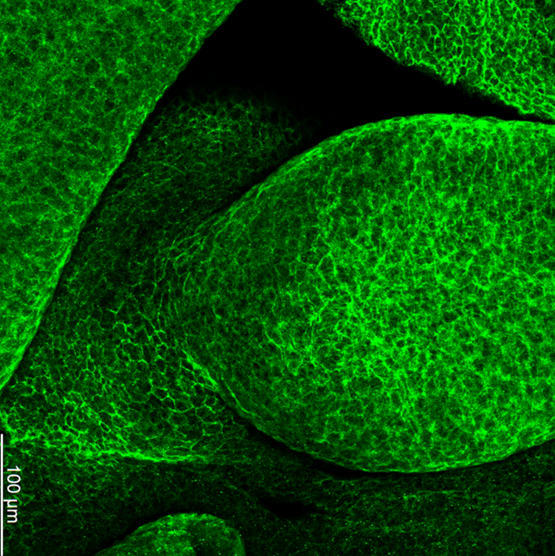

Supplement: Supplementary file 4 — Movie EV2 [file 44318_2026_771_MOESM4_ESM.zip › Fig 2/Fig 2E neck n2.tif]

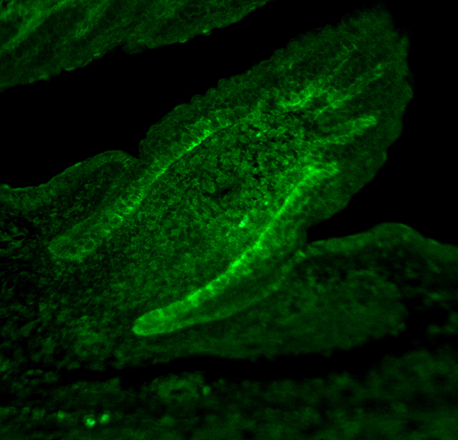

Supplement: Supplementary file 4 — Movie EV2 [file 44318_2026_771_MOESM4_ESM.zip › Fig 2/Yap n-4.tif]

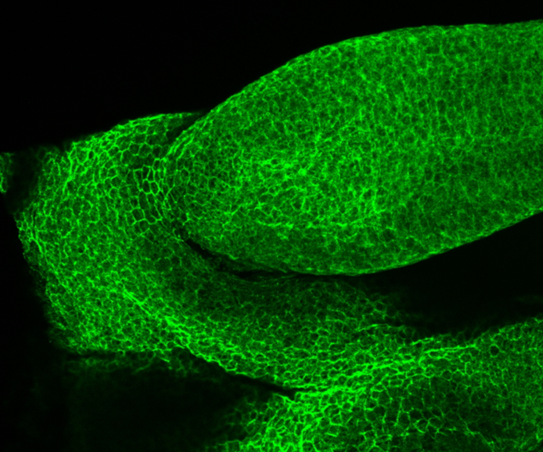

Supplement: Supplementary file 4 — Movie EV2 [file 44318_2026_771_MOESM4_ESM.zip › Fig 2/Fig 2E neck n3.tif]

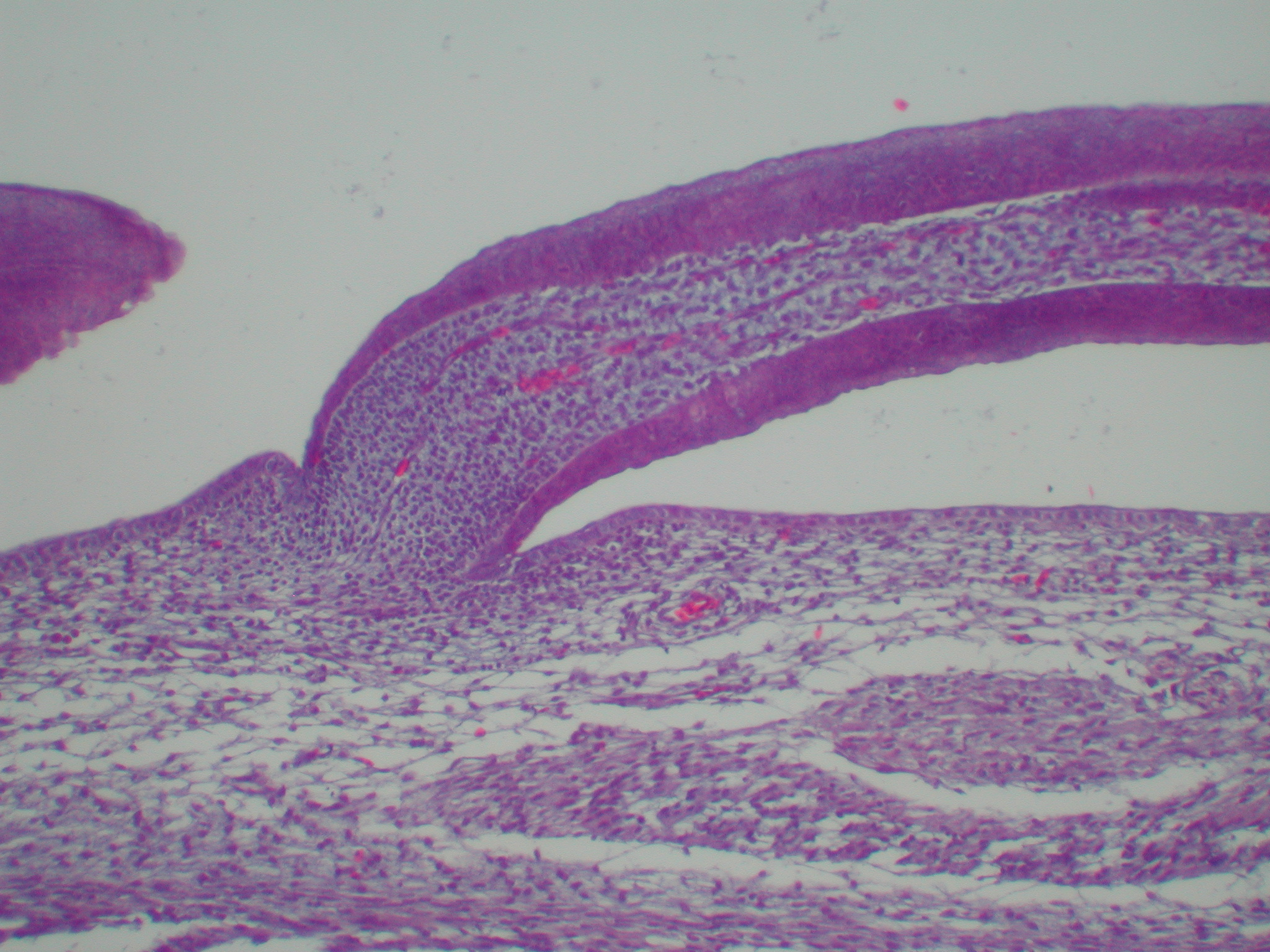

Supplement: Supplementary file 4 — Movie EV2 [file 44318_2026_771_MOESM4_ESM.zip › Fig 2/Fig 2A inv E10 n1.tif]

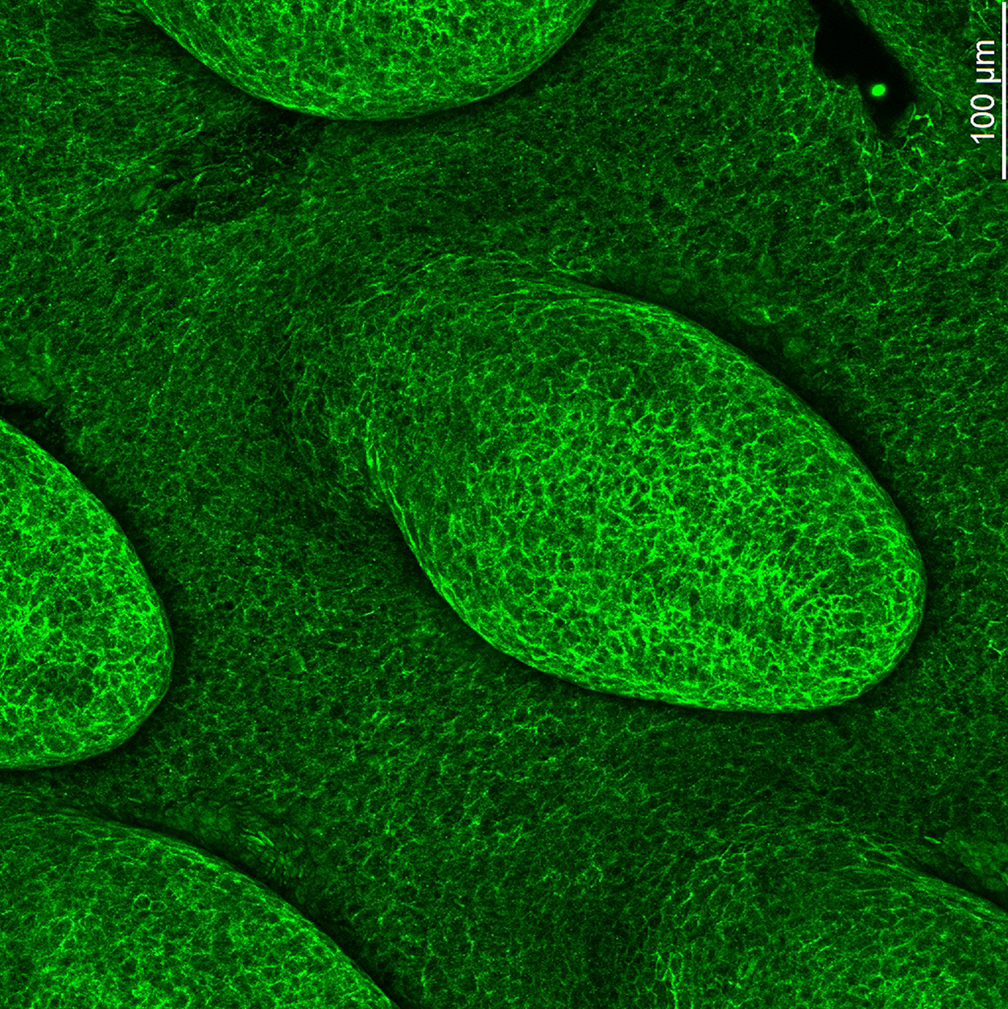

Supplement: Supplementary file 4 — Movie EV2 [file 44318_2026_771_MOESM4_ESM.zip › Fig 2/Fig 2E neck n1.tif]

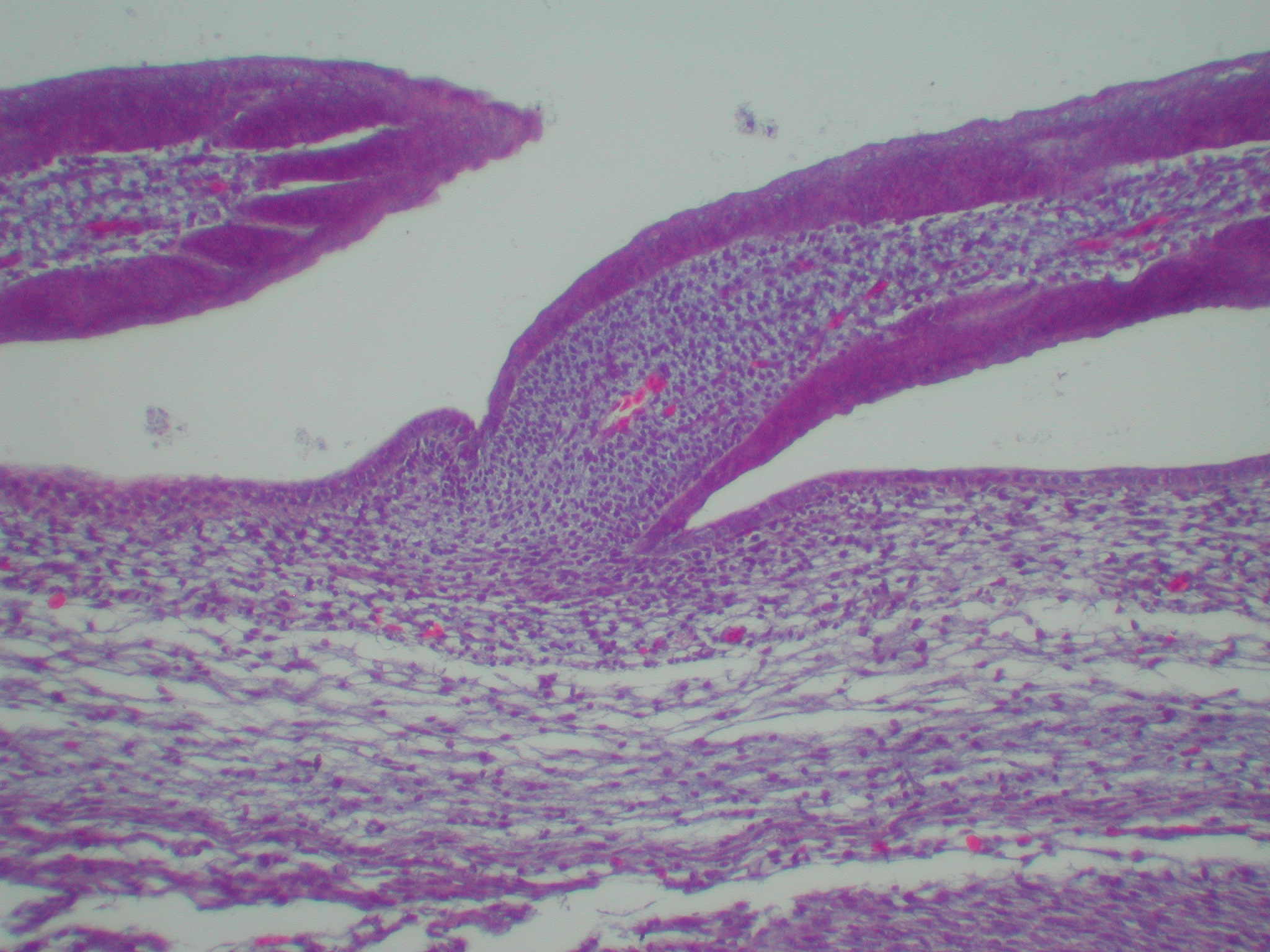

Supplement: Supplementary file 4 — Movie EV2 [file 44318_2026_771_MOESM4_ESM.zip › Fig 2/Fig 2A inv E10 n2.tif]

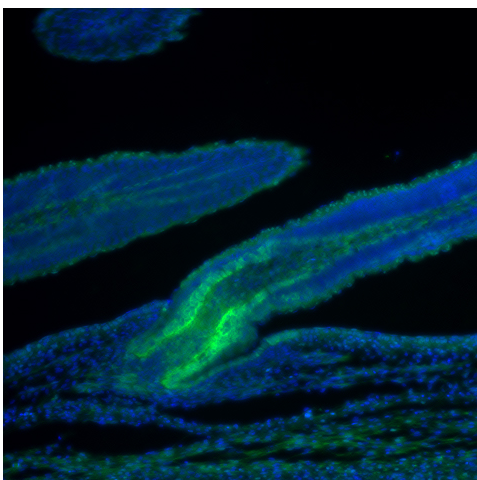

Supplement: Supplementary file 4 — Movie EV2 [file 44318_2026_771_MOESM4_ESM.zip › Fig 2/Yap n-2.tif]

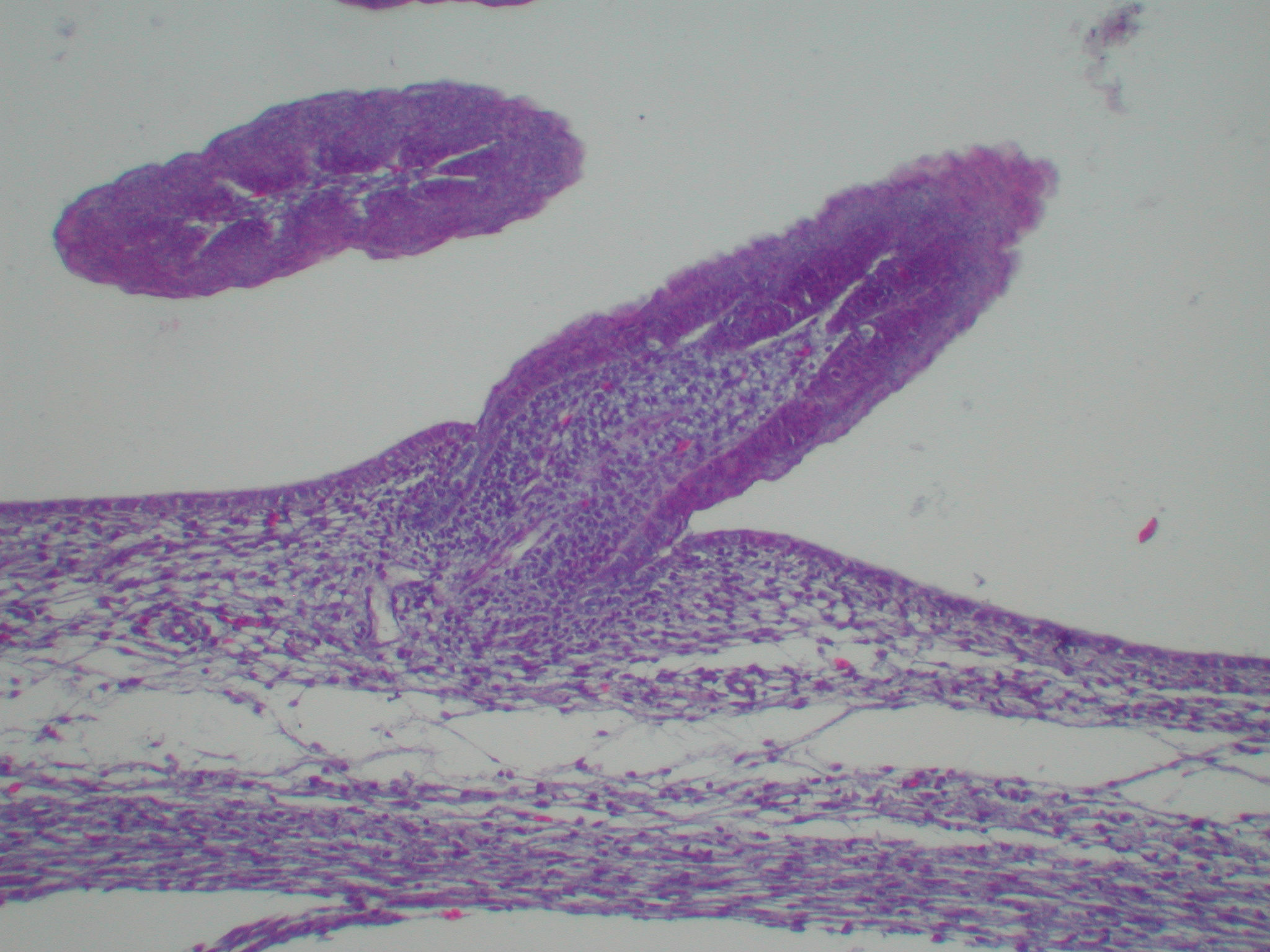

Supplement: Supplementary file 4 — Movie EV2 [file 44318_2026_771_MOESM4_ESM.zip › Fig 2/Fig 2A inv E12 n3.tif]

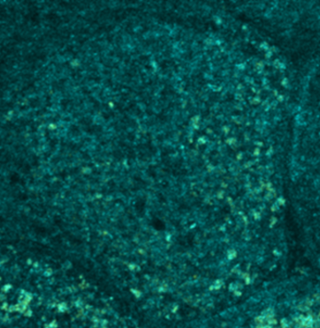

Supplement: Supplementary file 4 — Movie EV2 [file 44318_2026_771_MOESM4_ESM.zip › Fig 2/Fig 2J MMP FRET n1.tif]

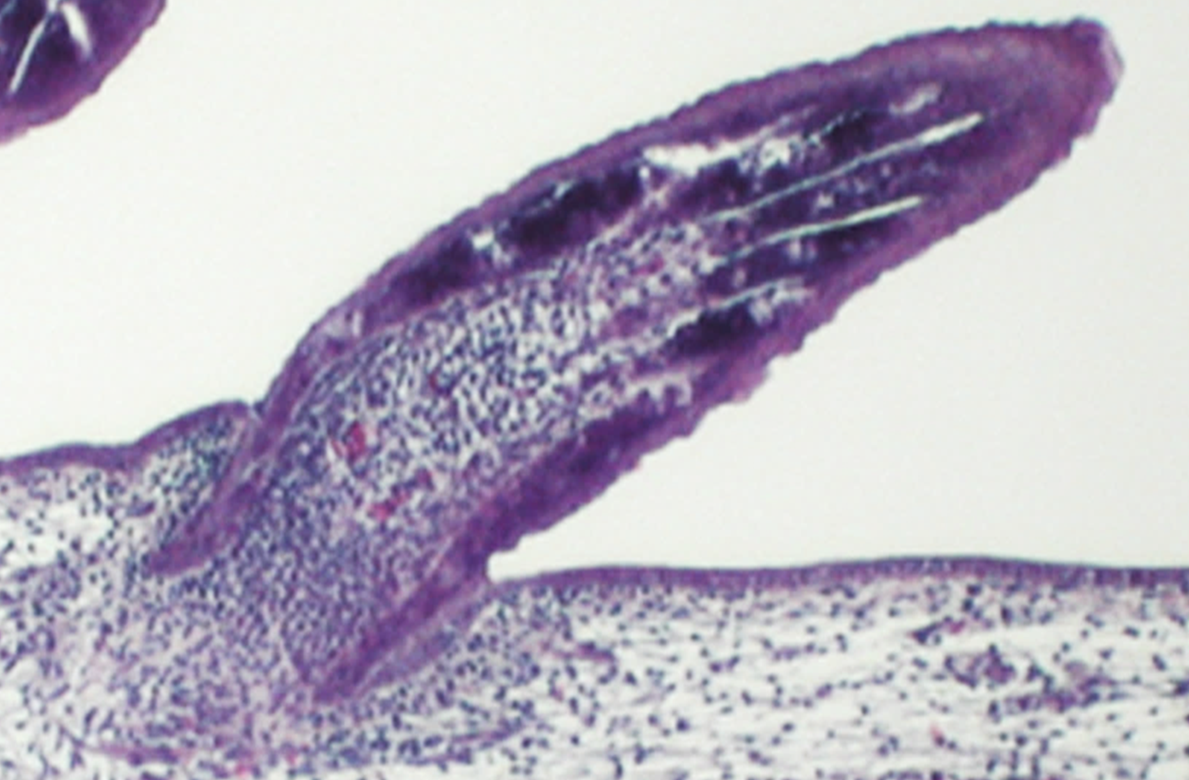

Supplement: Supplementary file 4 — Movie EV2 [file 44318_2026_771_MOESM4_ESM.zip › Fig 2/Fig 2A inv E12 n2.tif]

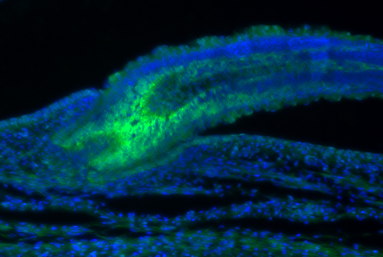

Supplement: Supplementary file 4 — Movie EV2 [file 44318_2026_771_MOESM4_ESM.zip › Fig 2/Yap n-3.tif]

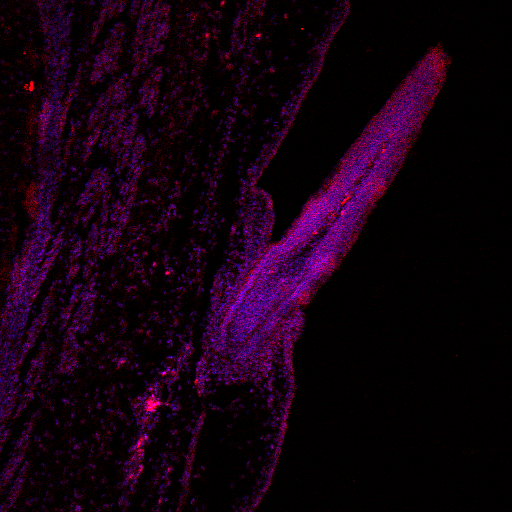

Supplement: Supplementary file 4 — Movie EV2 [file 44318_2026_771_MOESM4_ESM.zip › Fig 2/Fig 2K dnYAP MMP n1.tif]

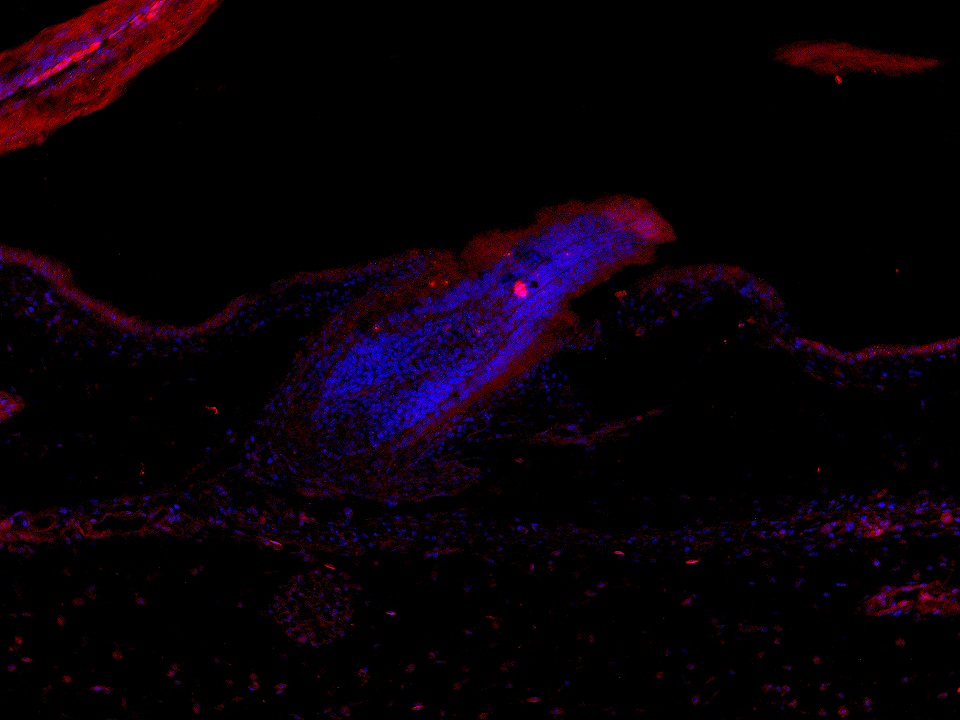

Supplement: Supplementary file 4 — Movie EV2 [file 44318_2026_771_MOESM4_ESM.zip › Fig 2/Fig 2k dnYAP MMP n3.tif]

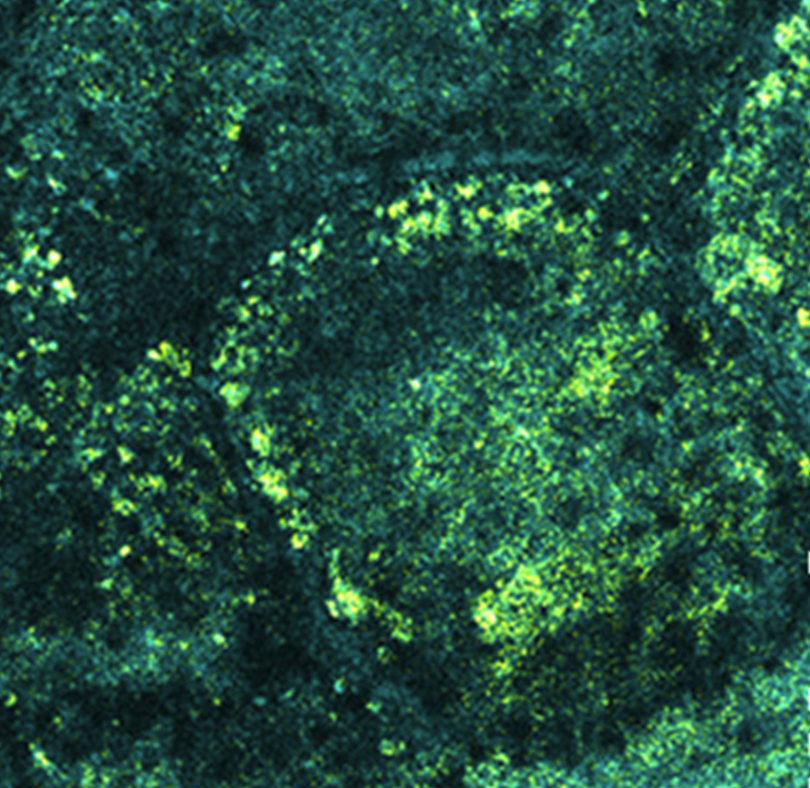

Supplement: Supplementary file 4 — Movie EV2 [file 44318_2026_771_MOESM4_ESM.zip › Fig 2/Fig 2J MMP FRET n2.tif]

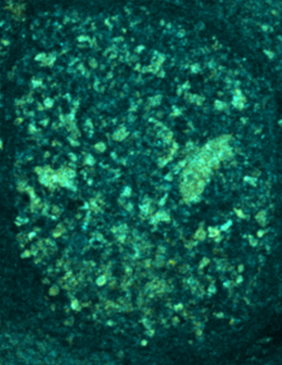

Supplement: Supplementary file 4 — Movie EV2 [file 44318_2026_771_MOESM4_ESM.zip › Fig 2/Fig 2J MMP FRET n3.tif]

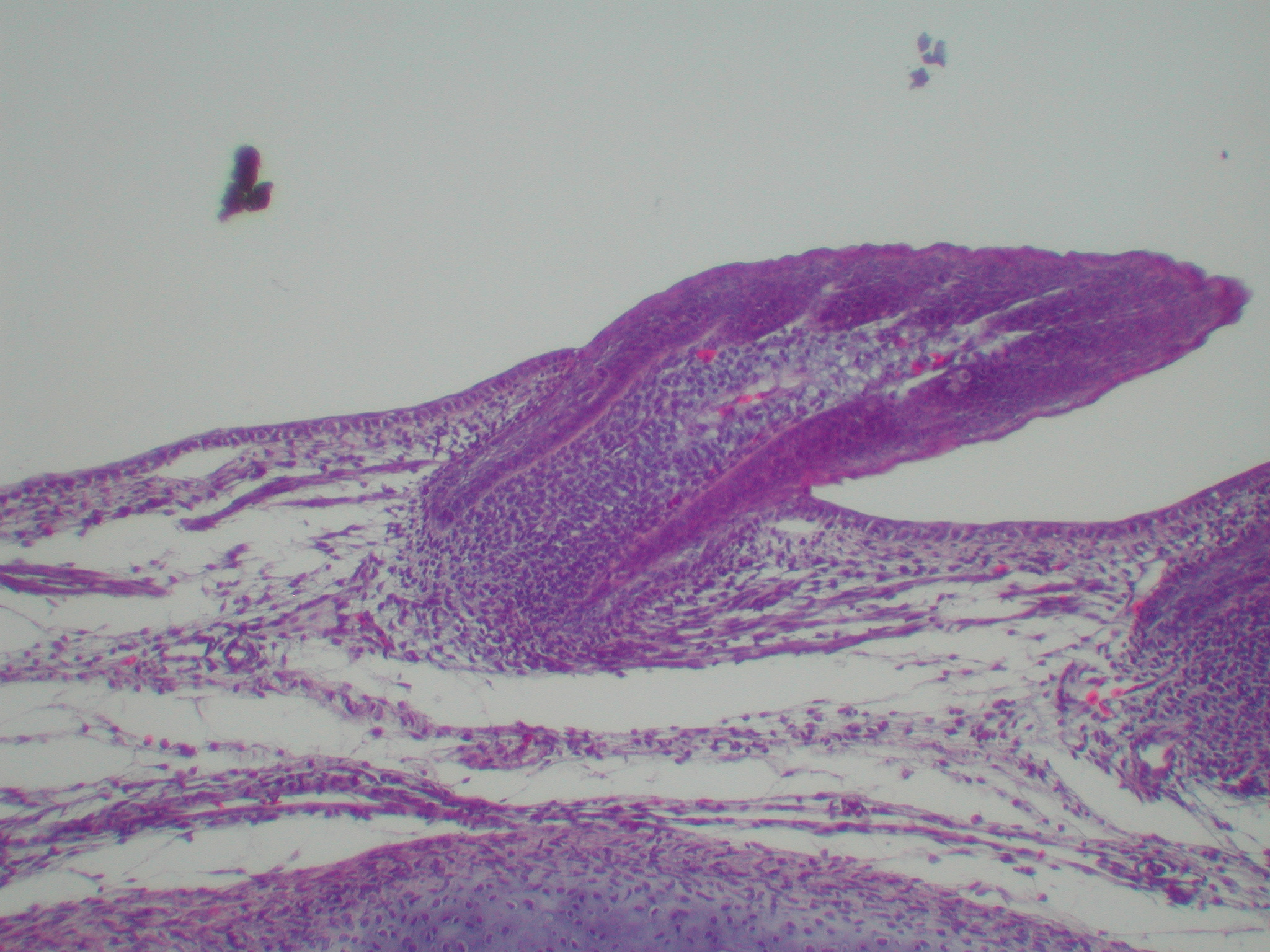

Supplement: Supplementary file 4 — Movie EV2 [file 44318_2026_771_MOESM4_ESM.zip › Fig 2/Fig 2A inv E12 n1.tif]

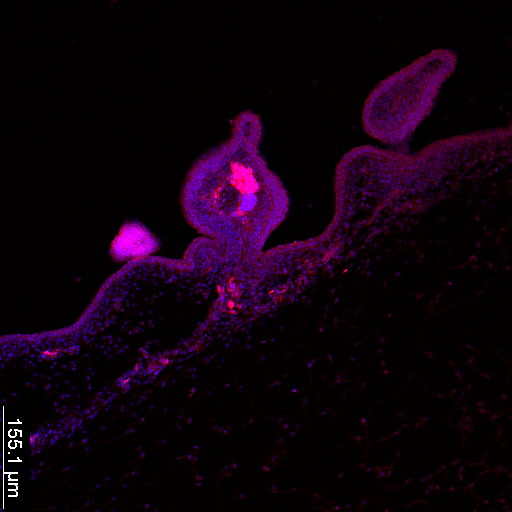

Supplement: Supplementary file 4 — Movie EV2 [file 44318_2026_771_MOESM4_ESM.zip › Fig 2/Fig 2k dnYAP MMP n2.tif]

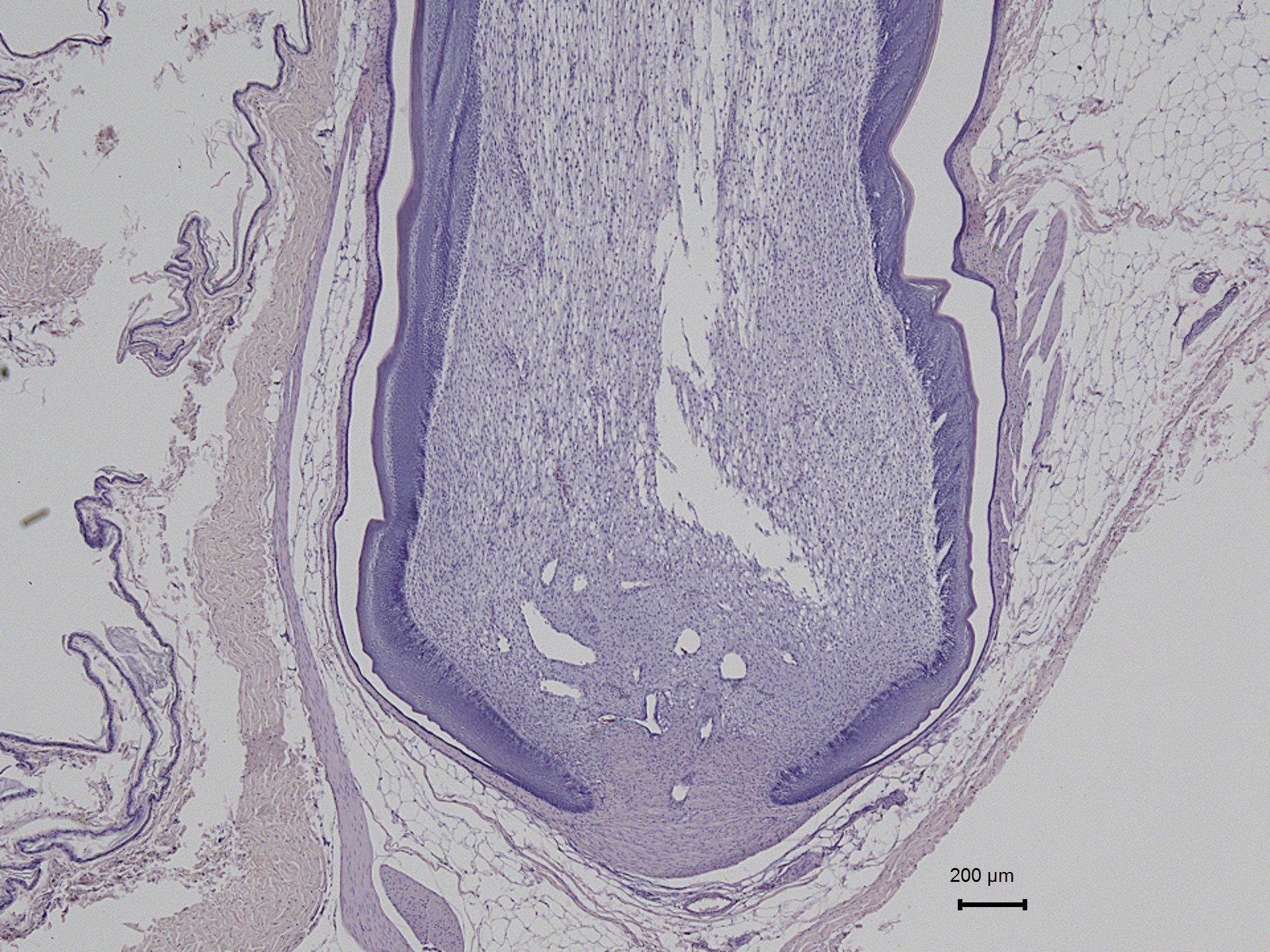

Supplement: Supplementary file 5 — Movie EV3 [file 44318_2026_771_MOESM5_ESM.zip › Fig 3/Fig 3C DP n2.tif]

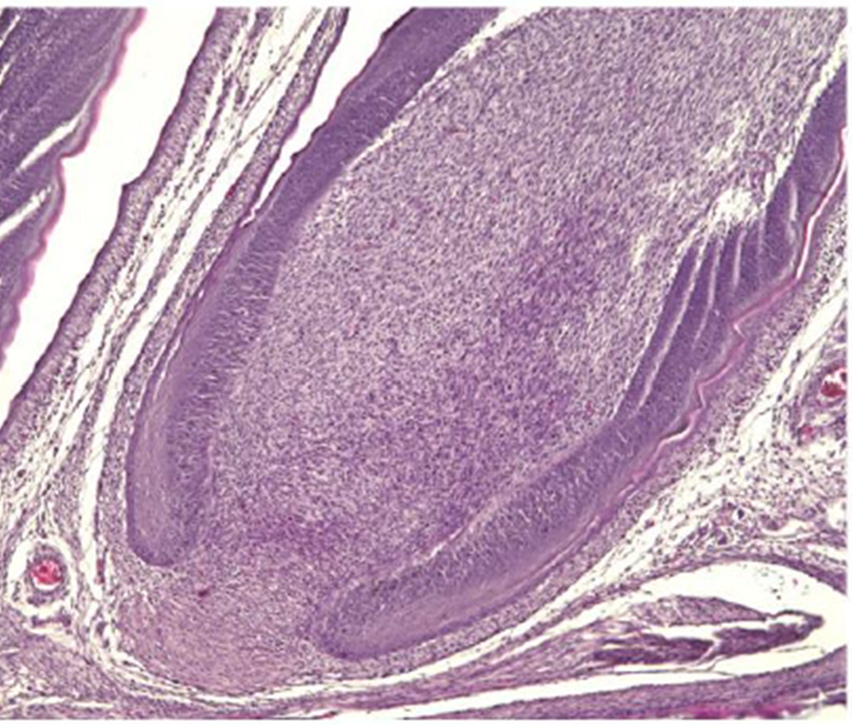

Supplement: Supplementary file 5 — Movie EV3 [file 44318_2026_771_MOESM5_ESM.zip › Fig 3/Fig 3C DP n1.tif]

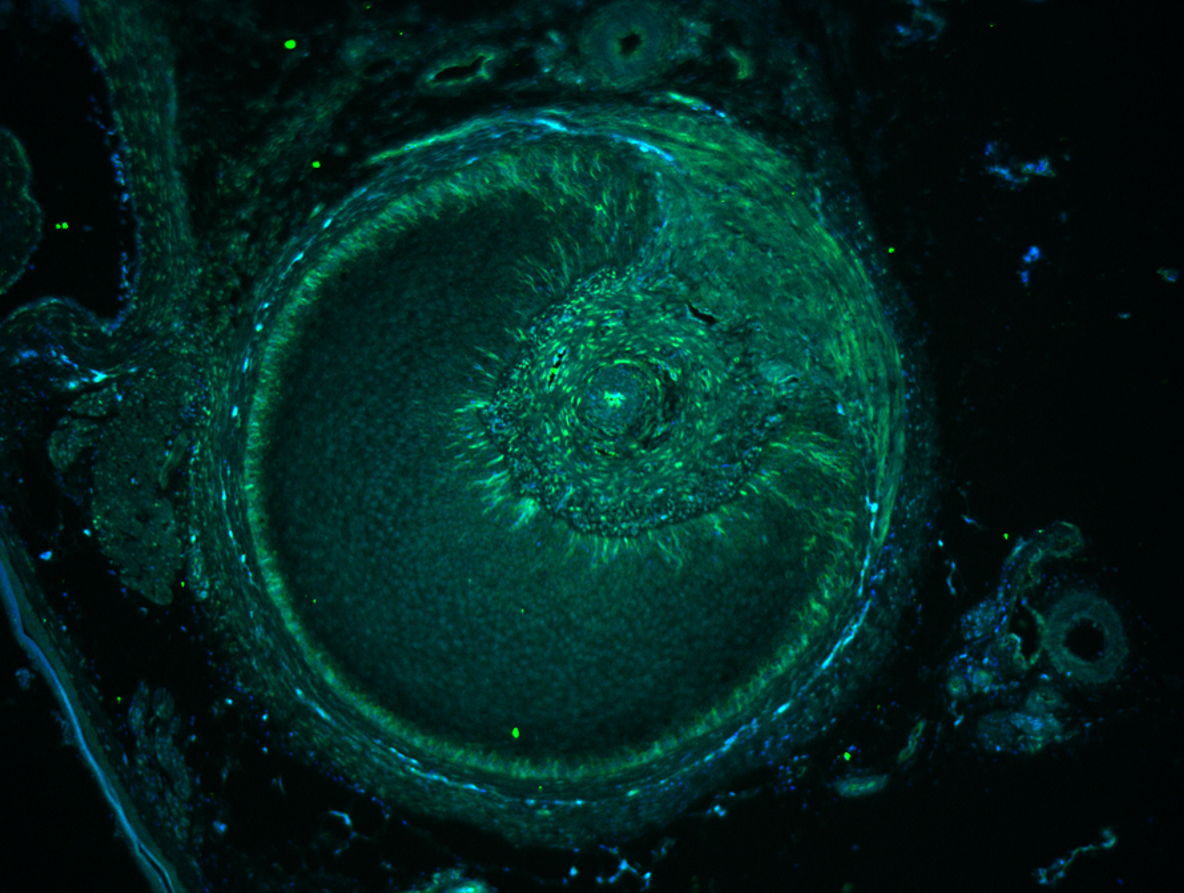

Supplement: Supplementary file 5 — Movie EV3 [file 44318_2026_771_MOESM5_ESM.zip › Fig 3/Fig 3H Yap.tif]

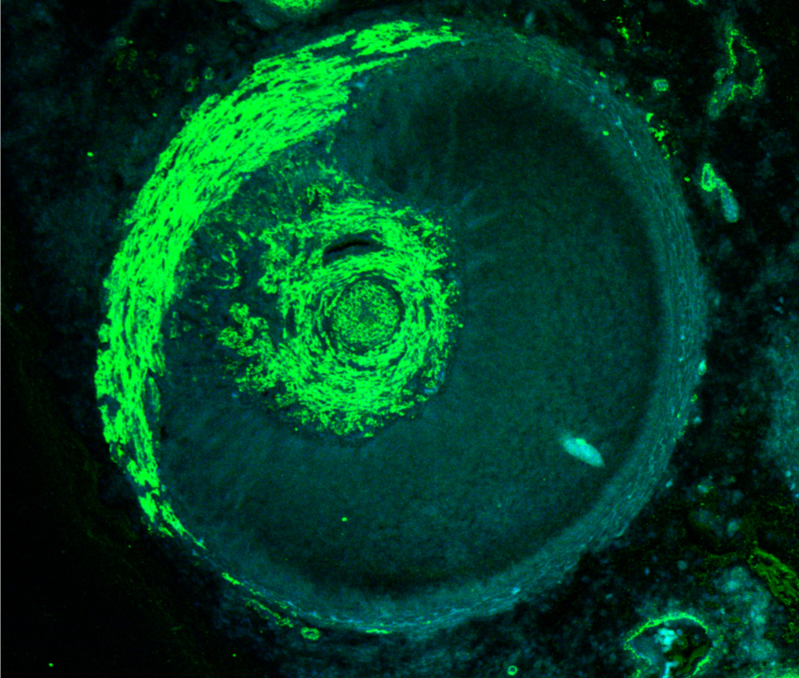

Supplement: Supplementary file 5 — Movie EV3 [file 44318_2026_771_MOESM5_ESM.zip › Fig 3/Fig 3H Yap n2.tif]

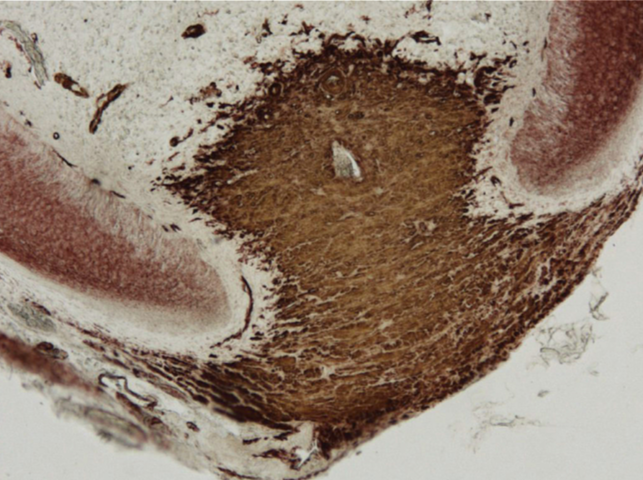

Supplement: Supplementary file 5 — Movie EV3 [file 44318_2026_771_MOESM5_ESM.zip › Fig 3/3G SMA NB DP n5.tif]

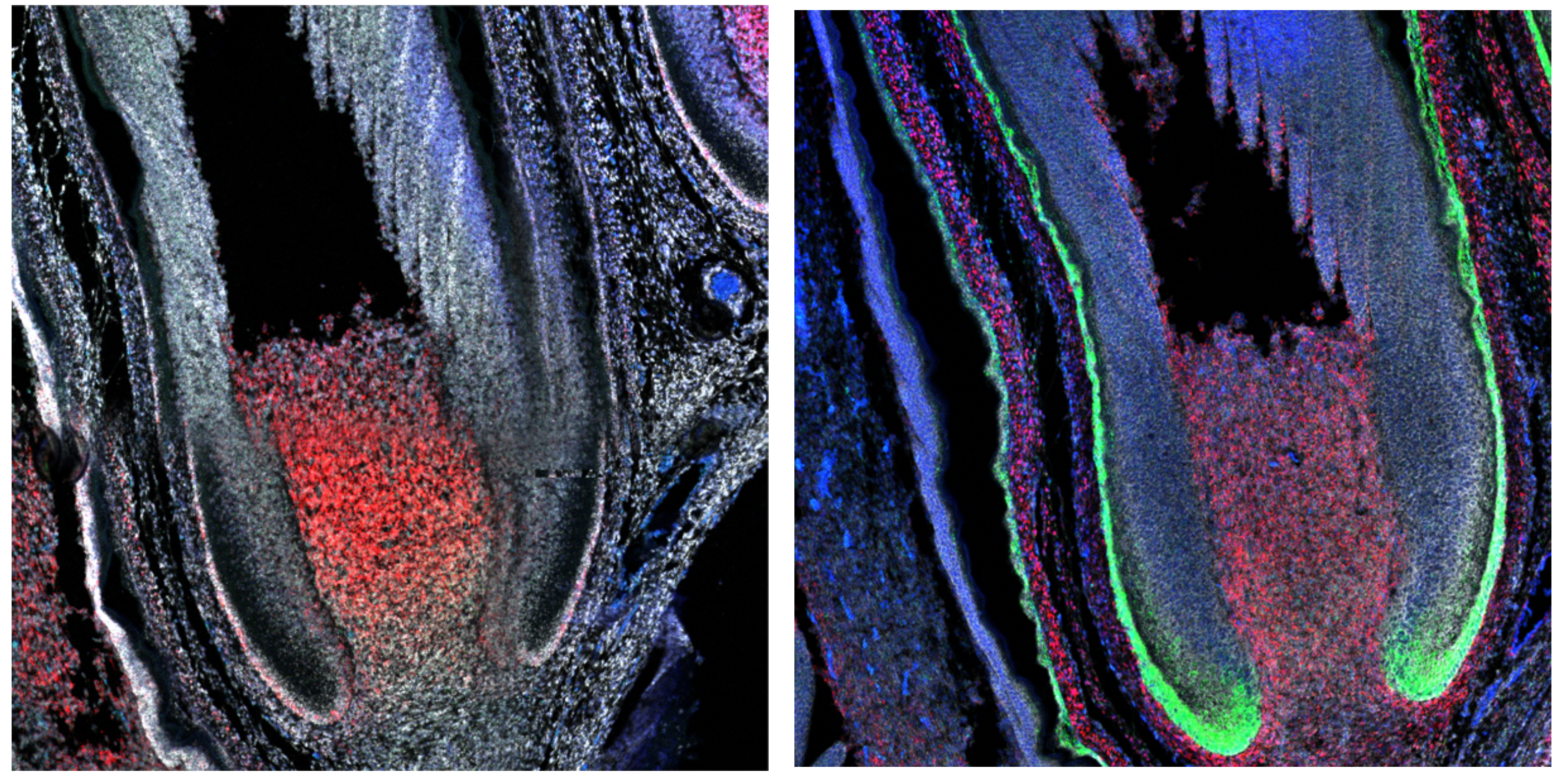

Supplement: Supplementary file 5 — Movie EV3 [file 44318_2026_771_MOESM5_ESM.zip › Fig 3/Fig 3H n2.tiff]

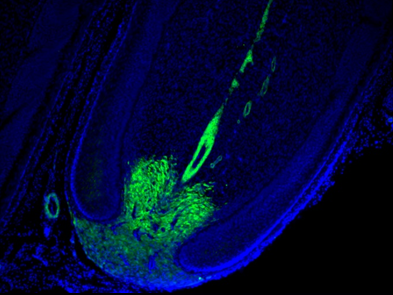

Supplement: Supplementary file 5 — Movie EV3 [file 44318_2026_771_MOESM5_ESM.zip › Fig 3/3G SMA NB DP n4.tif]

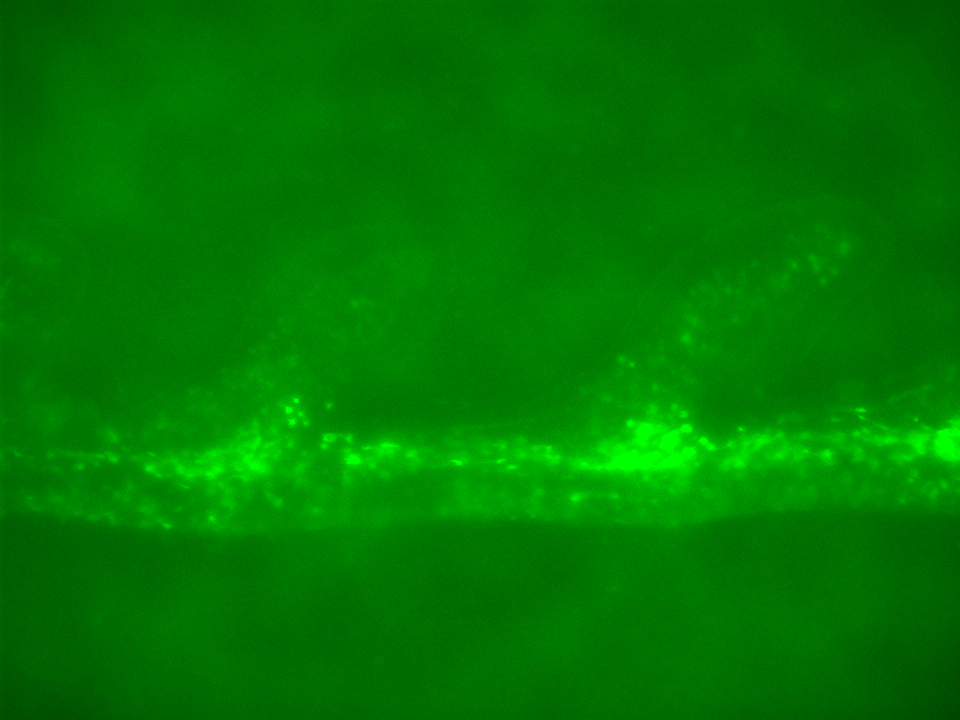

Supplement: Supplementary file 5 — Movie EV3 [file 44318_2026_771_MOESM5_ESM.zip › Fig 3/Fig 3A 18h-1 .tif]

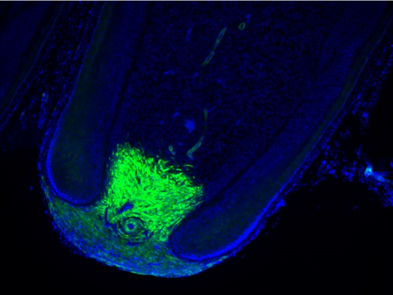

Supplement: Supplementary file 5 — Movie EV3 [file 44318_2026_771_MOESM5_ESM.zip › Fig 3/3G SMA NB DP n3.tif]

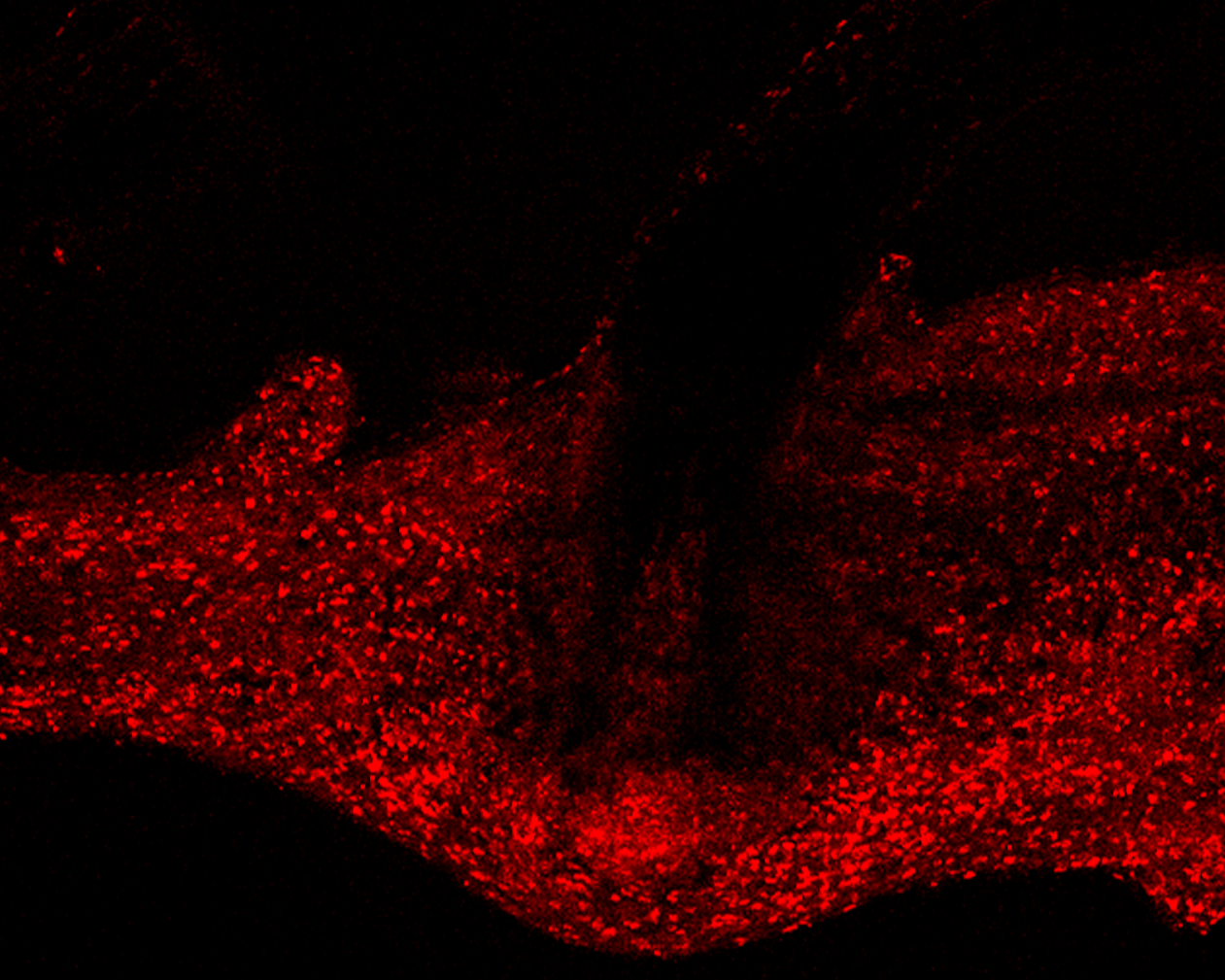

Supplement: Supplementary file 5 — Movie EV3 [file 44318_2026_771_MOESM5_ESM.zip › Fig 3/Fig 3D DP n3.tif]

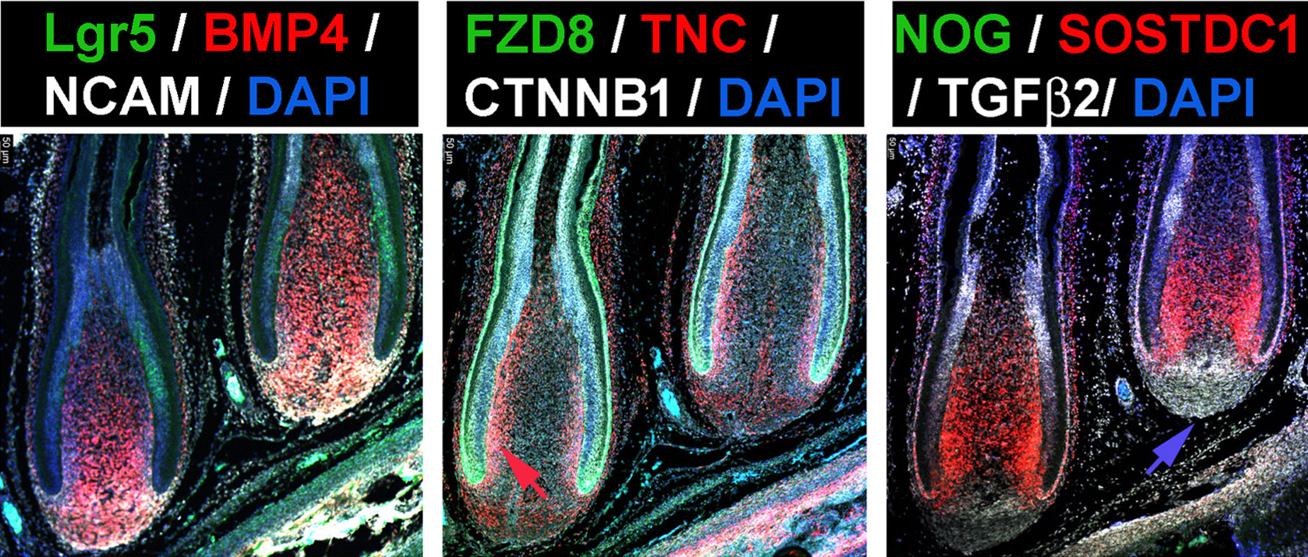

Supplement: Supplementary file 5 — Movie EV3 [file 44318_2026_771_MOESM5_ESM.zip › Fig 3/Fig 3H FTCNST n2.tif]

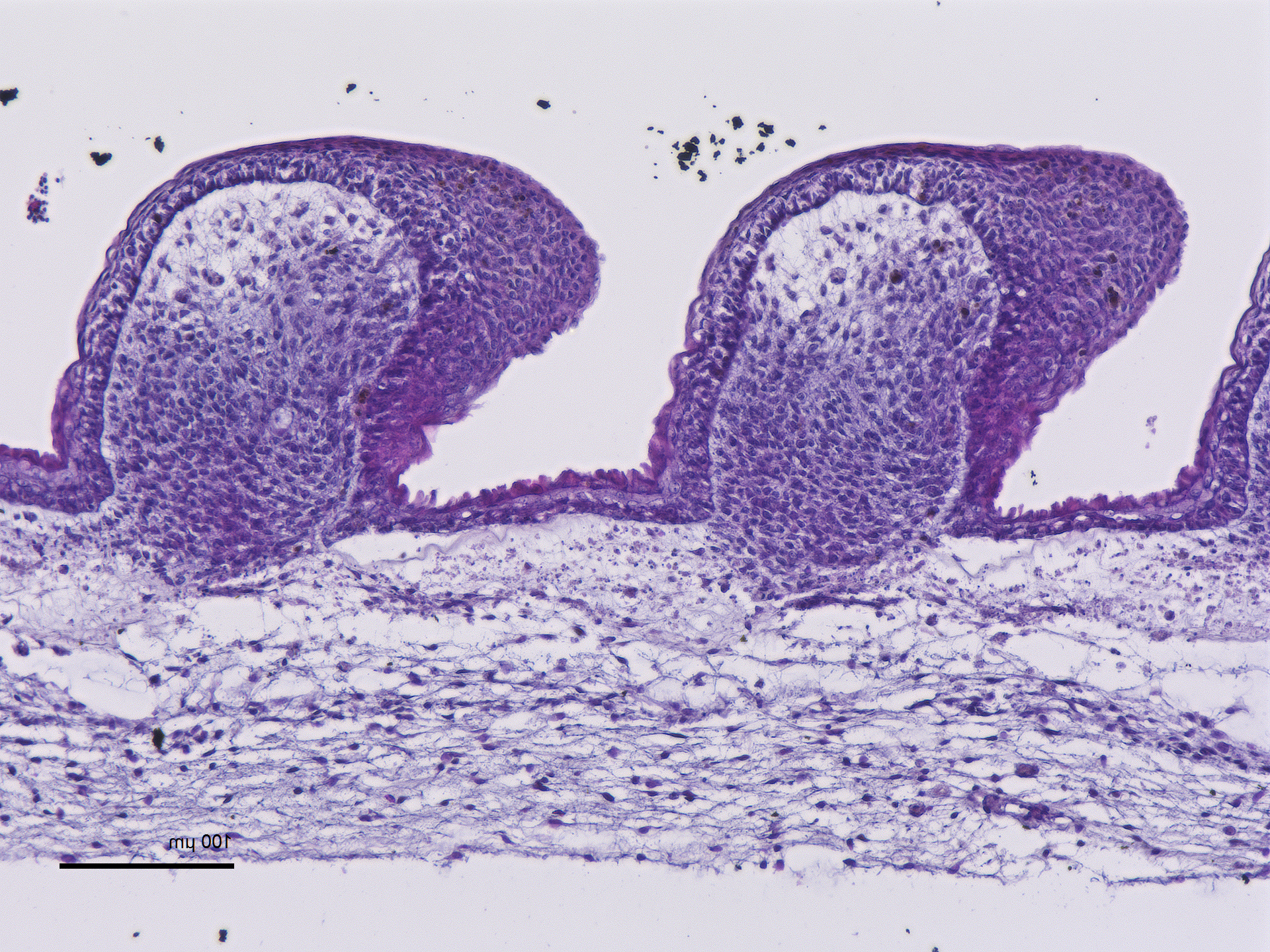

Supplement: Supplementary file 5 — Movie EV3 [file 44318_2026_771_MOESM5_ESM.zip › Fig 3/3K 3 Ly2106971_02.tif]

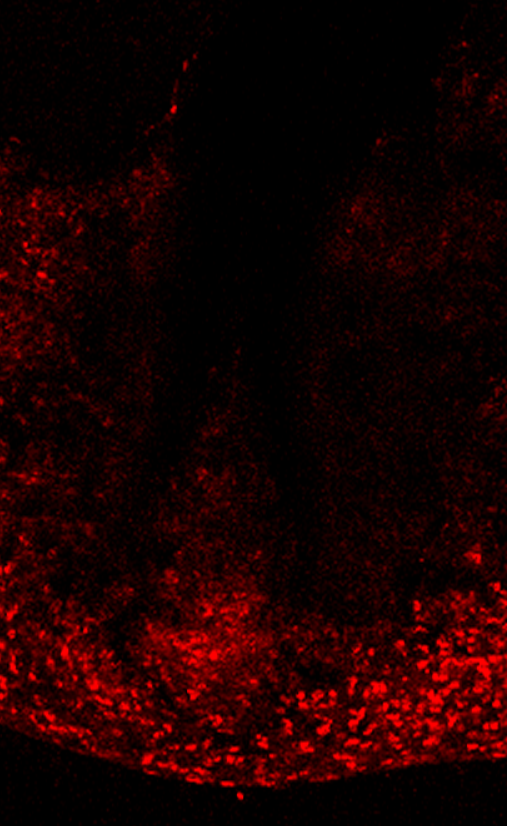

Supplement: Supplementary file 5 — Movie EV3 [file 44318_2026_771_MOESM5_ESM.zip › Fig 3/Fig 3D DP n2.tif]

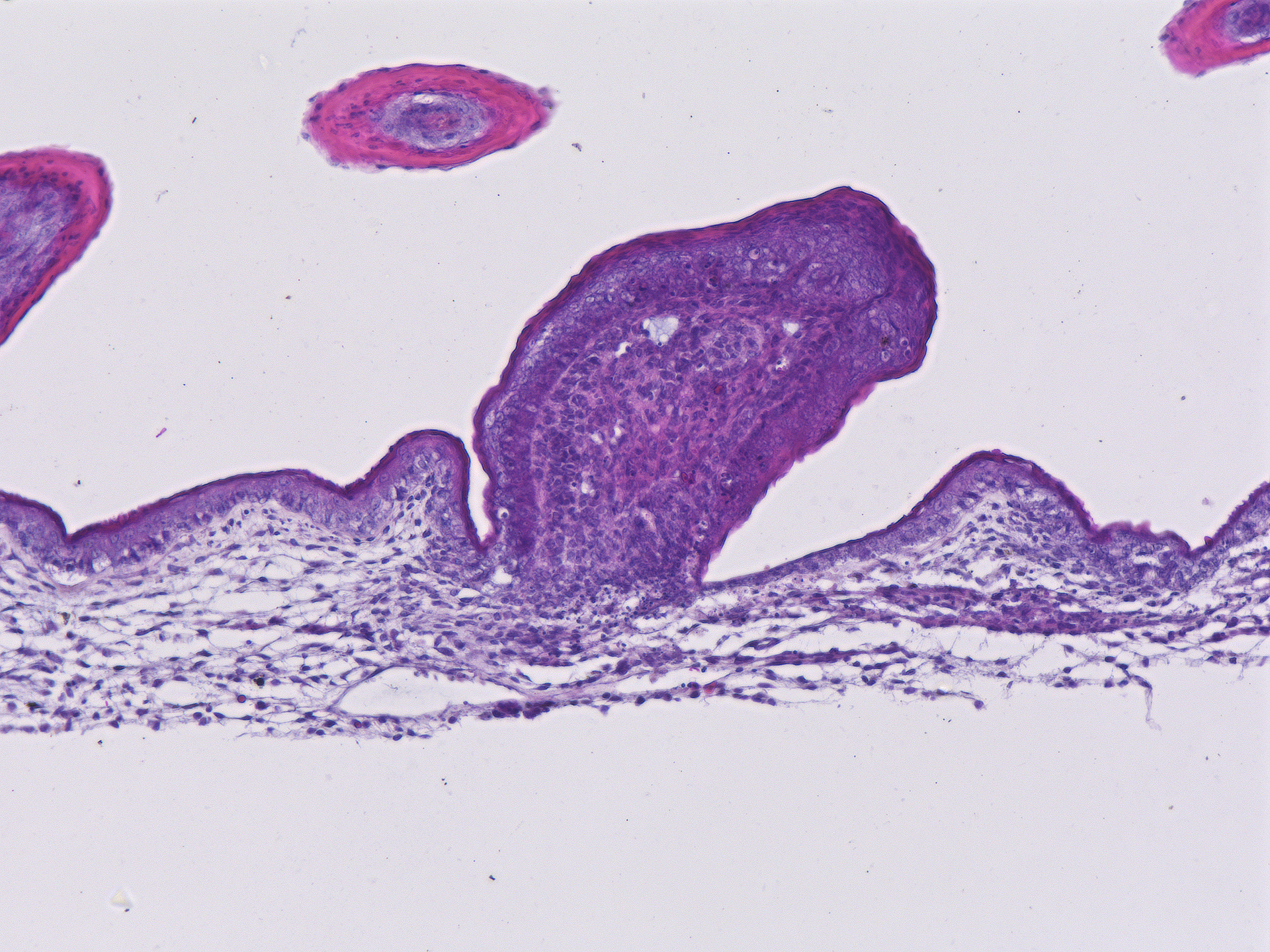

Supplement: Supplementary file 5 — Movie EV3 [file 44318_2026_771_MOESM5_ESM.zip › Fig 3/3K 2 Ly2106971 20uM_05.tif]

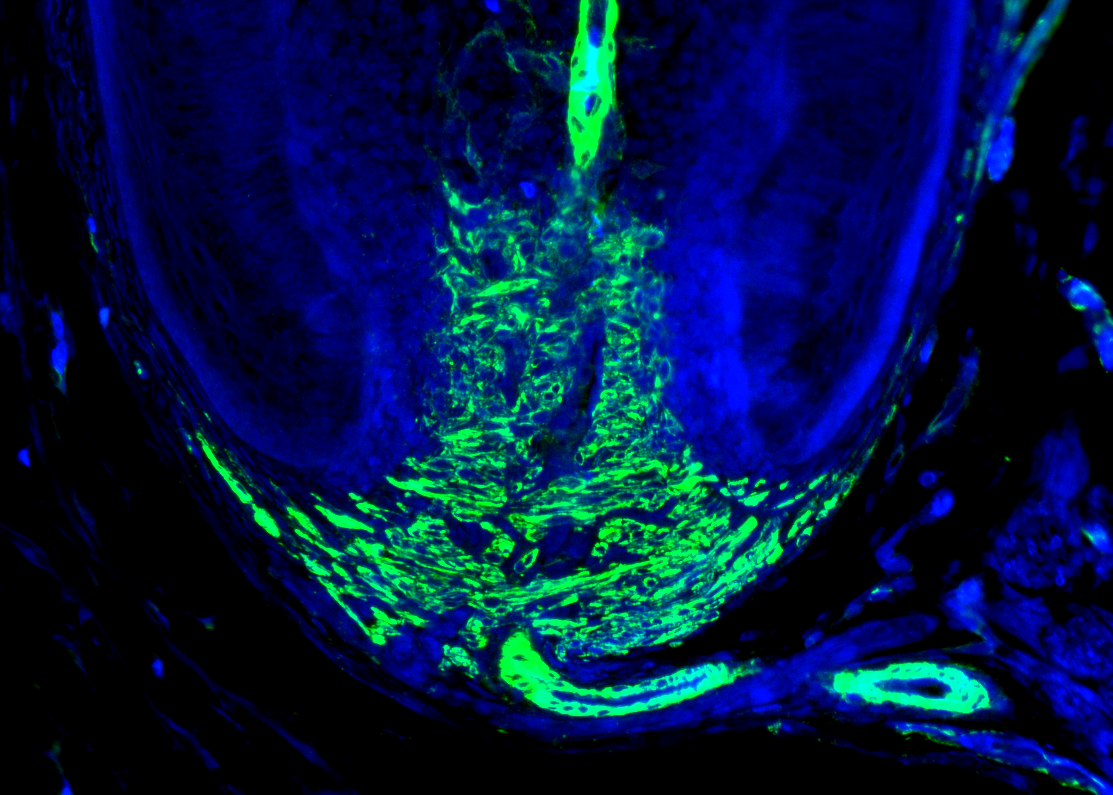

Supplement: Supplementary file 5 — Movie EV3 [file 44318_2026_771_MOESM5_ESM.zip › Fig 3/3G SMA NB DP n2.tif]

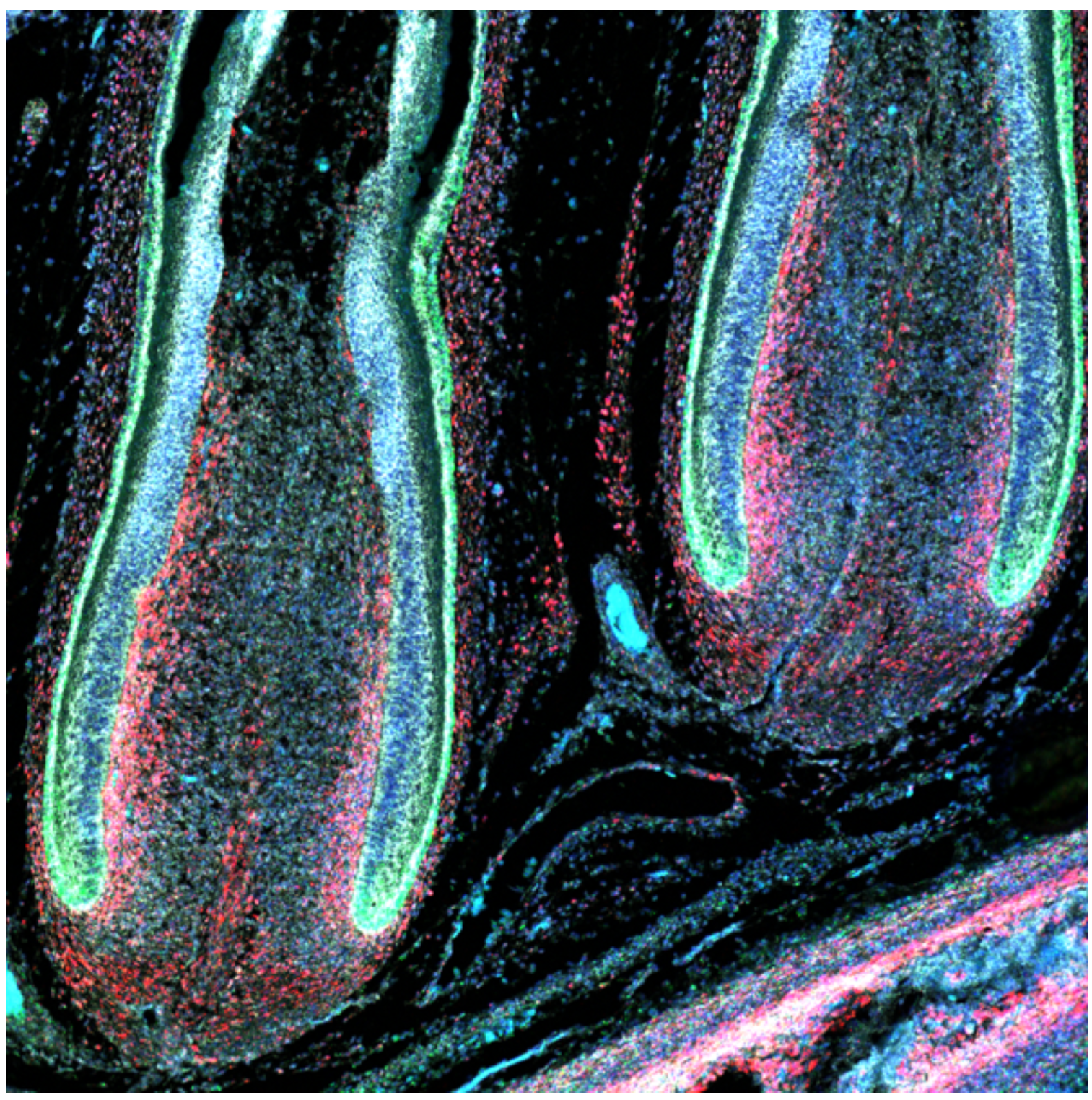

Supplement: Supplementary file 5 — Movie EV3 [file 44318_2026_771_MOESM5_ESM.zip › Fig 3/Fig 3H n3.tiff]

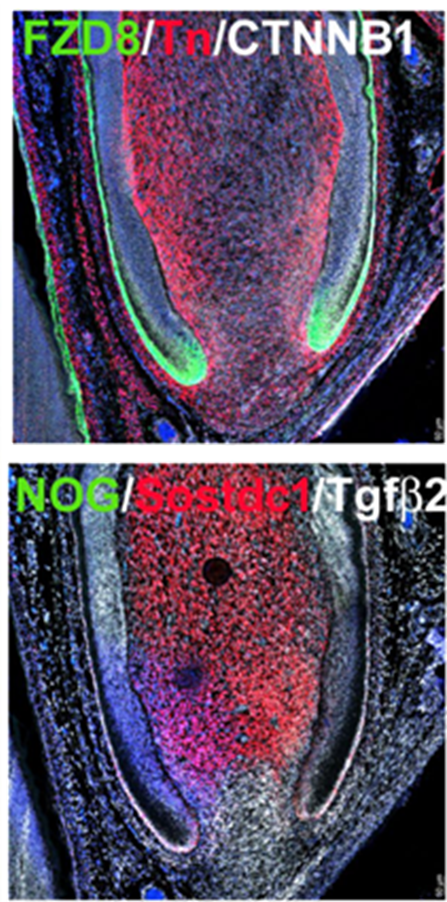

Supplement: Supplementary file 5 — Movie EV3 [file 44318_2026_771_MOESM5_ESM.zip › Fig 3/Fig 3H FTCNST n1.tif]

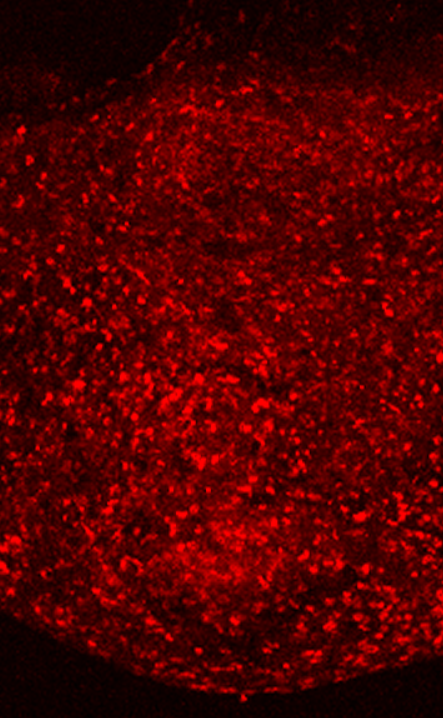

Supplement: Supplementary file 5 — Movie EV3 [file 44318_2026_771_MOESM5_ESM.zip › Fig 3/Fig 3D DP n1.tif]

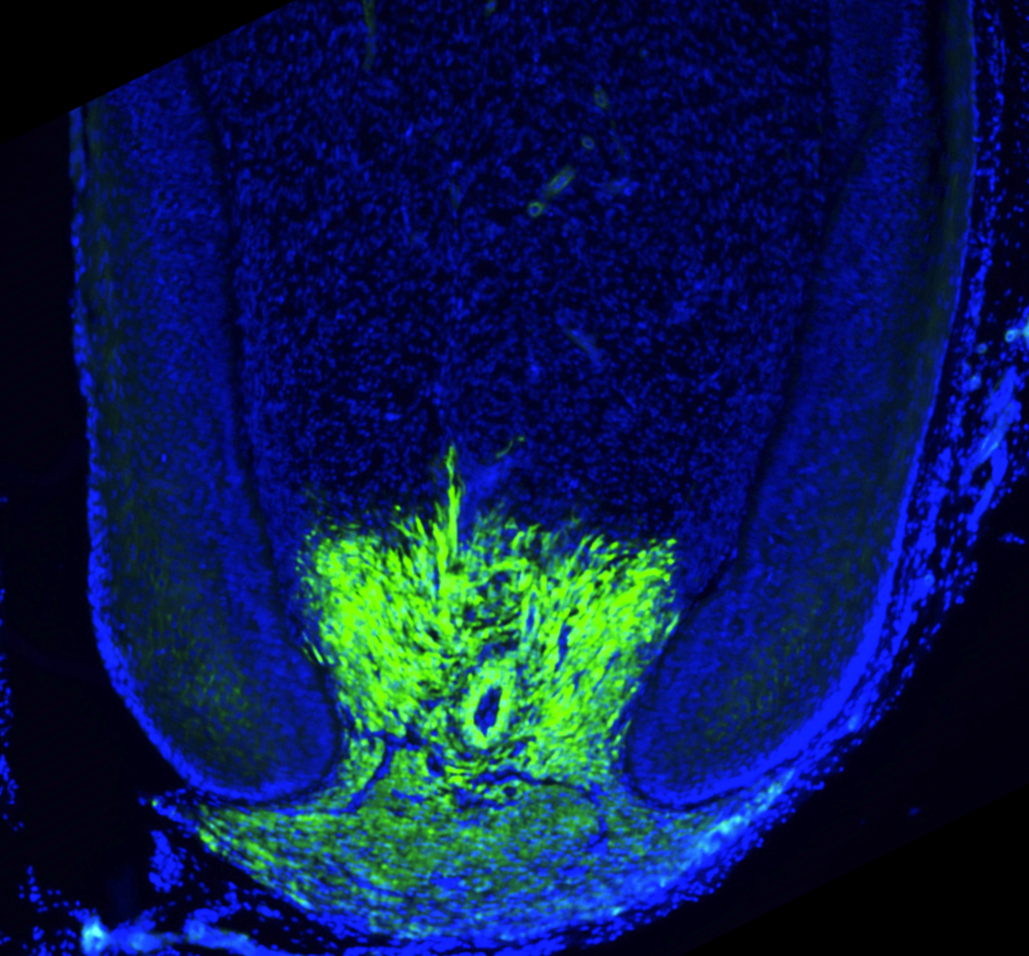

Supplement: Supplementary file 5 — Movie EV3 [file 44318_2026_771_MOESM5_ESM.zip › Fig 3/3G SMA NB DP n1.tif]

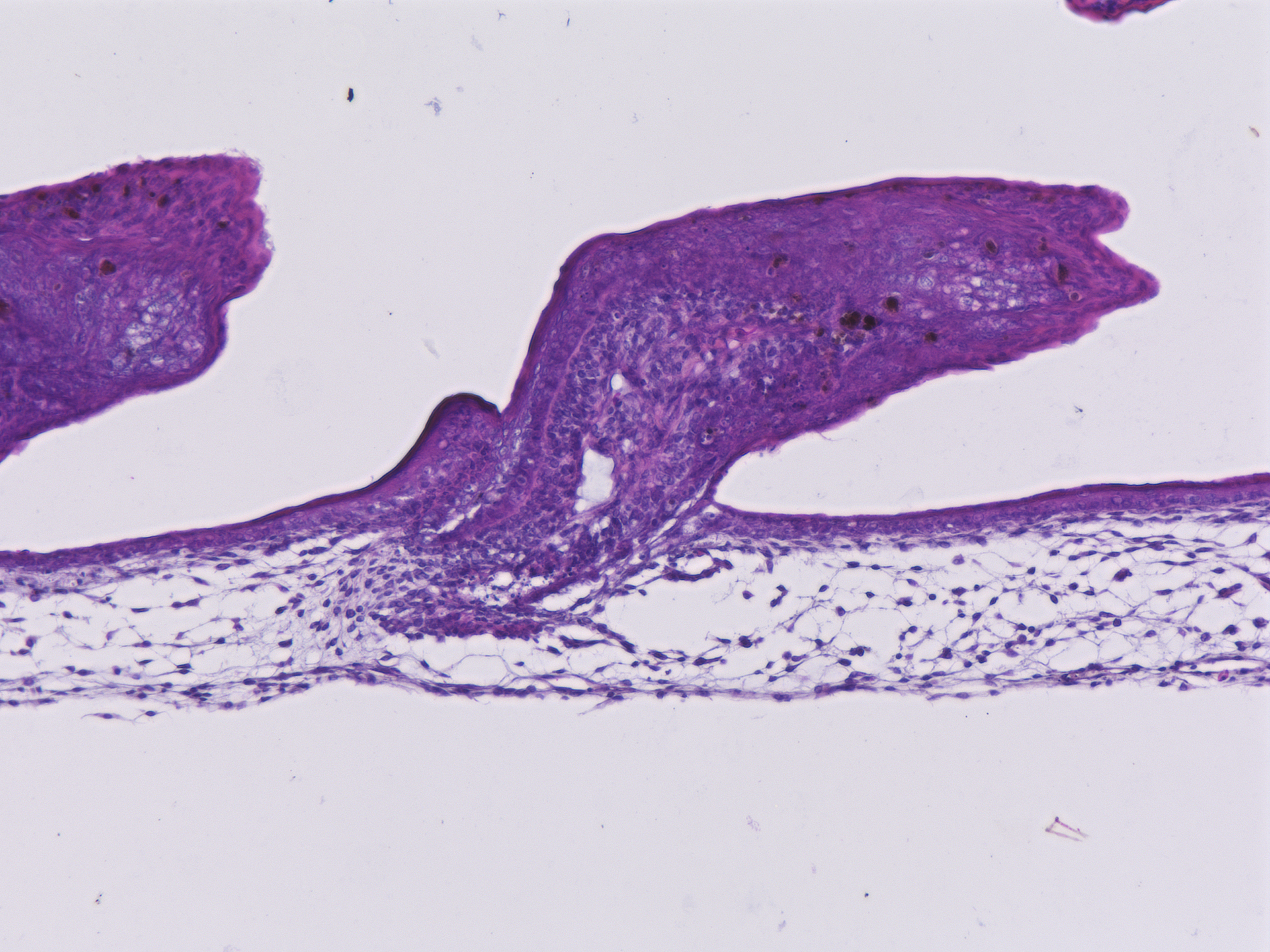

Supplement: Supplementary file 5 — Movie EV3 [file 44318_2026_771_MOESM5_ESM.zip › Fig 3/3K control_05.tif]

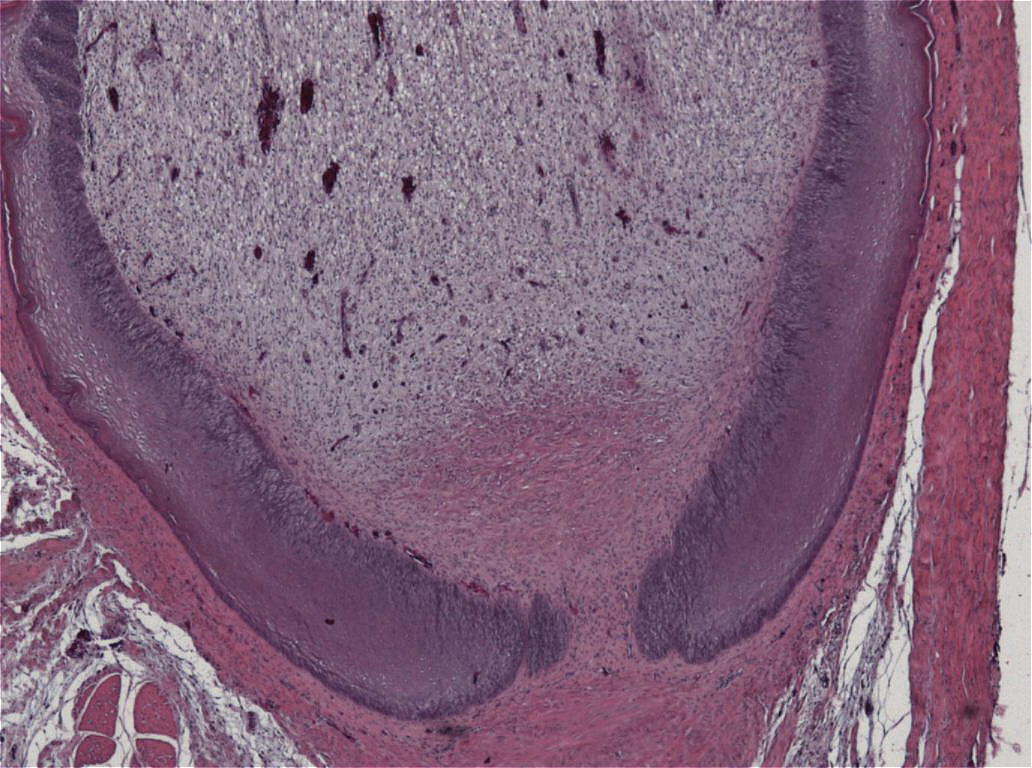

Supplement: Supplementary file 5 — Movie EV3 [file 44318_2026_771_MOESM5_ESM.zip › Fig 3/Fig 3A DP n3.tif]

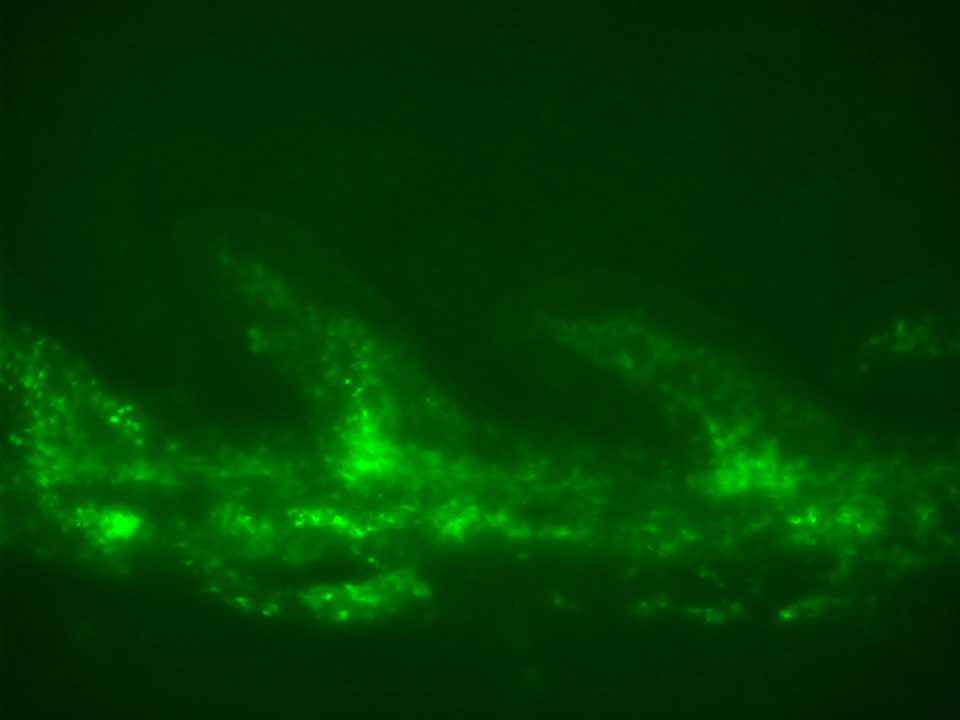

Supplement: Supplementary file 5 — Movie EV3 [file 44318_2026_771_MOESM5_ESM.zip › Fig 3/Fig 3A 18h-2.tif]

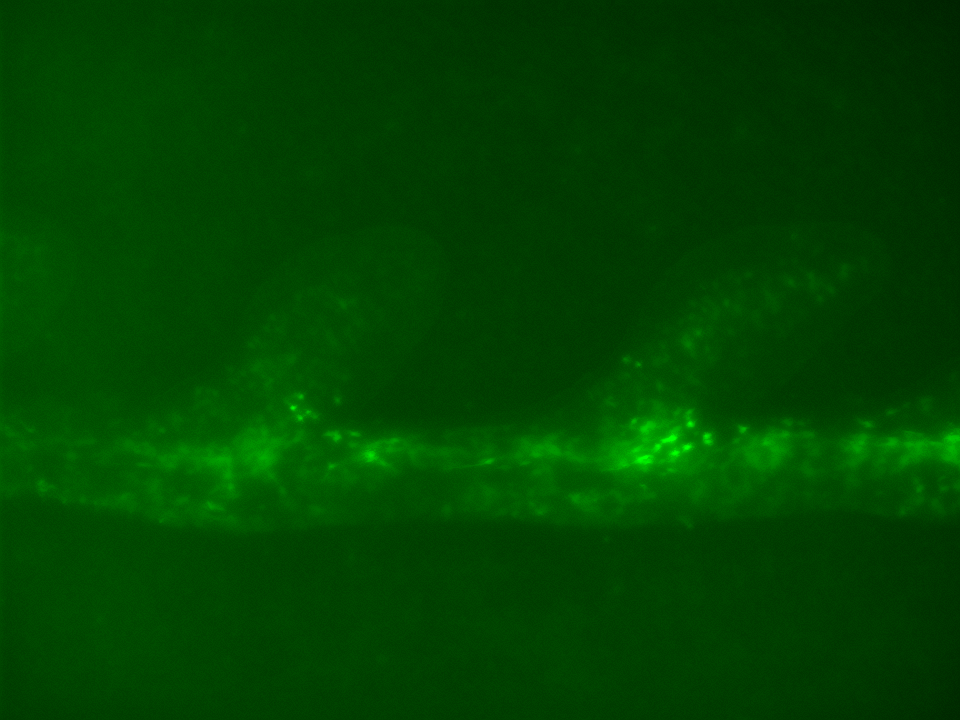

Supplement: Supplementary file 5 — Movie EV3 [file 44318_2026_771_MOESM5_ESM.zip › Fig 3/Fig 3A 0h-1.tif]

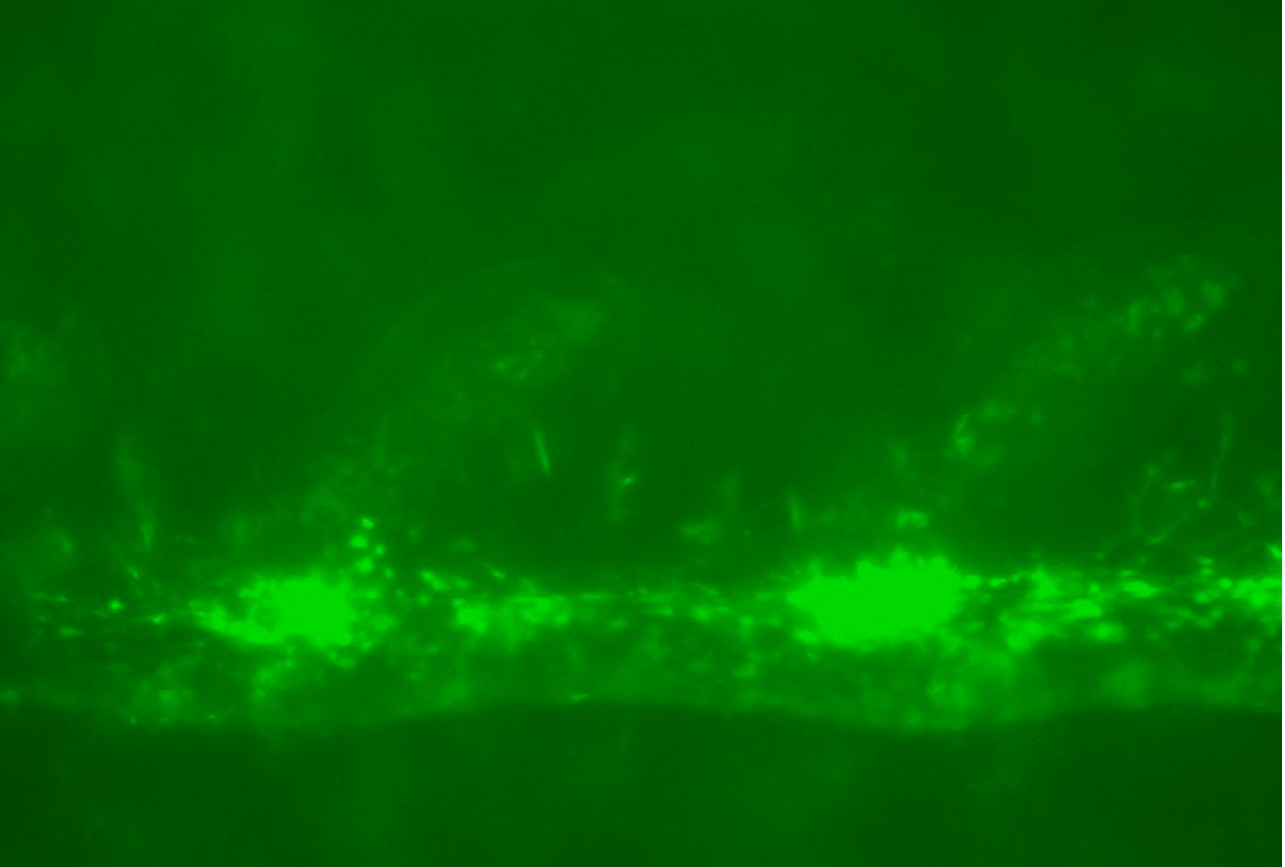

Supplement: Supplementary file 5 — Movie EV3 [file 44318_2026_771_MOESM5_ESM.zip › Fig 3/Fig 3A 18h-3.tif]

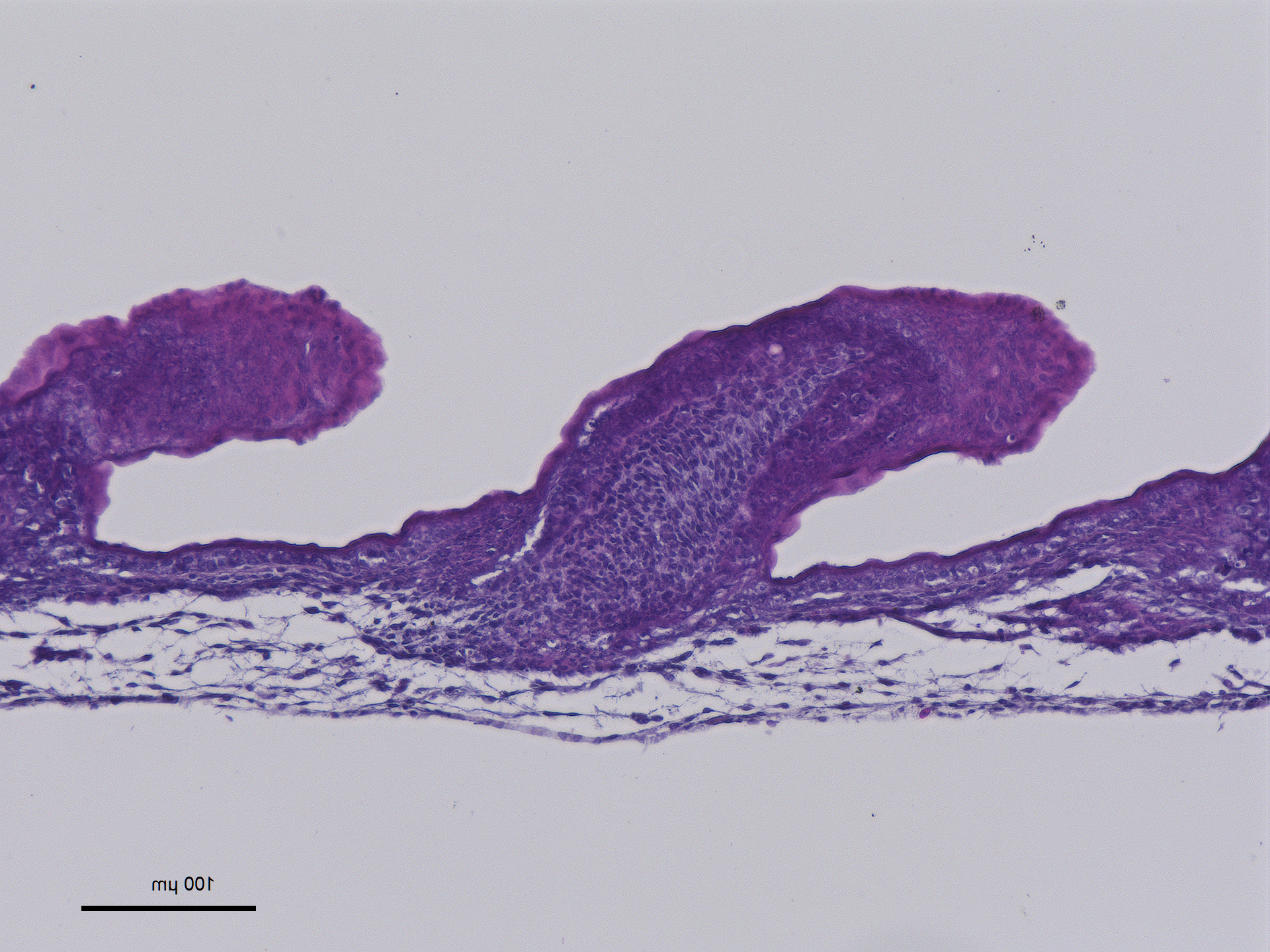

Supplement: Supplementary file 5 — Movie EV3 [file 44318_2026_771_MOESM5_ESM.zip › Fig 3/3K control_04.tif]

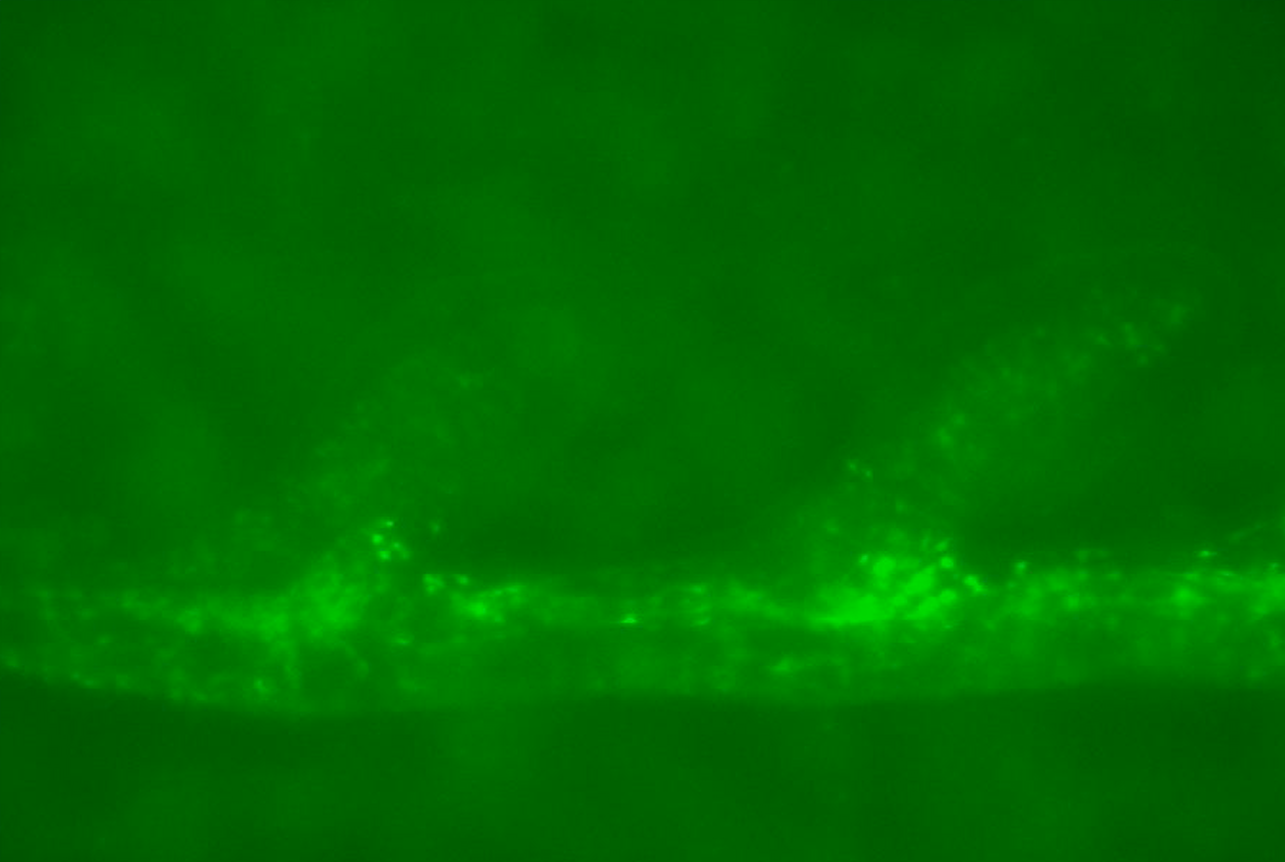

Supplement: Supplementary file 5 — Movie EV3 [file 44318_2026_771_MOESM5_ESM.zip › Fig 3/Fig 3A 0h-2.tif]

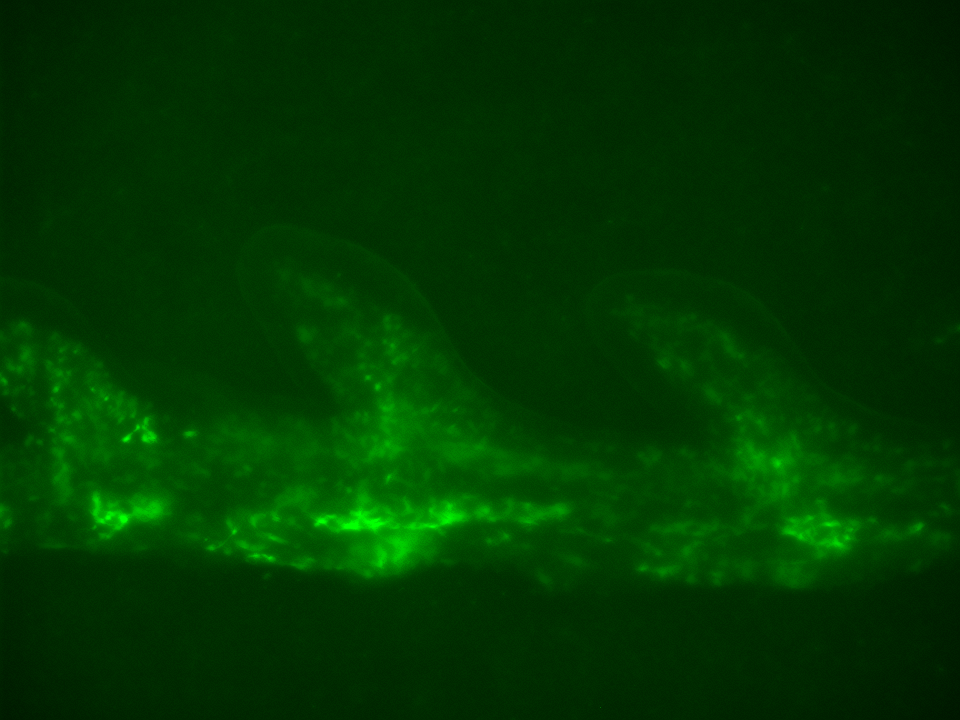

Supplement: Supplementary file 5 — Movie EV3 [file 44318_2026_771_MOESM5_ESM.zip › Fig 3/Fig 3A 0h-3.tif]

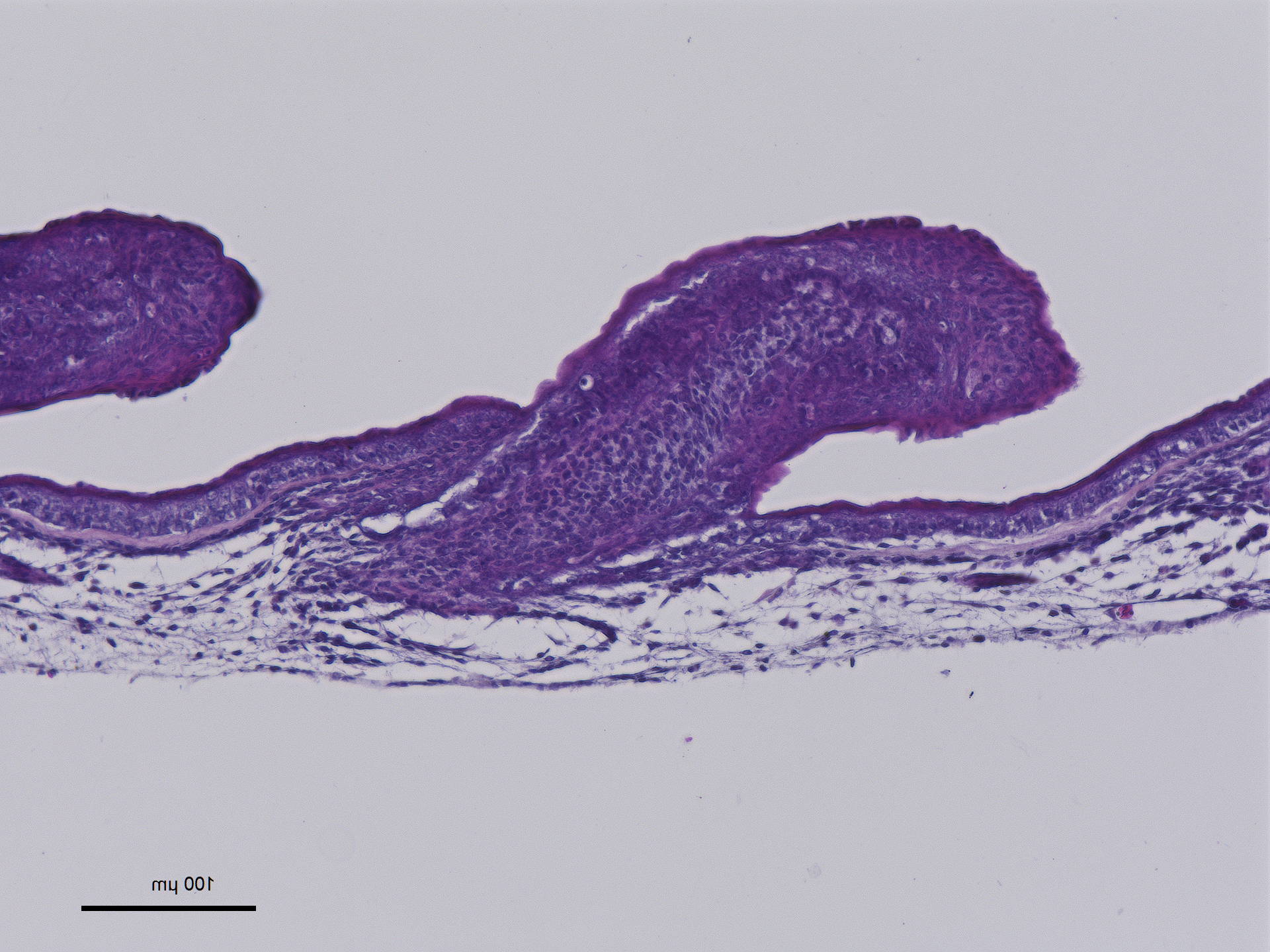

Supplement: Supplementary file 5 — Movie EV3 [file 44318_2026_771_MOESM5_ESM.zip › Fig 3/3K control_03.tif]

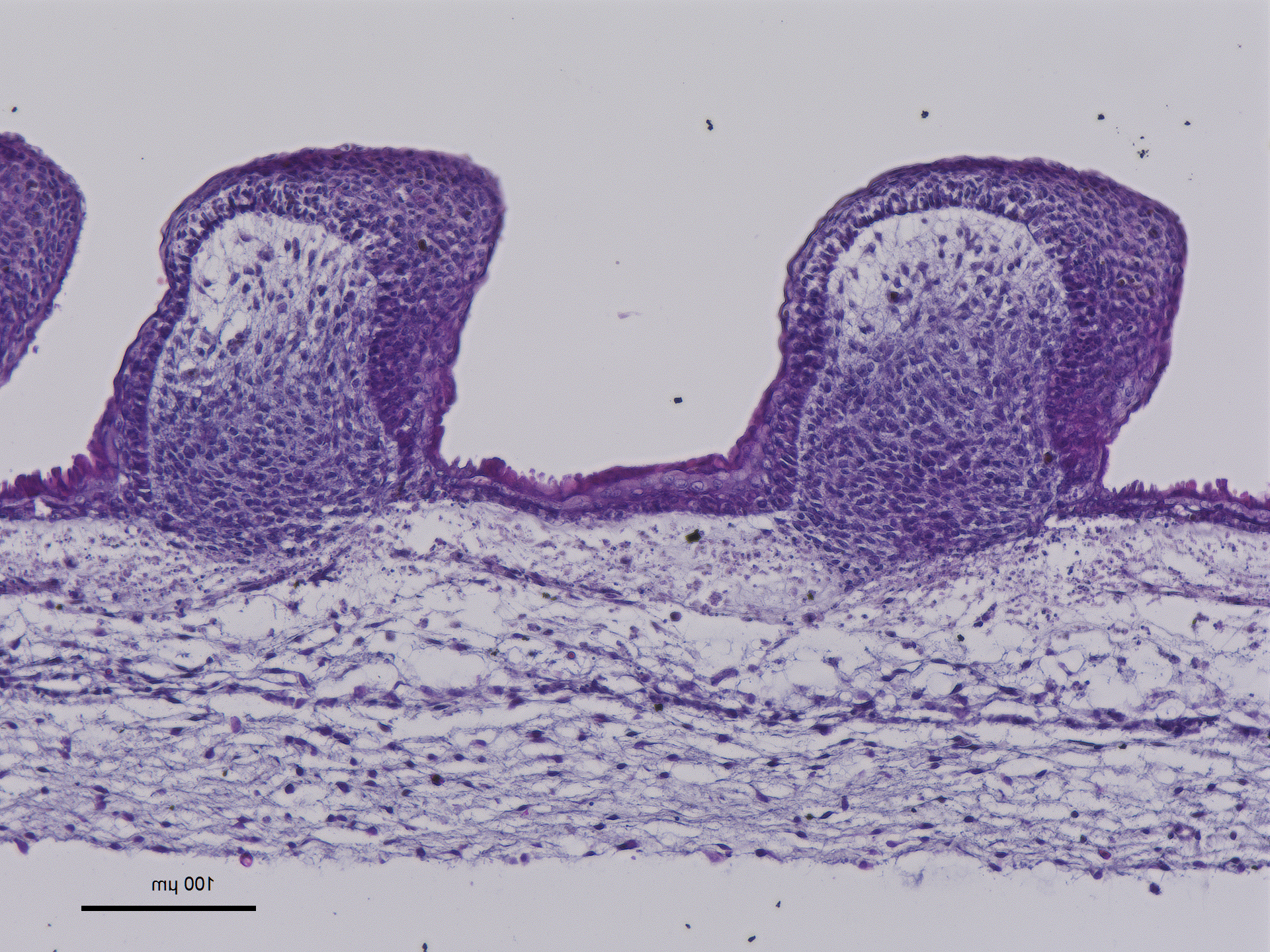

Supplement: Supplementary file 5 — Movie EV3 [file 44318_2026_771_MOESM5_ESM.zip › Fig 3/3K 4 Ly2106971_05.tif]

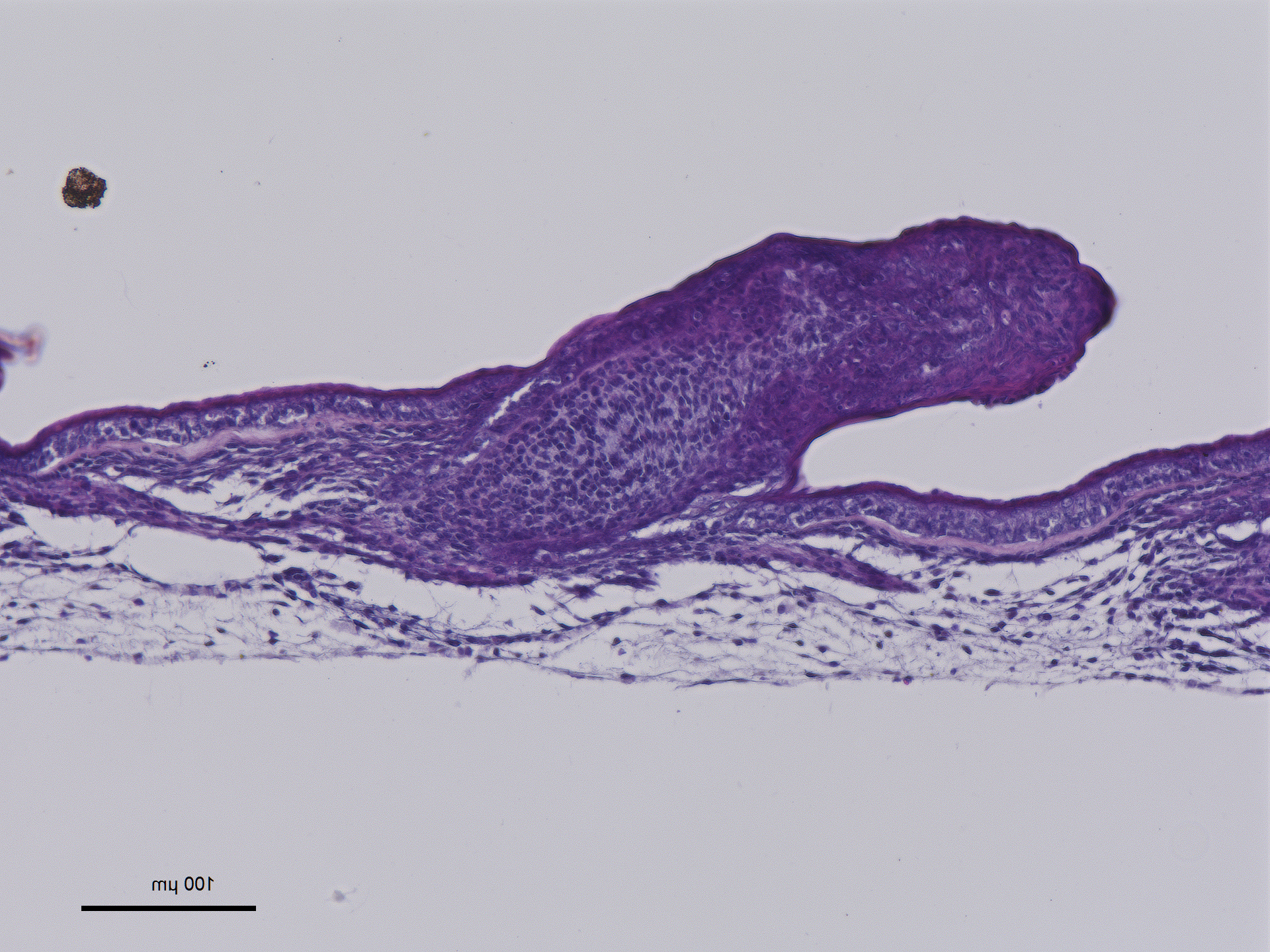

Supplement: Supplementary file 5 — Movie EV3 [file 44318_2026_771_MOESM5_ESM.zip › Fig 3/3K control_02.tif]

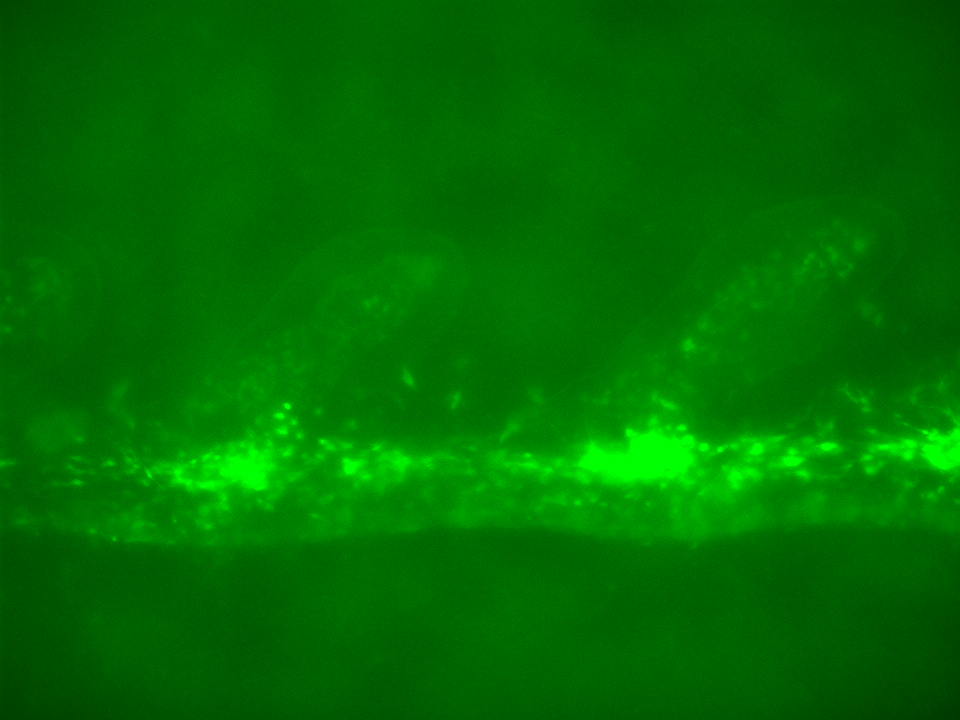

Supplement: Supplementary file 5 — Movie EV3 [file 44318_2026_771_MOESM5_ESM.zip › Fig 3/Fig 3A 36h-1.tif]

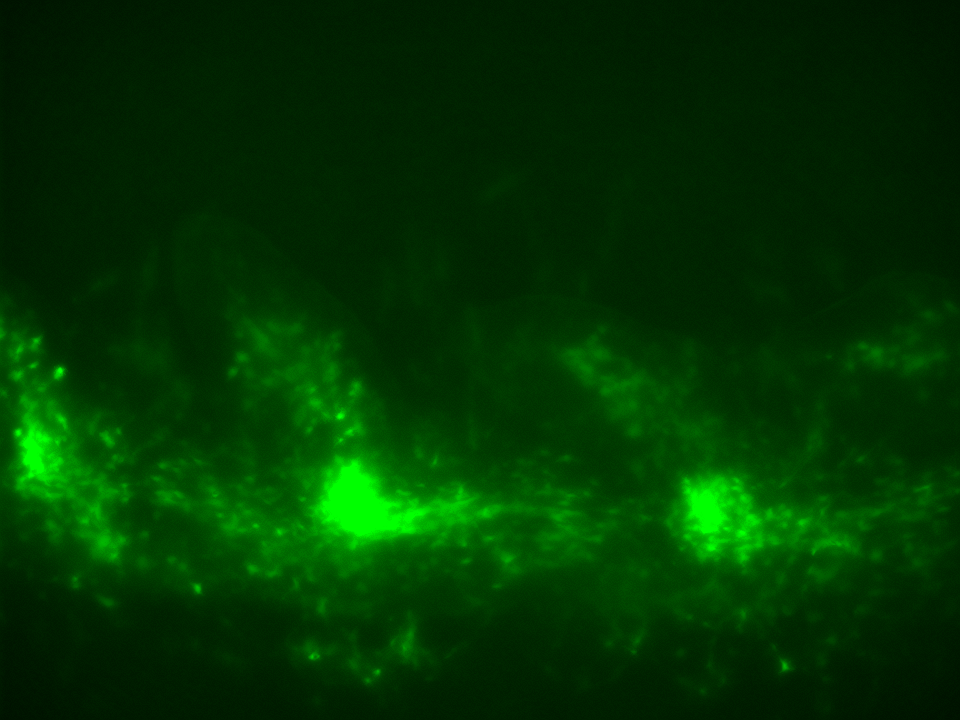

Supplement: Supplementary file 5 — Movie EV3 [file 44318_2026_771_MOESM5_ESM.zip › Fig 3/Fig 3A 36h-2.tif]
