# Supplementary figures and images for: Novel tissue mechanics-guided cellular flows drive the formation of feather follicles (part 2 of 2)
Source: EMBO J. 2026 May 2;45(11):3926–53. doi: 10.1038/s44318-026-00771-7 (PMC13226717; doi:10.1038/s44318-026-00771-7)

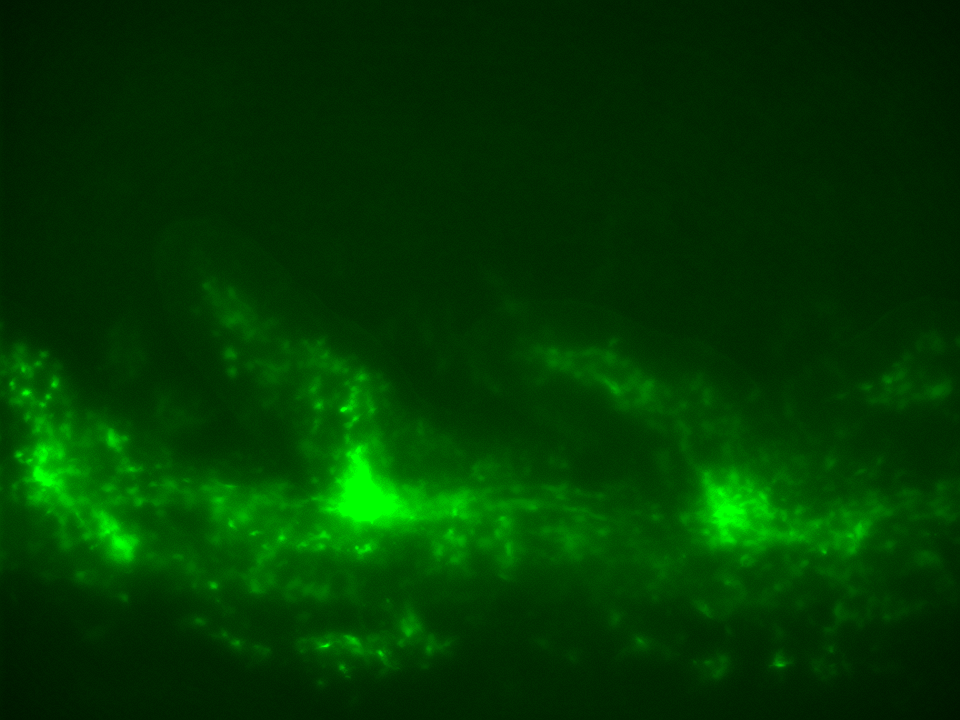

Supplement: Supplementary file 5 — Movie EV3 [file 44318_2026_771_MOESM5_ESM.zip › Fig 3/Fig 3A 36h-3.tif]

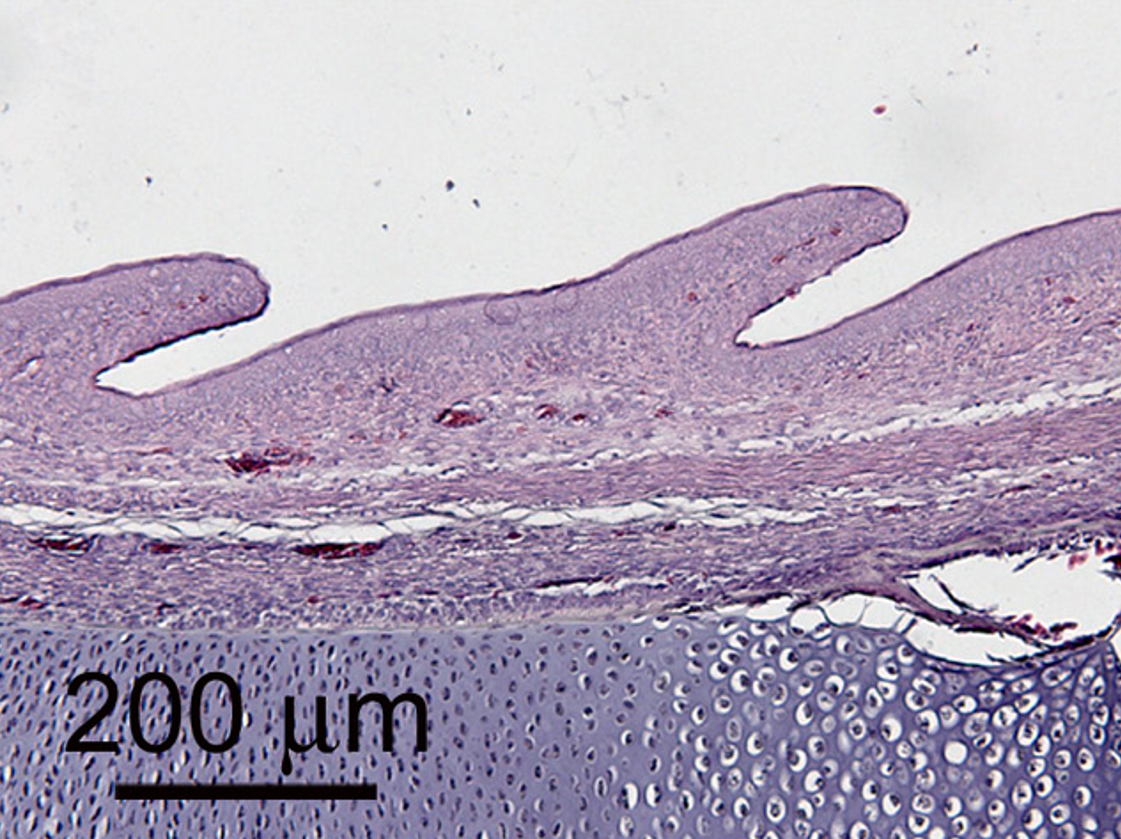

Supplement: Supplementary file 6 — Movie EV4 [file 44318_2026_771_MOESM6_ESM.zip › Fig 5/Fig 5A scale n2.tif]

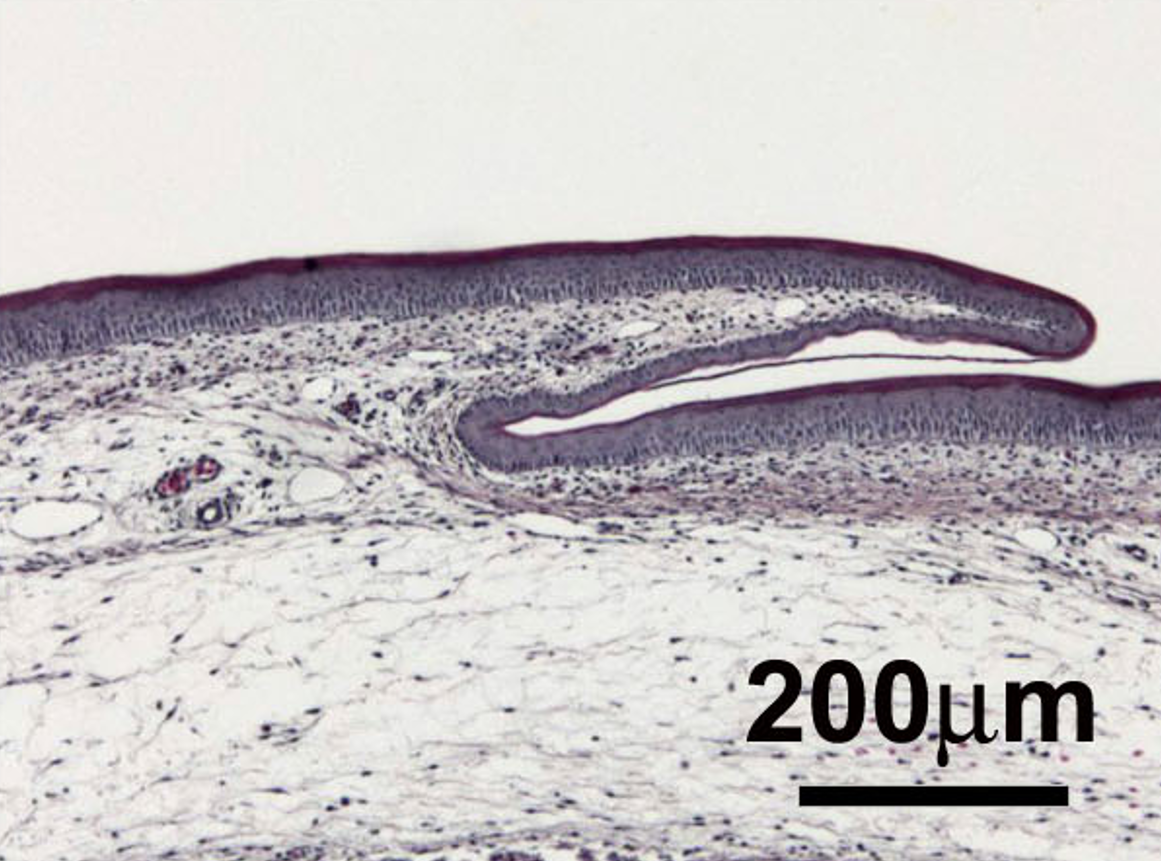

Supplement: Supplementary file 6 — Movie EV4 [file 44318_2026_771_MOESM6_ESM.zip › Fig 5/Fig 5A scale n1.tif]

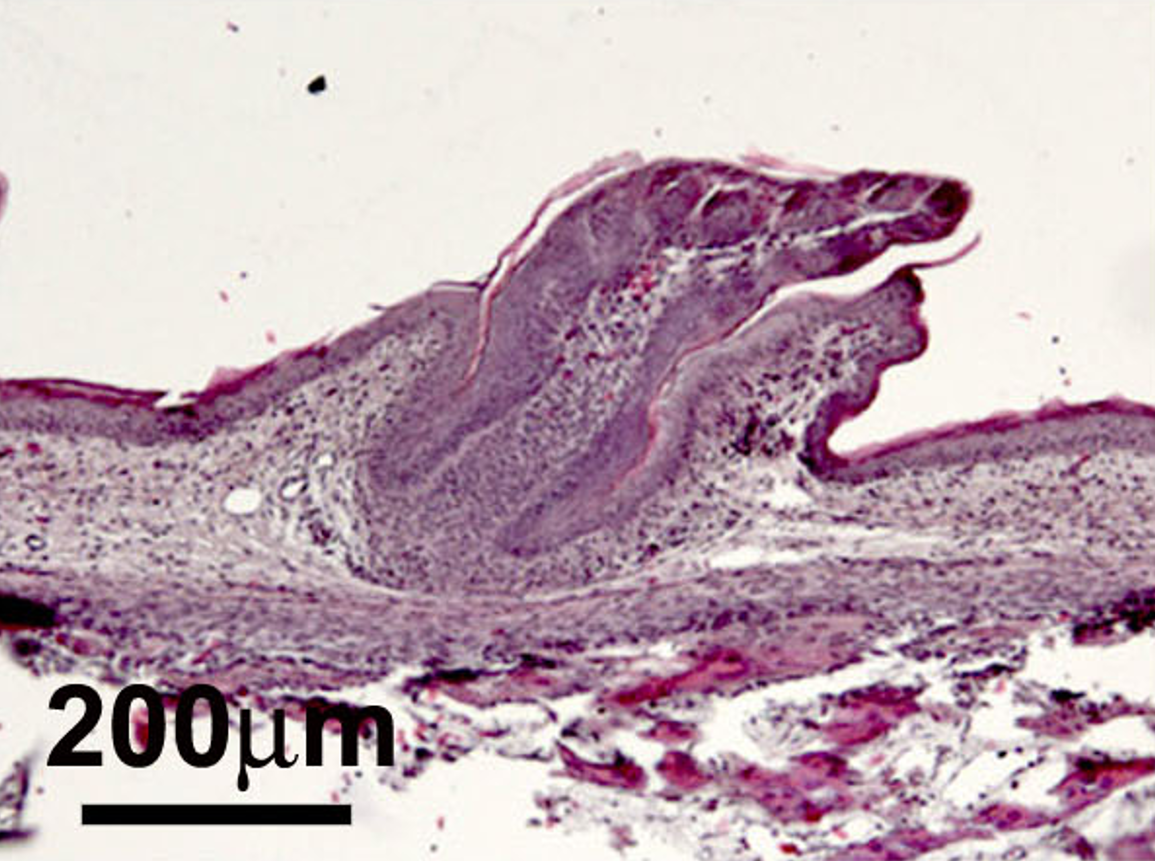

Supplement: Supplementary file 6 — Movie EV4 [file 44318_2026_771_MOESM6_ESM.zip › Fig 5/Fig 5G S2F n2.tif]

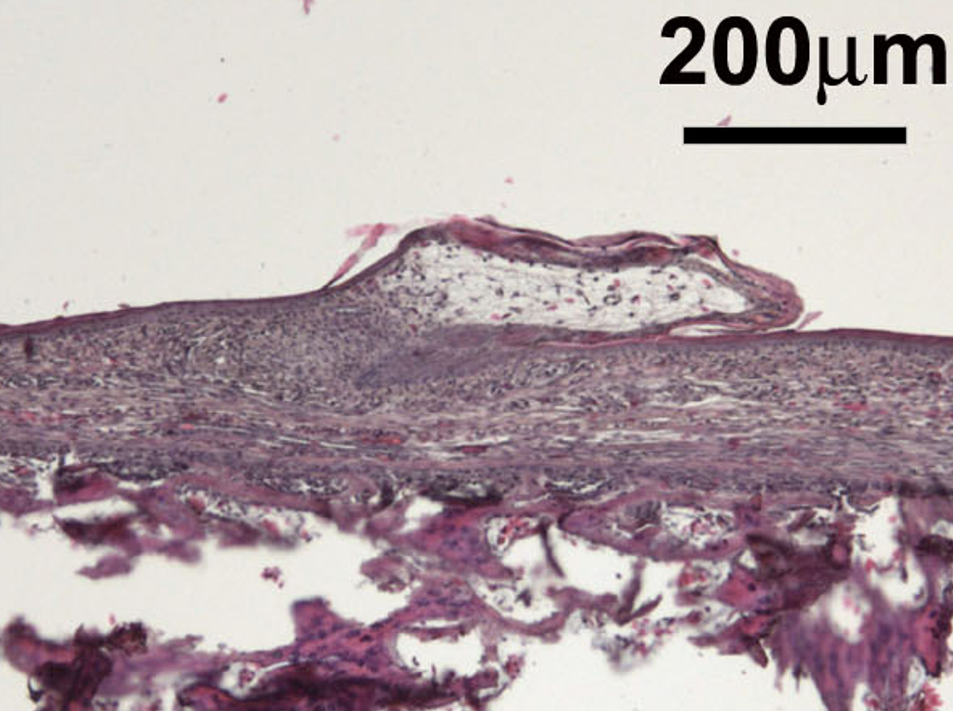

Supplement: Supplementary file 6 — Movie EV4 [file 44318_2026_771_MOESM6_ESM.zip › Fig 5/Fig 5G S2F n1.tif]

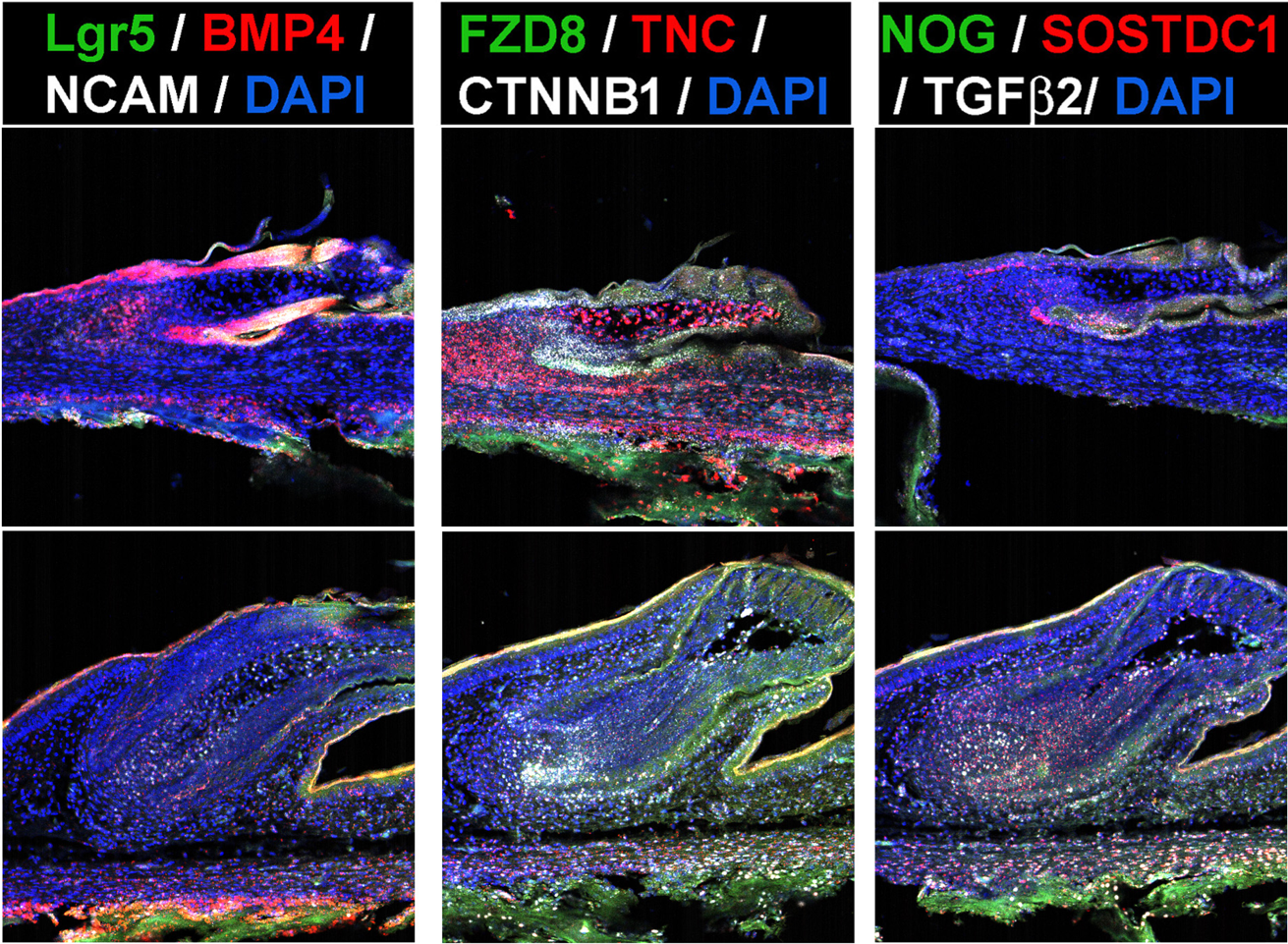

Supplement: Supplementary file 6 — Movie EV4 [file 44318_2026_771_MOESM6_ESM.zip › Fig 5/Fig 5G S2F RNAscope n1-2.png]

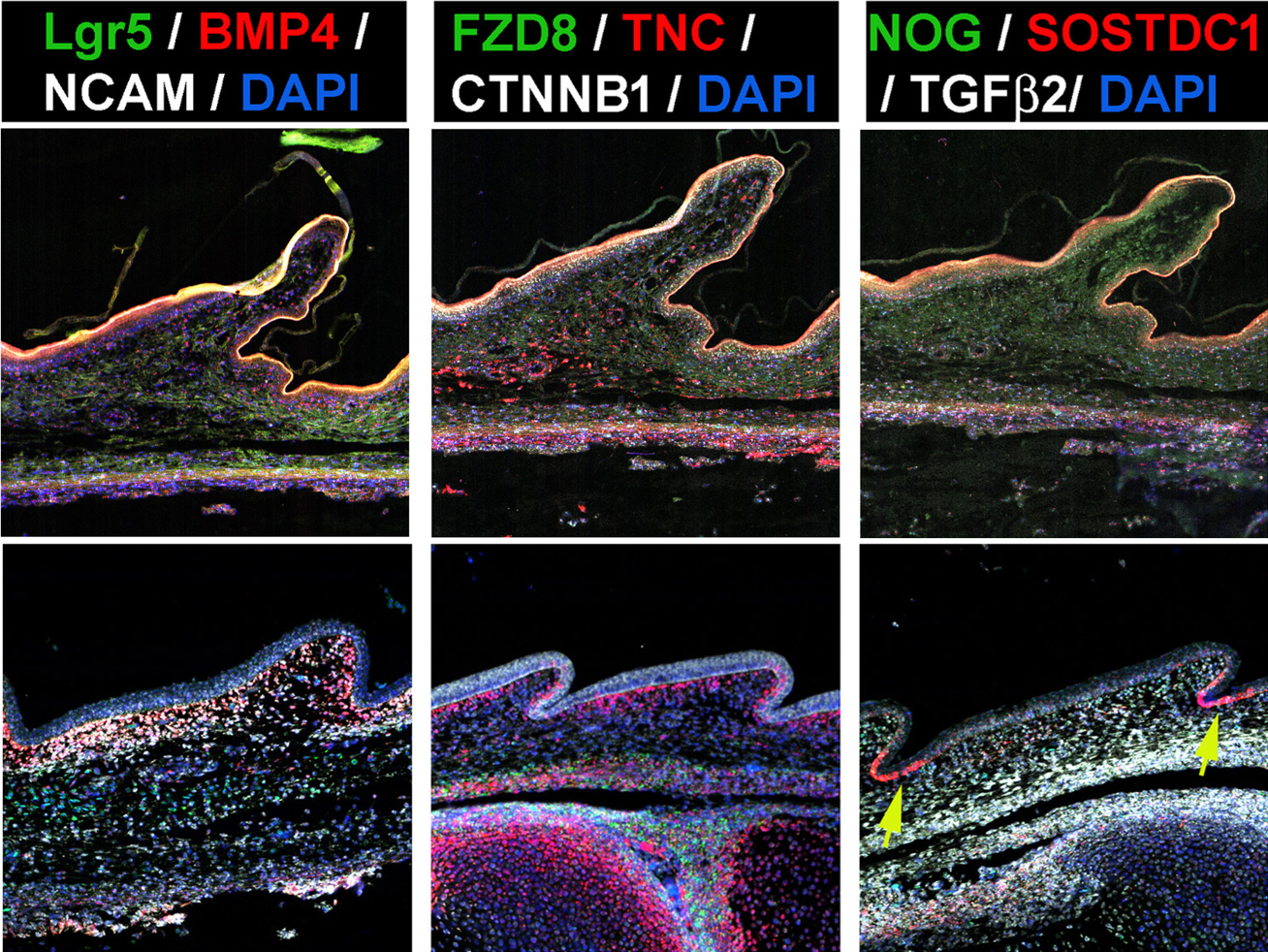

Supplement: Supplementary file 6 — Movie EV4 [file 44318_2026_771_MOESM6_ESM.zip › Fig 5/Fig 5E n1-2.tif]

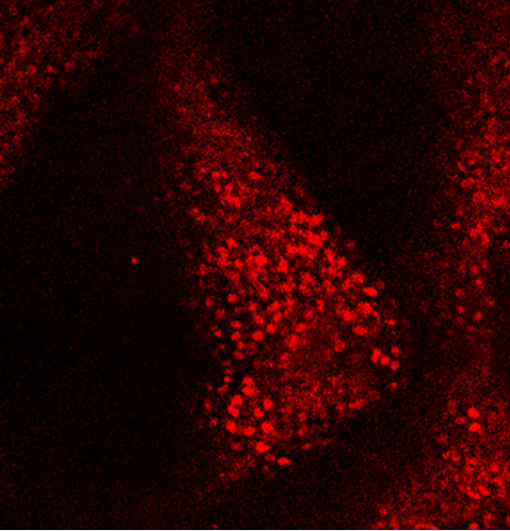

Supplement: Supplementary file 6 — Movie EV4 [file 44318_2026_771_MOESM6_ESM.zip › Fig 5/Fig 5H S2F n1.tif]

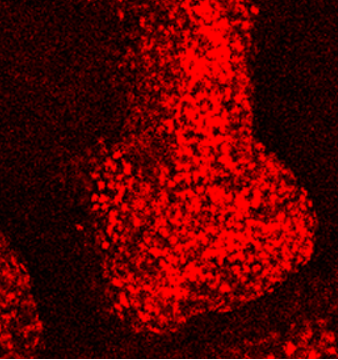

Supplement: Supplementary file 6 — Movie EV4 [file 44318_2026_771_MOESM6_ESM.zip › Fig 5/Fig 5H S2F n3.tif]

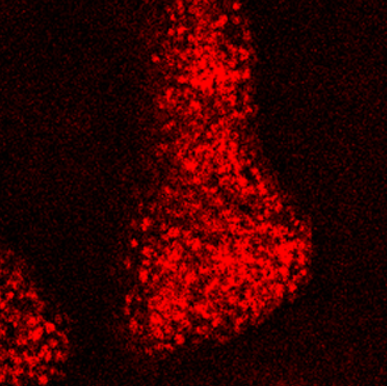

Supplement: Supplementary file 6 — Movie EV4 [file 44318_2026_771_MOESM6_ESM.zip › Fig 5/Fig 5H S2F n2.tif]

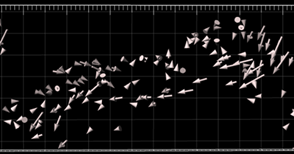

Supplement: Supplementary file 6 — Movie EV4 [file 44318_2026_771_MOESM6_ESM.zip › Fig 5/Fig 5B n2.tif]

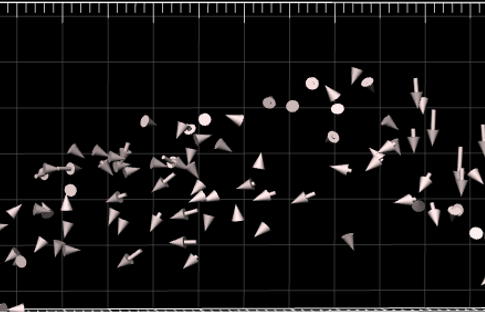

Supplement: Supplementary file 6 — Movie EV4 [file 44318_2026_771_MOESM6_ESM.zip › Fig 5/Fig 5B n3.tif]

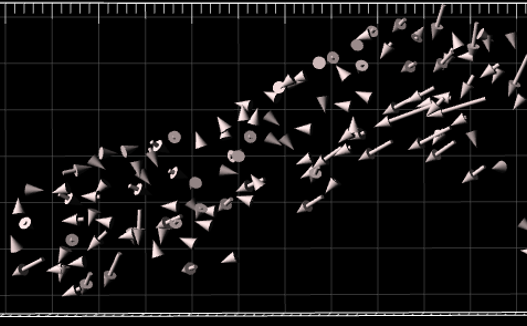

Supplement: Supplementary file 6 — Movie EV4 [file 44318_2026_771_MOESM6_ESM.zip › Fig 5/Fig 5B n1.tif]

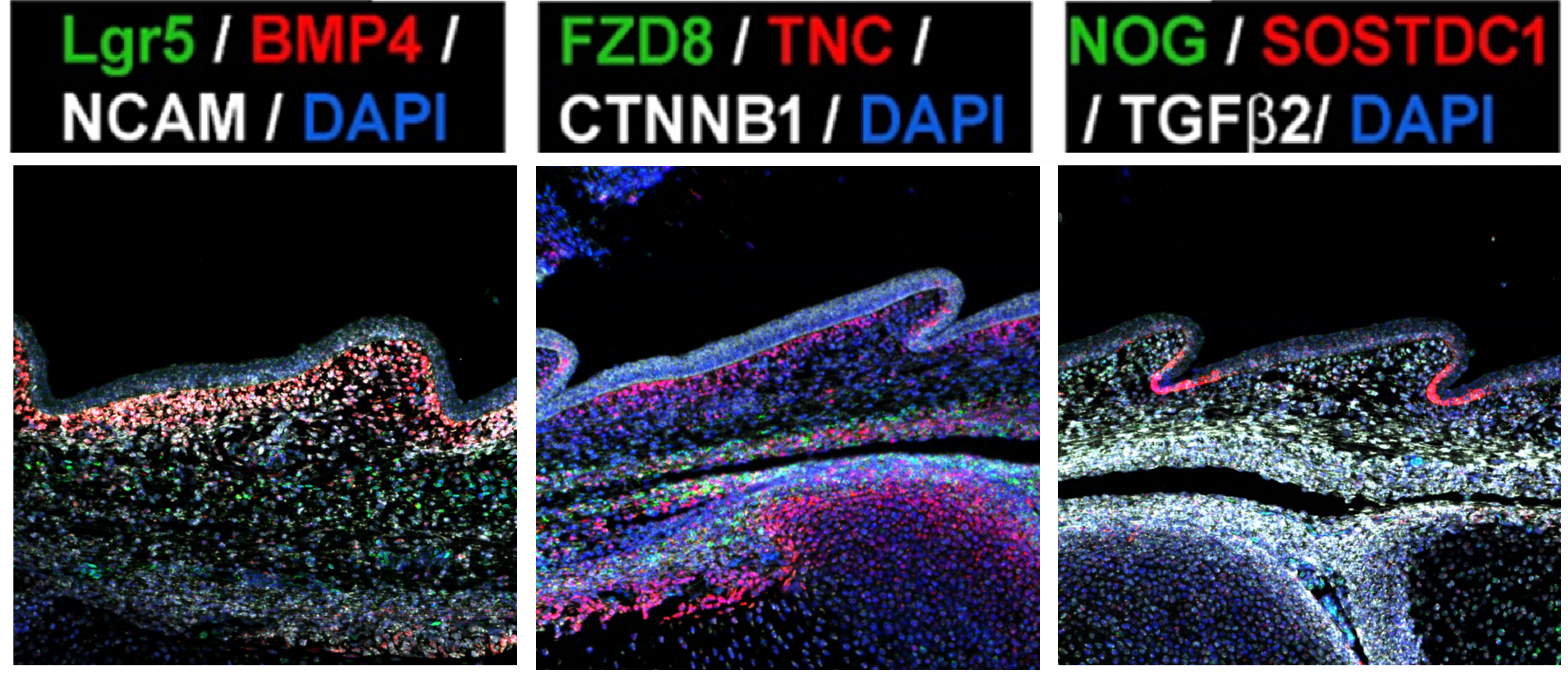

Supplement: Supplementary file 6 — Movie EV4 [file 44318_2026_771_MOESM6_ESM.zip › Fig 5/Fig 5E n3.tiff]

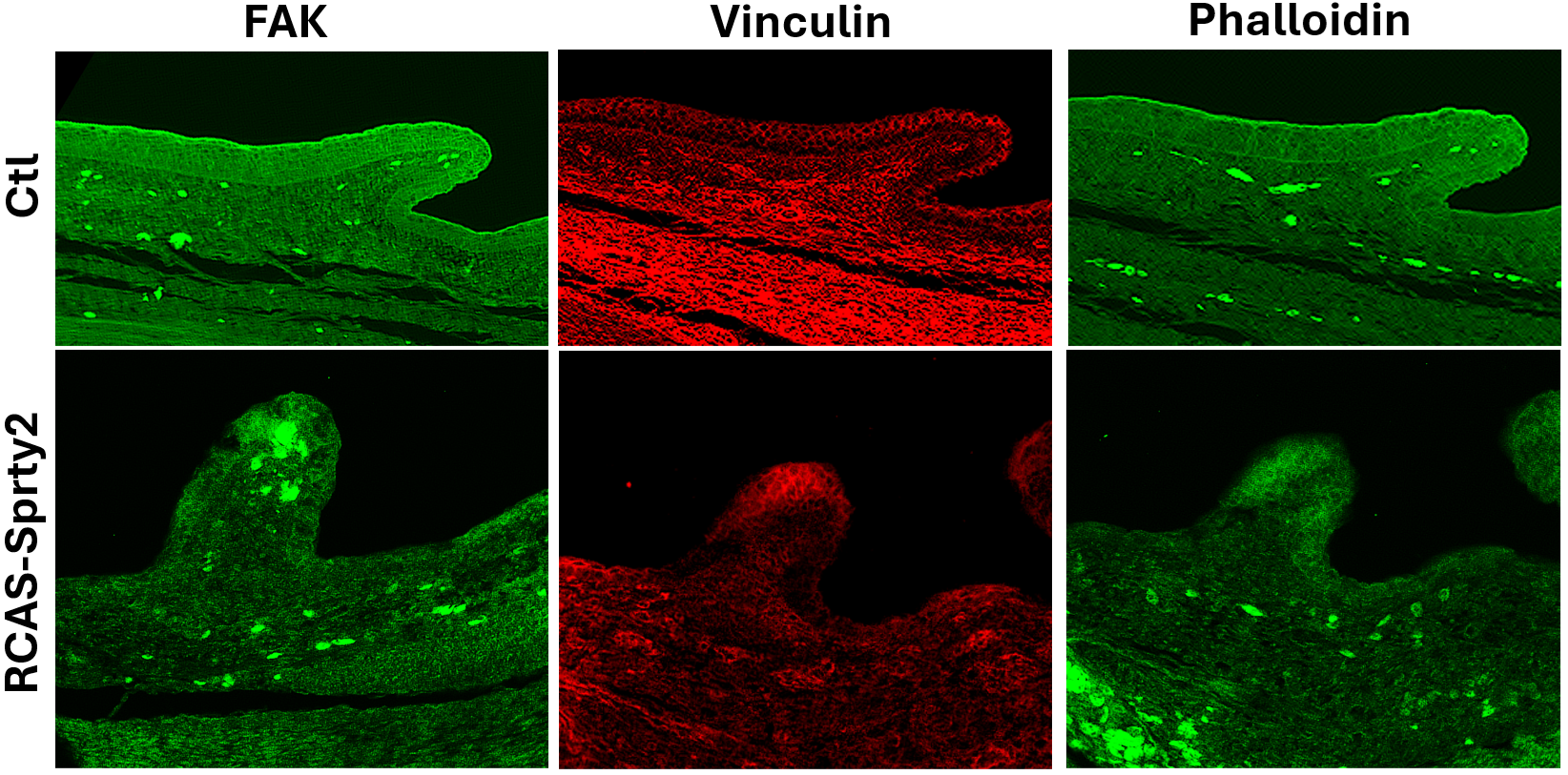

Supplement: Supplementary file 6 — Movie EV4 [file 44318_2026_771_MOESM6_ESM.zip › Fig 5/Fig 5L S2F n3.tif]

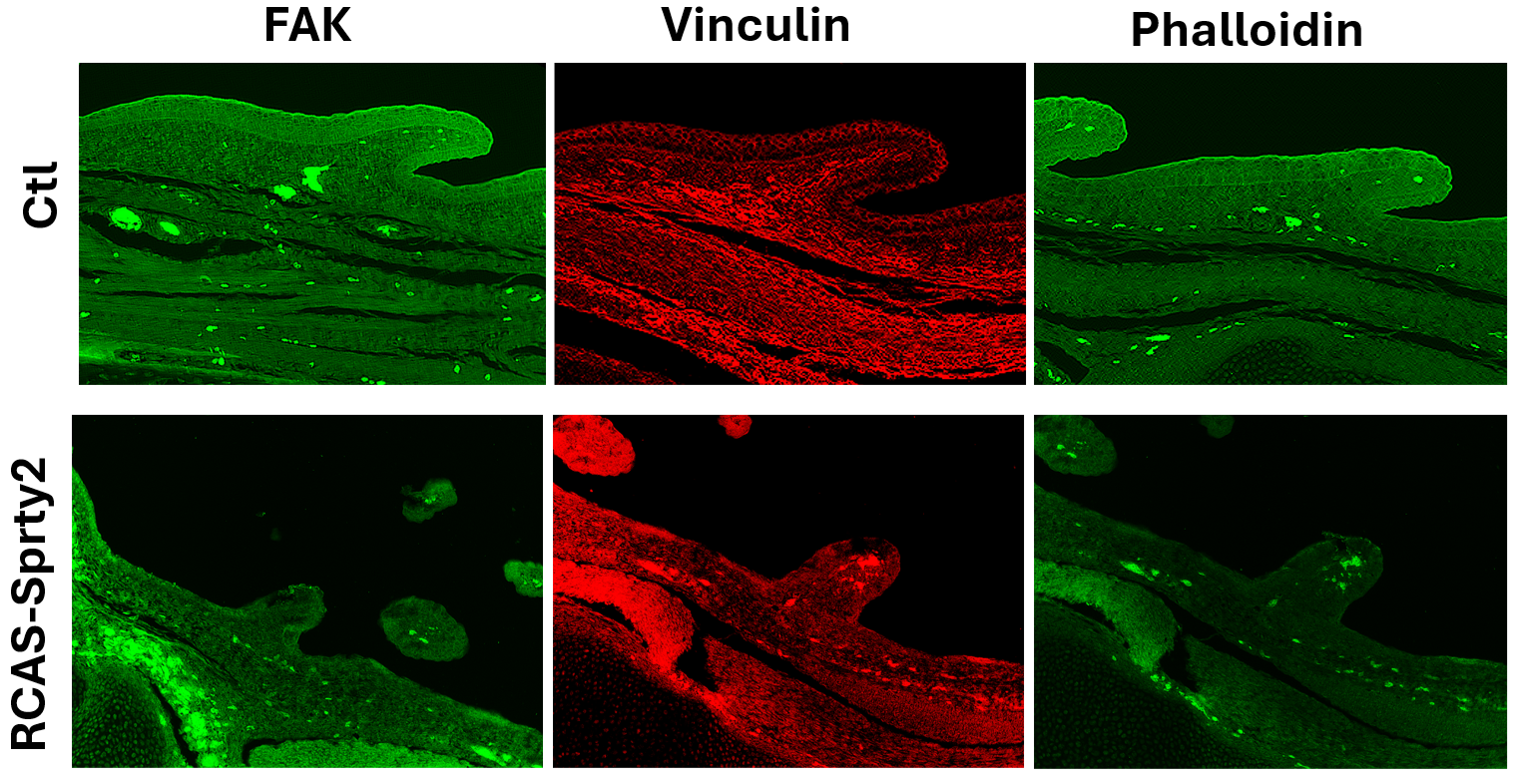

Supplement: Supplementary file 6 — Movie EV4 [file 44318_2026_771_MOESM6_ESM.zip › Fig 5/Fig 5L S2F n2.tif]

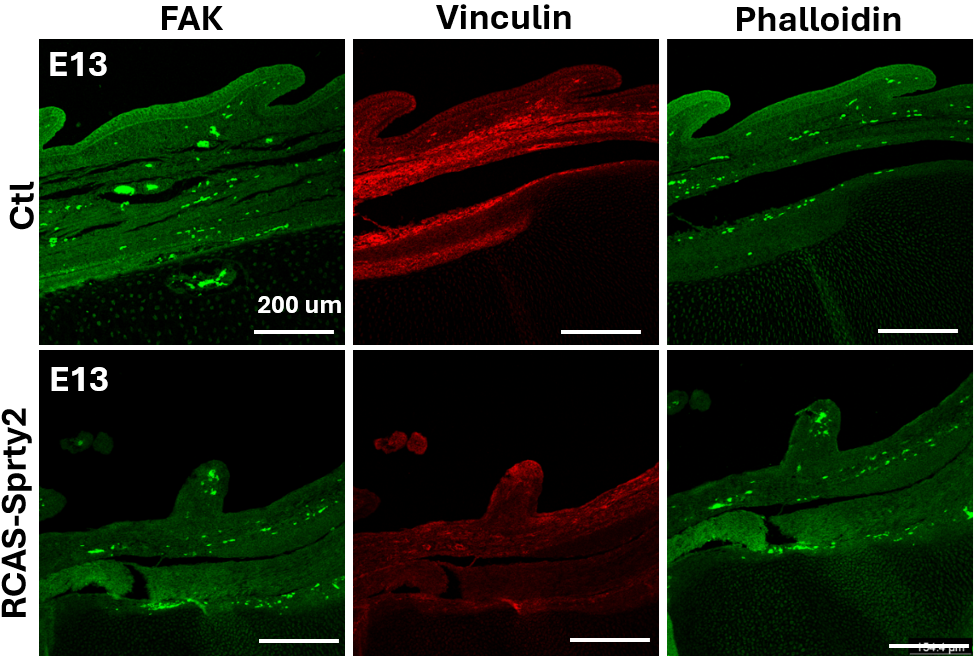

Supplement: Supplementary file 6 — Movie EV4 [file 44318_2026_771_MOESM6_ESM.zip › Fig 5/Fig 5L S2F n1.tif]

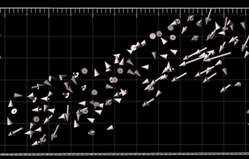

Supplement: Supplementary file 6 — Movie EV4 [file 44318_2026_771_MOESM6_ESM.zip › Fig 5/Fig 5B n1.png]
