# Supplementary material for: Novel tissue mechanics-guided cellular flows drive the formation of feather follicles
Source: EMBO J. 2026 May 2;45(11):3926–53. doi: 10.1038/s44318-026-00771-7 (PMC13226717; doi:10.1038/s44318-026-00771-7)
Supplement: Supplementary file 7 — Movie EV5 [file 44318_2026_771_MOESM7_ESM.zip › Movie EV1.docx]

**Movie EV1. Feather bud protrusion.** Cell tracking video of E7 chicken explant culture for 18h reveals cell migration pattern changed from horizontal to vertical during bud protrusion. Each colour ball represents 1 cell particle tracked using Imaris. The cell tracks (dragon tail) represent the migration pattern of the cell particle throughout the video. The colour gradient of the tracks indicates the timing of the tracks.
