# Supplementary material for: Novel tissue mechanics-guided cellular flows drive the formation of feather follicles
Source: EMBO J. 2026 May 2;45(11):3926–53. doi: 10.1038/s44318-026-00771-7 (PMC13226717; doi:10.1038/s44318-026-00771-7)
Supplement: Supplementary file 8 — Source data Fig. 1 [file 44318_2026_771_MOESM8_ESM.zip › Movie EV2.docx]

**Movie EV2. Feather follicle invagination.** Cell tracking video of E10+24h explant showing different cellular flows. Each colour ball represents 1 cell particle tracked using Imaris. The colour gradient of the tracks (dragon tail) is limited to the last 3h.
