# Supplementary material for: Novel tissue mechanics-guided cellular flows drive the formation of feather follicles
Source: EMBO J. 2026 May 2;45(11):3926–53. doi: 10.1038/s44318-026-00771-7 (PMC13226717; doi:10.1038/s44318-026-00771-7)
Supplement: Supplementary file 9 — Source data Fig. 2 [file 44318_2026_771_MOESM9_ESM.zip › Movie EV3.docx]

**Movie EV3. Dermal papillae formation.** Cell tracking video of E17+24h flight feather to reveal differential cellular flows: The particles are post-edited labelled in different colours according to their position and cell types. (green) DP forming cells migrate downwards. (red) Epidermal tongue cells migrate downwards while distal feather epidermal cells migrate upwards. (yellow) Feather elongating dermal cells migrate distally. Only the last 3h of the track (dragon tail) were shown.
