# Supplementary material for: Novel tissue mechanics-guided cellular flows drive the formation of feather follicles
Source: EMBO J. 2026 May 2;45(11):3926–53. doi: 10.1038/s44318-026-00771-7 (PMC13226717; doi:10.1038/s44318-026-00771-7)
Supplement: Supplementary file 10 — Source data Fig. 3 [file 44318_2026_771_MOESM10_ESM.zip › Movie EV4.docx]

**Movie EV4. Scutate scale formation.** Cell tracking video of E10+24h quail scale showing limited dermal cell migration during scale formation. Epidermal cells are labelled blue while dermal cells are purple. The entire duration of the cell tracks were shown in colour gradient.
