# Supplementary material for: Novel tissue mechanics-guided cellular flows drive the formation of feather follicles
Source: EMBO J. 2026 May 2;45(11):3926–53. doi: 10.1038/s44318-026-00771-7 (PMC13226717; doi:10.1038/s44318-026-00771-7)
Supplement: Supplementary file 11 — Source data Fig. 5 [file 44318_2026_771_MOESM11_ESM.zip › Movie EV5.docx]

**Movie EV5. Scale to feather conversion.** Cell tracking video of a newly induced feather bud on top of the scutate scale. The tracked particle cells are labelled by different colours according to their cell type and location. Purple: scale epidermal cells. Yellow: converted-feather epidermal cells. Blue: scale dermal cells. Black: converted-feather dermal cells. Total duration: 16h, only the last 3h of the track (dragon tail) were shown.
