# Supplementary material for: Novel tissue mechanics-guided cellular flows drive the formation of feather follicles
Source: EMBO J. 2026 May 2;45(11):3926–53. doi: 10.1038/s44318-026-00771-7 (PMC13226717; doi:10.1038/s44318-026-00771-7)
Supplement: Supplementary file 12 — Expanded View Figures [file 44318_2026_771_MOESM12_ESM.pdf]

Expanded View Figures

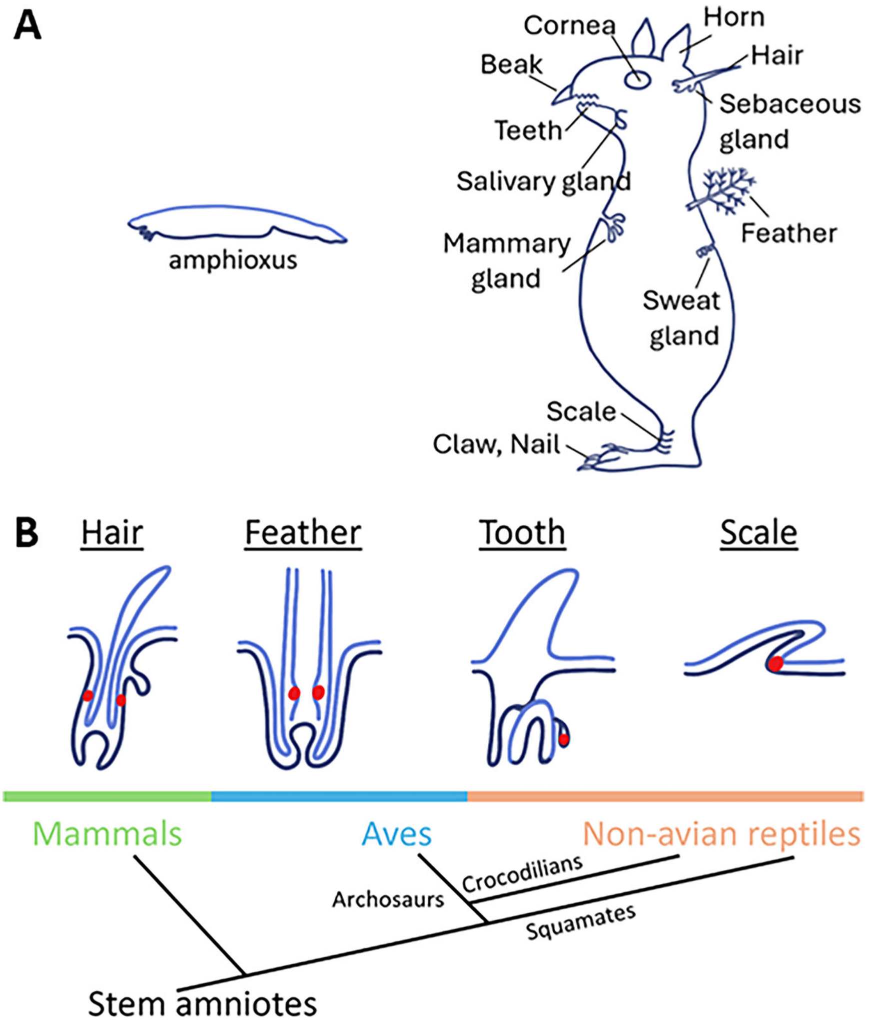

Figure EV1. Evo-devo of integumentary organs.

(**A**) Ancestral chordates such as amphioxus have a smooth integument. As vertebrates evolve, different types of skin appendages emerge to help animals interact with the environment. Shown here is a conceptual animal with different integumentary organs. Modified from Chuong CM edit, 1998. Molecular Basis of Epithelial Appendage Morphogenesis. Landes Biosciences. (**B**) Among these integumentary appendages, the follicle architecture provides epidermal stem cells (red dots) with a dermal niche, facilitating the molting of the distal differentiated structures and renewal of new appendages. Follicles in hair, feathers and reptile teeth result from convergent evolution. Scales do not have this configuration and keep homeostasis similar to the mechanism used in the epidermis.

 © The Author(s)

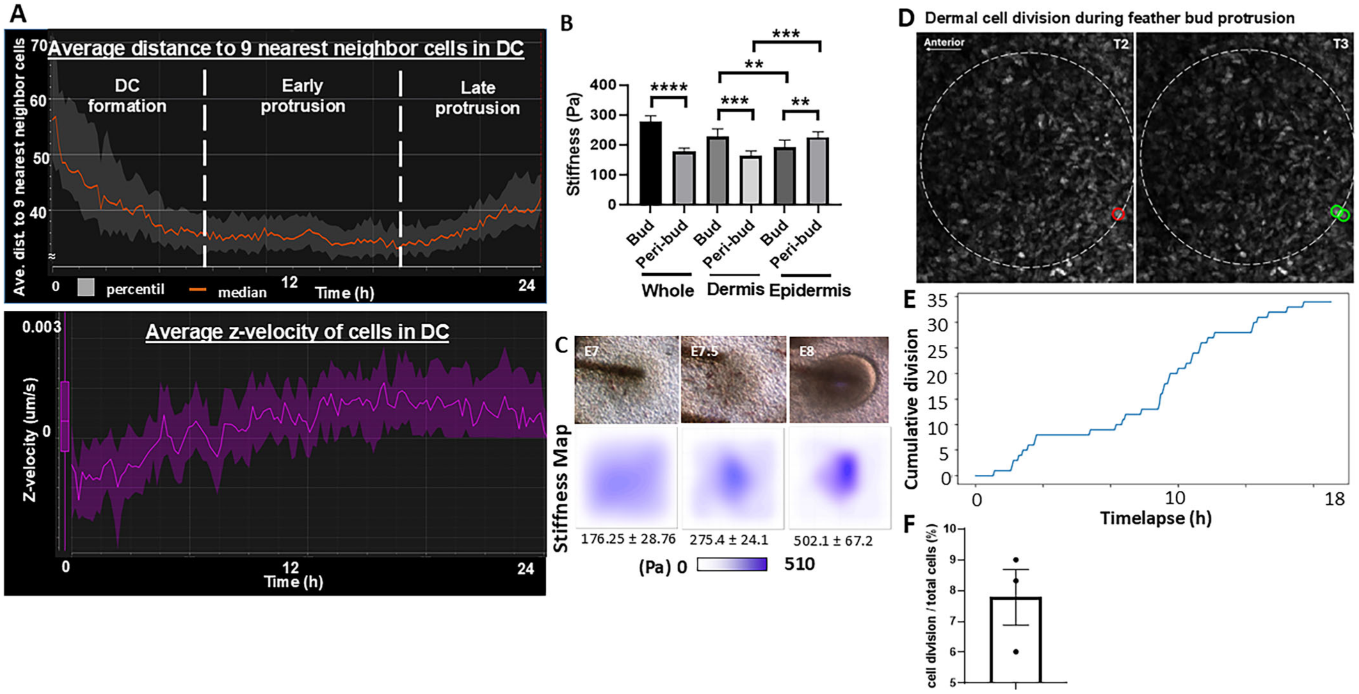

Figure EV2. Biophysical characterization of feather bud formation.

(**A**) Top panel, average distance to nine nearest neighbor cells. Lower panel, average z-velocity in DC during feather bud protrusion. Representative results. $n = 3/3$, biological replicates. (**B**) Average tissue stiffness of whole, dermis and epidermis of bud and peribud regions of E8 skin. Two-way ANOVA, Whole, bud vs peribud: $F(1, 8) = 304.7$ ****, <0.0001. Dermis, bud vs peribud: $F(1, 8) = 35.79$ ***$P = 0.0003$. Epidermis, bud vs peribud: $F(1, 8) = 37.66$ **$P = 0.0031$. Dermis bud vs epi bud, $F(1, 8) = 11.47$, **$P = 0.0095$. Dermis peribud vs epidermis peribud: $F(1, 8) = 45.76$, ***$P = 0.0001$. $n = 9/5$. Nine measurements from five biological replicates. (**C**) Stiffness map of E7, E7.5, and E8 feather buds showing during dermal condensation and feather bud protrusion, the regional stiffness gradually increases from 176 ± 28 Pa to 275 ± 24 and 502 ± 67 Pa, respectively. $n = 3/3$. (**D**) Representative photos of detected cell division at the posterior end of the feather bud during its protrusion at T2 and T3 of E8 + 18 h video. The arrow indicates the anterior end of the feather bud. $n = 3/3$, biological replicates. (**E**) Representative graph of cumulative cell division events observed in 511 dermal nuclei within the feather bud. $n = 3/3$, biological replicates. (**F**) Average percentage of cell division events over total cells in three E8 + 18 h feather buds. $N = 3/3$, biological replicates.

© The Author(s)

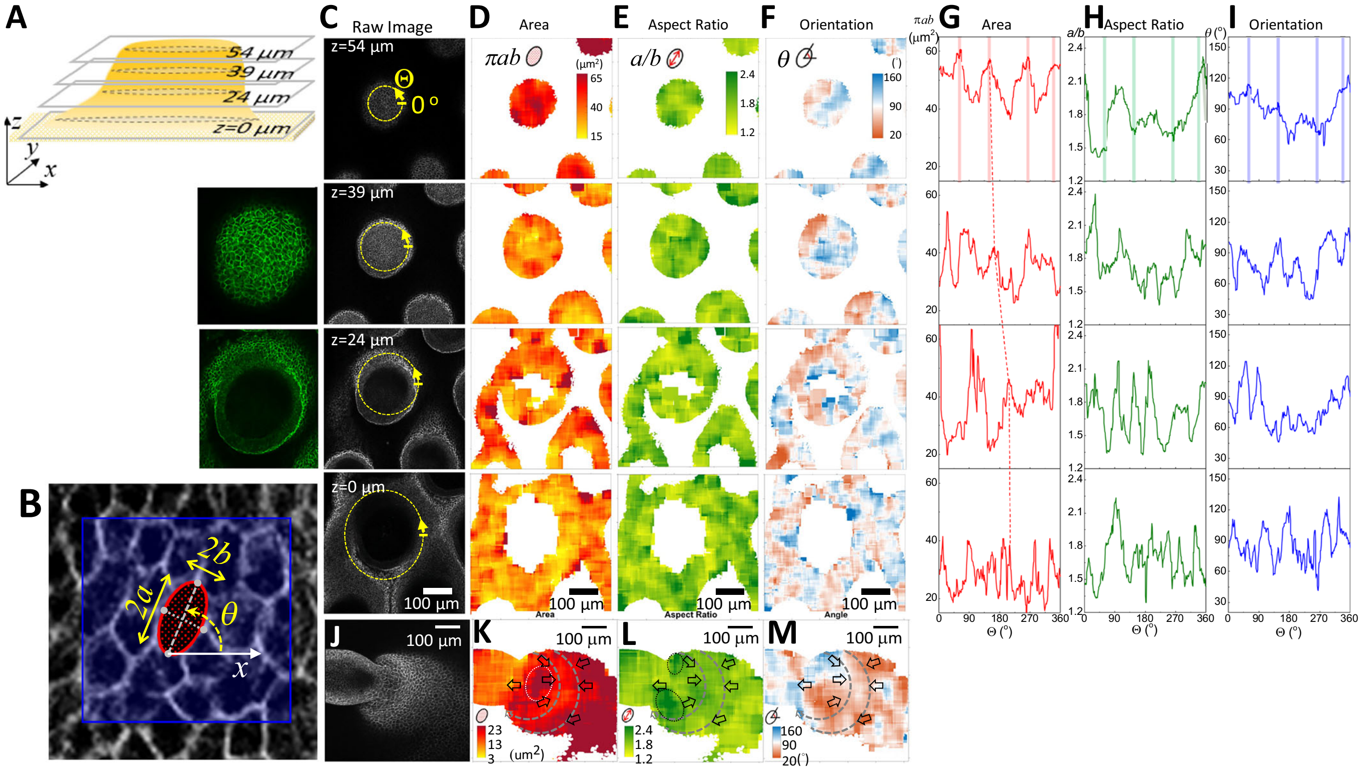

Figure EV3. QMorF (Image-cased quantitative morphology field measurement).

(**A**) Illustration of different section levels of an E9 feather bud that are used for analyses. (**B**) Representative quantifiable parameters of a cross-sectional cellular morphology in the QMorF analysis. $n = 3/3$, biological replicates. (**C**) Raw image of respective sections. The green lines point to the magnified original E-cadherin staining images of respective feather bud z-sections. $n = 3/3$, biological replicates. (**D–F**) Area, aspect ratio, and orientation of respective sections. $n = 3/3$, biological replicates. (**G–I**) Quantified area, aspect ratio, and orientation of cell shape over the azimuthal direction (Θ-axis) along the yellow dot circle in panel (**C**) of respective sections. Four peaks distributed over the azimuthal direction of the area plot in panel (**G**) at the bud tip (z = 54 μm) suggest a fourfold morphological symmetry of cells to accommodate the confined tip geometry. (**J–M**) Whole mount of E10 skin stained with antibodies to L-CAM (E-cadherin). Raw image used for quantification of area, aspect ratio, and orientation of invaginating E10 feather bud. $n = 3/3$, biological replicates. Please see the methods for QMorF analysis.

 © The Author(s)

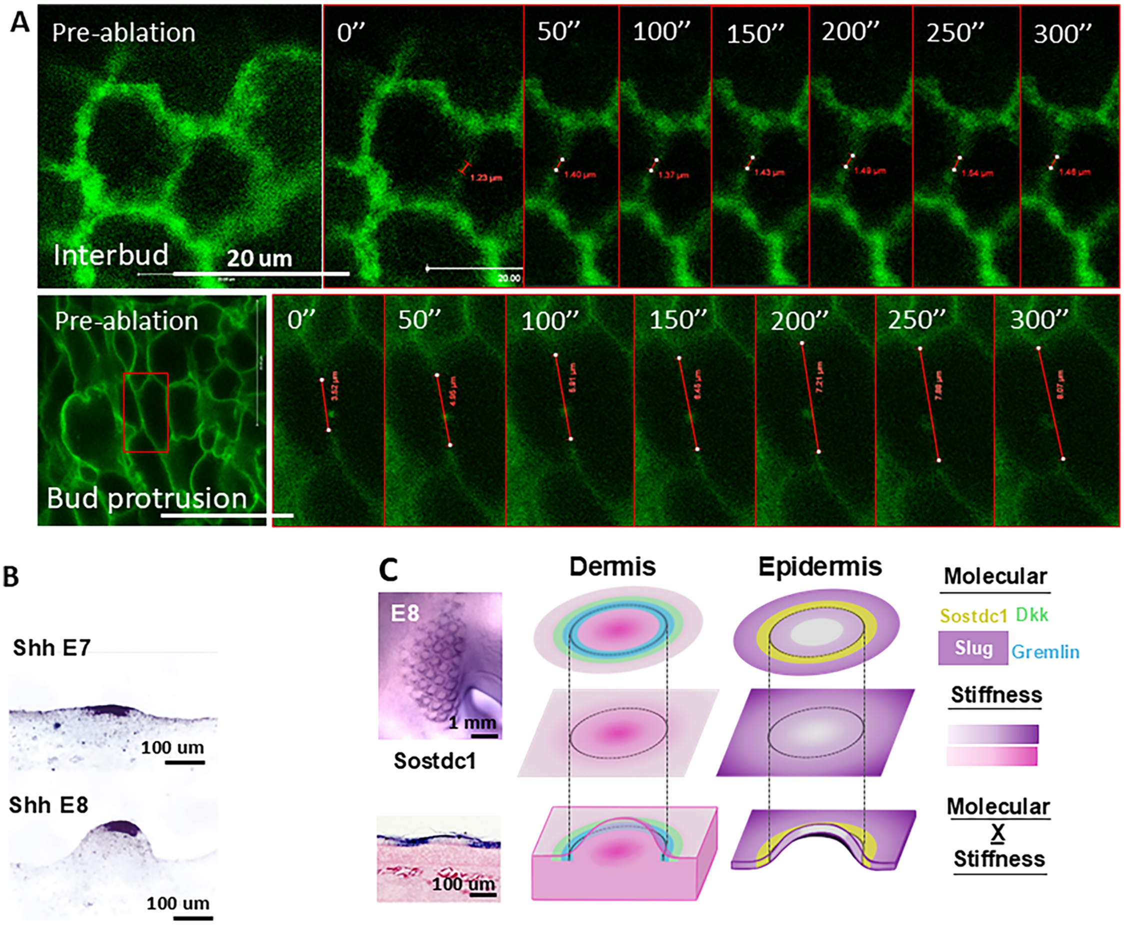

Figure EV4. Cellular tension and molecular expression of protruding feather buds.

(**A**) Laser ablation analyses showing ablated cell membrane retracted faster in epidermal cells of E8 protruding feather buds than in the interbud region. $n = 3/3$, biological replicates. (**B**) In situ hybridization of Shh of E7 and E8 chicken skin. $n = 3/3$, biological replicates. (**C**) Spatial expression pattern of Sostdc1, Slug, DKK1 and Gremlin in conjunction with spatial stiffness in the E9 dermis and epidermis. Notice these molecules demarcate bud boundaries.

© The Author(s)

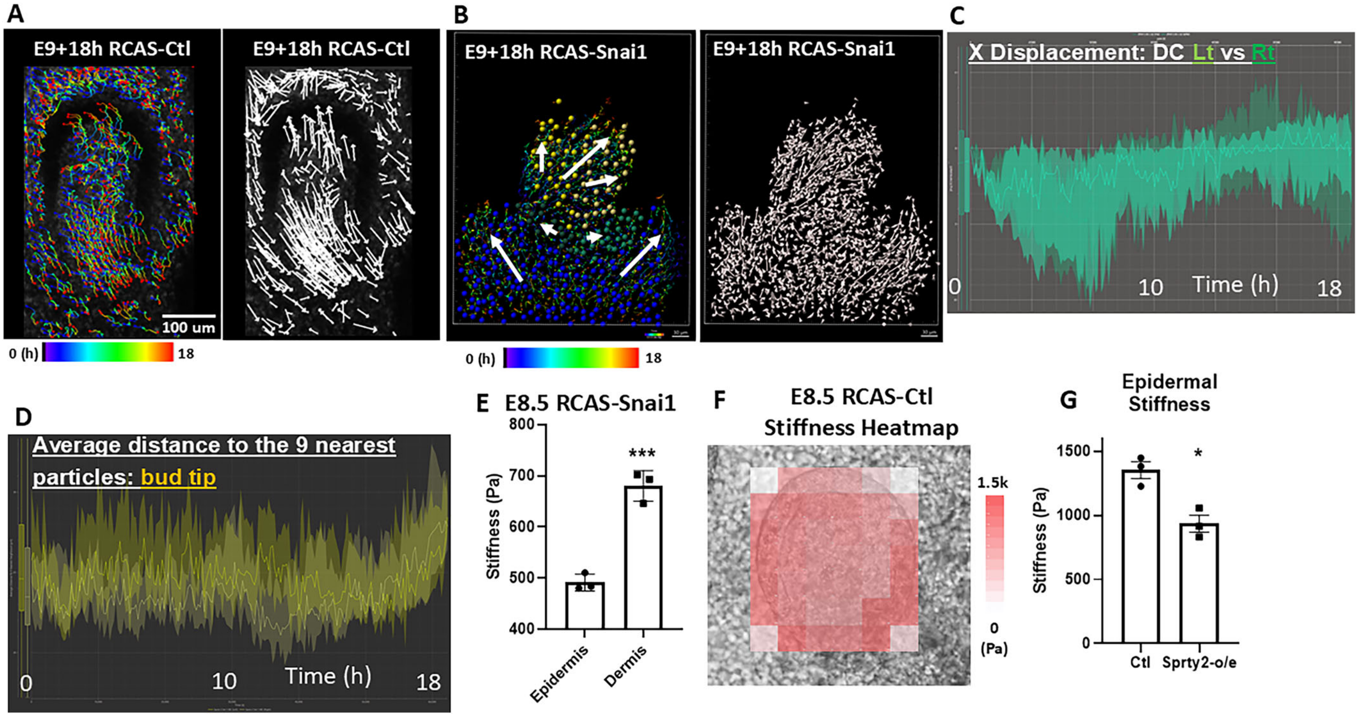

Figure EV5. Dispersed cell migration pattern in the feather bud was demonstrated using Imaris cell track analysis of E9 + 18 h RCAS-Snai1 chicken skin.

(**A**) Cell tracking of E9 + 18 h RCAS-Ctl. Left: Dermal cell tracked and color scale from 0 to 18 h. Right: arrowheads showing the initial to final displacement of dermal cells after 18 h of culturing. *N* = 3/3, biological replicates. (**B**) Cell tracking of E9 + 18 h RCAS-Snai1. Left: dermal cells of different regions labeled with specific colors. Yellow: feather bud dermal cells. Green: dermal condensate cells. Blue: non-feather bud dermal cells. Color scale: 0–18 h. Right: arrowheads showing the initial to final displacement of dermal cells after 18 h of culturing. *n* = 3/3, biological replicates. (**C**) E9 + 18 h RCAS-Snai1. Graph of X-axis (left-right) displacement of dermal condensate cells (DC) in left (Lt) and right (Rt) halves of DC over 18 h. *n* = 3/3, biological replicates. (**D**) E9 + 18 h RCAS-Snai1. The dispersed migrating pattern of feather bud dermal cells is demonstrated by the average distance to the 9 nearest particles in the feather bud dermal cells in the 15–18 h of cell tracking. Representative result. *n* = 3/3, biological replicates. (**E**) Stiffness comparison of E8.5 RCAS-Snai1 epidermis vs dermis. *T*-test, ****P* = 0.0007, *n* = 3/3, biological replicates. (**F**) Average stiffness heatmap of E8.5 RCAS-Ctl feather bud. *n* = 3/3, biological replicates. (**G**) Epidermal stiffness comparison of E8.5 RCAS-Ctl vs Sprty2 overexpression feather bud. *T*-test, **P* = 0.0109, *n* = 3/3, biological replicates.

 © The Author(s)

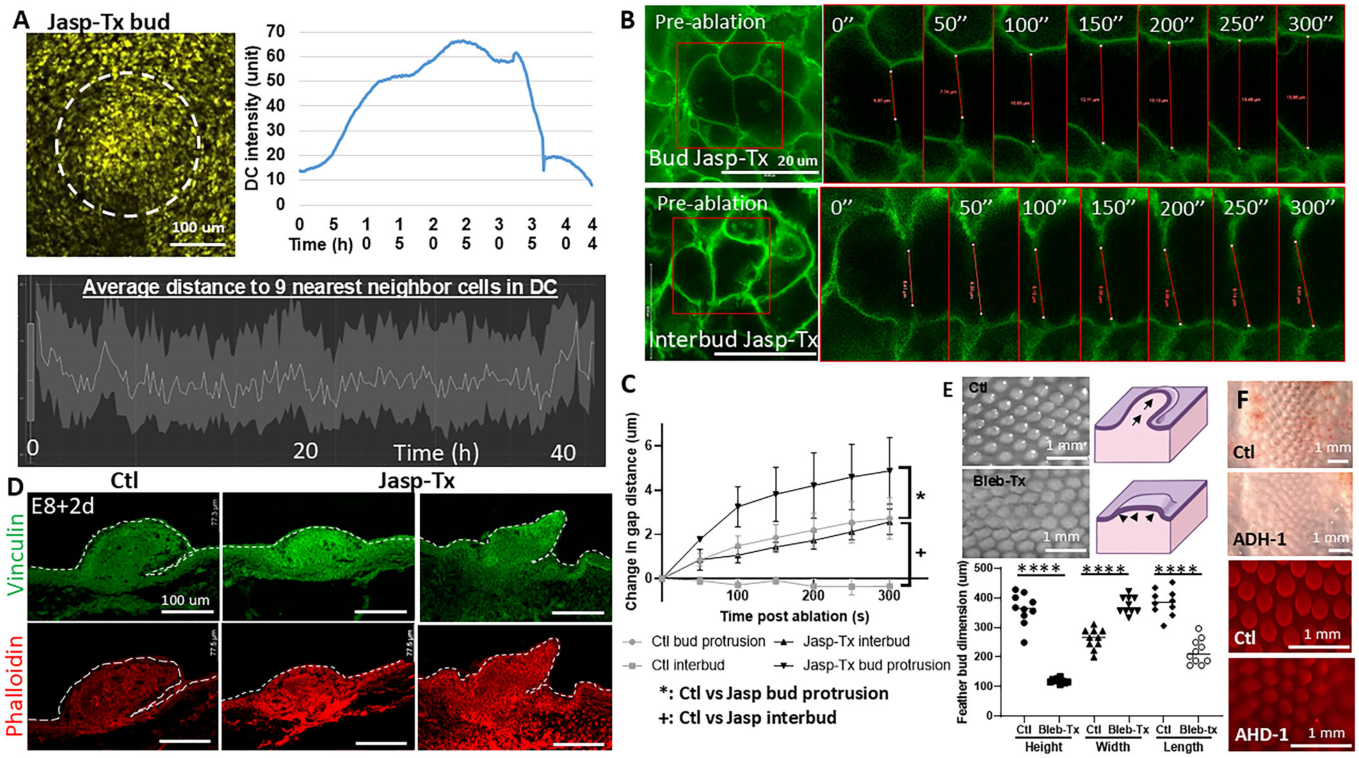

Figure EV6. Perturbing the actomyosin system affects dermal condensation, cellular membrane tension dynamics, and normal feather bud growth.

(**A**) Jasp-Tx E8 feather bud failed to progress after forming the initial dermal condensate (dotted line). Intensity of the DC and average distance to nine nearest neighbor cells in the DC both show an initial formation of DC and then disassemble after 30 h of Jasp-Tx. Representative image, $n = 3/3$, biological replicates. Scale bar: 100 um. (**B**) Laser ablation analyses showing Jasp-Tx increased epidermal cell membrane retraction rate in both bud and interbud region of E8 quail skin. Scale bar: 20 um. Representative results. $n = 3/3$, biological replicates. (**C**) Quantified results of the change in gap distance after laser ablation in various conditions. Two-way ANOVA. Ctl vs Jasp bud protrusion: F (1.177, 4.707) = 12.15, $^*P = 0.0179$. Ctl vs Jasp interbud: F (2.021, 8.084) = 7.817, $+P = 0.0372$. $n = 3/3$, biological replicates. (**D**) Representative results of vinculin and phalloidin (F-actin) detection in E8 + 2 d control and Jasp-Tx quail feather buds. Vinculin is a focal adhesion protein, and phalloidin labels F-actin. Scale bar: 100 um. $n = 3/3$, biological replicates. (**E**) Blebbistatin treatment (Bleb-Tx) led to shortened and widened feather buds in E9 explants cultured for 48 h. Graph: quantified dot graph of feather bud dimension changes with and without Bleb-Tx. Scale bar: 1 mm. *T*-test, $P < 0.0001$ for all three comparisons. $n = 10/3$, biological replicates. (**F**) N-cadherin inhibitor ADH-1 treatment in E8 chicken explants for 2 days impeded normal feather bud development, leading to smaller feather buds. Scale bar: 1 mm. Representative result. $n = 3/3$, biological replicates.

© The Author(s)

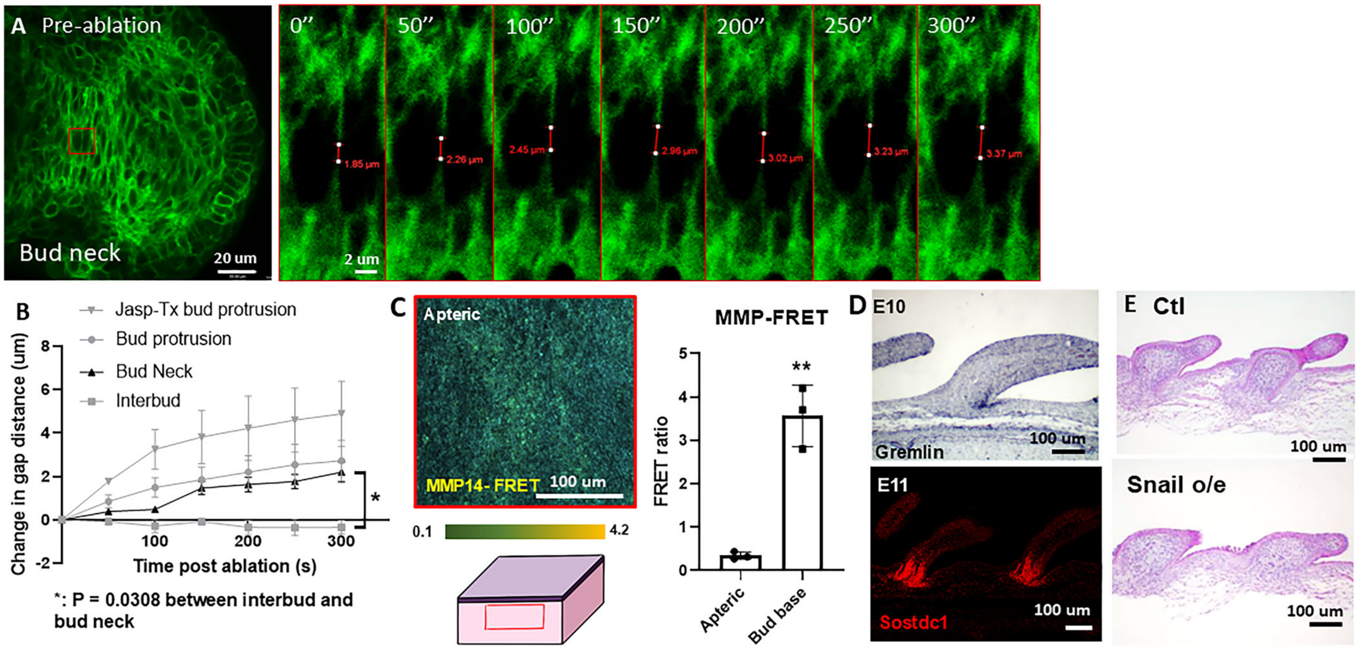

Figure EV7. Molecular expression and cellular dynamics of invaginating feather follicle.

(**A**) Laser ablation analyses showing epidermal cell membrane retraction in the bud neck region of an invaginating feather follicle of E8 quail skin. Scale bar: 20 um. $n = 3/3$, biological replicates. (**B**) Quantified results of the change in gap distance after laser ablation in the bud neck and various other conditions. Two-way ANOVA: Bud neck vs interbud, F (1.422, 5.687) = 7.419, $^*P = 0.0308$. $n = 3/3$, biological replicates. (**C**) MMP14-FRET activity at the apteric region of E11 chicken skin, as illustrated. Graph: MMP14-FRET activity of apteric vs bud base region. Scale bar: 100 um. *t*-test, $P < 0.0015$. $n = 3/3$, biological replicates. (**D**) In situ hybridization of Gremlin in E10 feather bud and Sostdc1 in E11 chicken skin. Scale bar: 100 um. $n = 3/3$, biological replicates. (**E**) H&E photos of Ctl and Snail overexpression in the epidermis inhibited epidermal invagination in E7 reconstituted skin for 7 days. $n = 3/3$, biological replicates.

 © The Author(s)

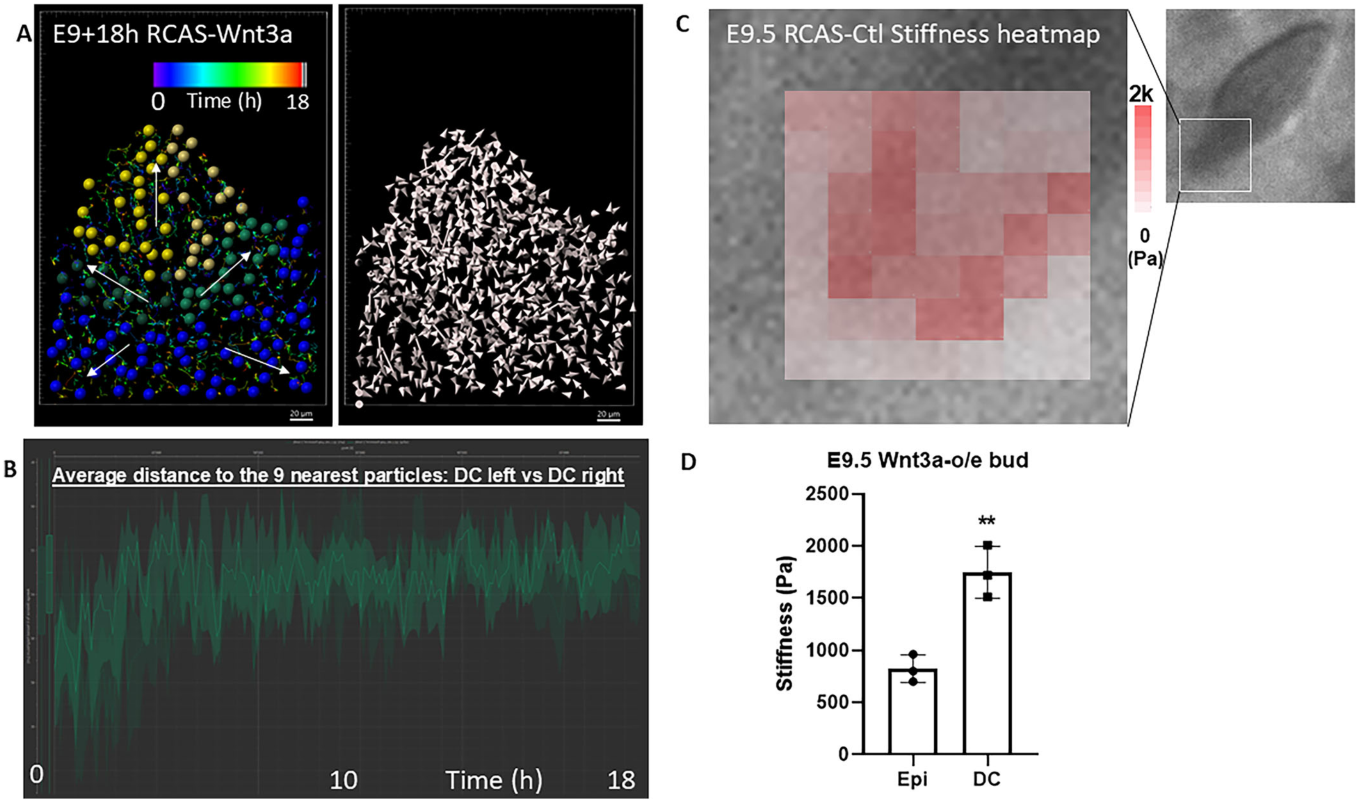

Figure EV8. RCAS-Wnt3a overexpression in the epidermis leads to dispersed dermal condensate cells.

(**A**) Left: dermal cells of different regions labeled with specific colors. Yellow: feather bud dermal cells. Green: dermal condensate cells. Blue: non-feather bud dermal cells. Color scale: 0–18 h. Right: arrowheads showing the initial to final displacement of dermal cells after 18 h of culturing. $n = 3/3$, biological replicates. (**B**) Average distance to the nine nearest particles increased in both DC left and DC right after 3 h of culturing in RCAS-Wnt3a-overexpressing feather follicle. $n = 3/3$, biological replicates. (**C**) Top: average stiffness heatmap of E9.5 RCAS-Ctl bud neck and invagination area. $n = 3/3$, biological replicates. (**D**) Stiffness comparison of E9.5 Wnt3a-o/e bud epidermis vs DC. *T*-test, $P = 0.0048$. $n = 3/3$, biological replicates.

© The Author(s)

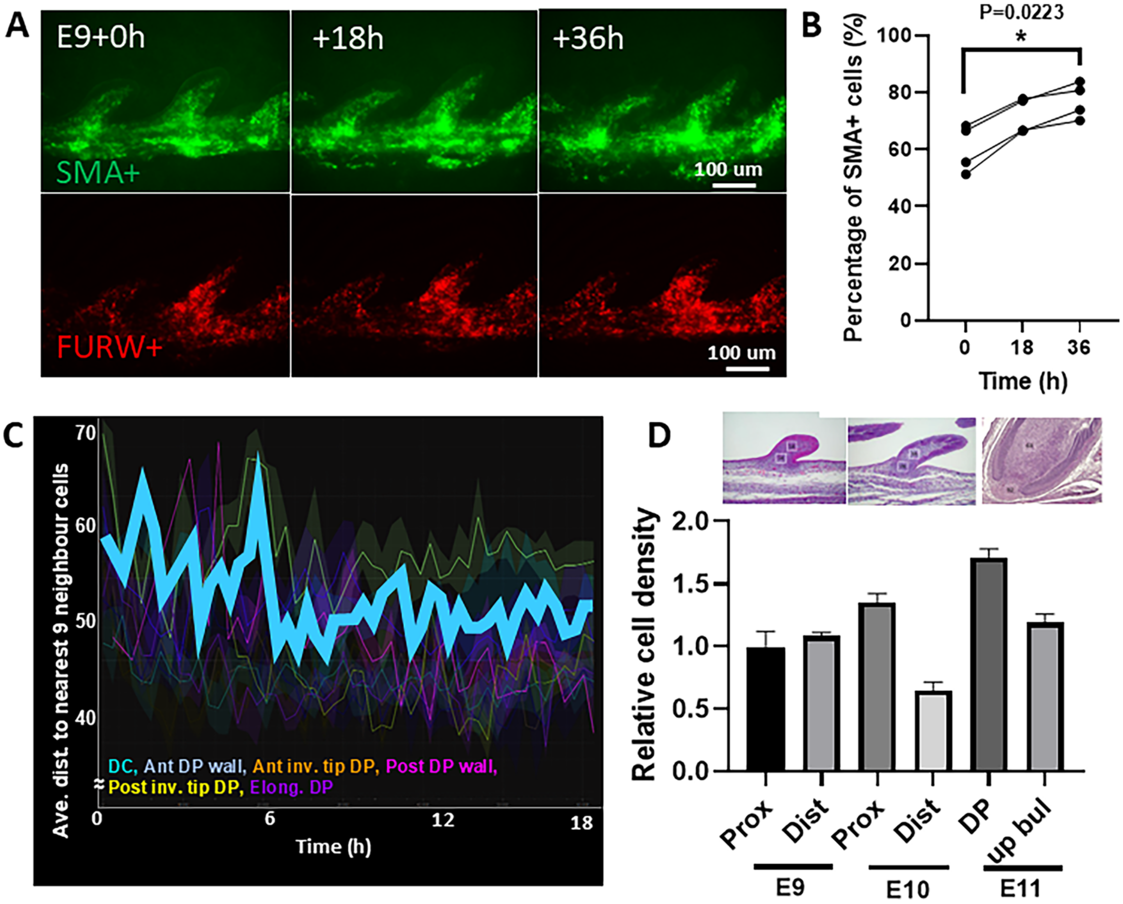

Figure EV9. Molecular expression and cellular dynamics of dermal papillae formation.

(**A**) The changes in SMA+ (green) and overall cell (red, FUKOW+) in growing E9 explant for 36 h. $n = 4/4$, biological replicates. (**B**) Graph showing quantified percentage of SMA+ over FUKOW+ cells in 4 E9 explant feather follicles. One-way ANOVA post hoc Tukey's. $F = 5.974$, 0 vs 36 h, $p = 0.0223$. $n = 4/4$, biological replicates. (**C**) Average distance to nearest neighbor cells in the invaginating E11 feather follicle. The bold cyan line indicates the median value of presumptive dermal papilla cells. Representative result. $n = 3/3$, biological replicates. (**D**) Relative cell density at proximal, distal, DP or upper bulge regions of the feather bud in E9, E10, and E11 feather bud. $n = 3/3$, biological replicates.

 © The Author(s)
